# Exploration of Phytochemical Constituents in *Neoshirakia japonica* by LC-MS and In Silico Evaluation of a New Antioxidant Targeting the NOX Family

**DOI:** 10.3390/antiox15081023

**Published:** 2026-08-17

**Authors:** Kyeong Seon Lee, Suzy Kim, Le Ba Vinh, Jae Sang Han, Bang Yeon Hwang, Mi Kyeong Lee, Ki Yong Lee

**Affiliations:** 1 College of Pharmacy, Korea University, Sejong 30019, Republic of Korea; kslee0118@korea.ac.kr (K.S.L.); wing_33@korea.ac.kr (S.K.); vinhrooney@gmail.com (L.B.V.); 2 Interdisciplinary Major Program in Innovative Pharmaceutical Sciences, Korea University, Sejong 30019, Republic of Korea; 3 Department of Chemistry, University of Bergen, 5007 Bergen, Norway; 4 College of Pharmacy, Chungbuk National University, Cheongju 28160, Republic of Korea; han00768@hanmail.net (J.S.H.); byhwang@chungbuk.ac.kr (B.Y.H.); mklee@chungbuk.ac.kr (M.K.L.)

**Keywords:** *Neoshirakia japonica*, LC-MS, chemical profiling, FBMN, ginsenoside, antioxidant, NOX family

## Abstract

*Neoshirakia japonica*, a small deciduous tree belonging to the family Euphorbiaceae, has traditionally been used to relieve pain and fatigue in ancient Asia. To date, there have been no attempts to investigate the overall metabolite profiles of *N. japonica*. To obtain the phytochemical profiles of its aerial part and root, the extracts were analyzed by UHPLC Orbitrap mass spectrometry, and the metabolites were clustered using feature-based molecular networking (FBMN). The common major constituents of the two different parts were annotated as ellagitannins and ellagic acid derivatives using the GNPS database and SIRIUS software, and a total of 20 compounds were isolated. With FBMN guidance, five minor ginsenosides (**10**–**14**) were isolated from the BuOH fraction of the aerial part and first identified in this plant. Also, one new xanthoxylin derivative (**16**) was discovered from the root, and its antioxidant activity and binding potential to NOX family proteins were evaluated by in silico studies. Consequently, this research aims to provide a comprehensive understanding of the major and minor components of *N. japonica* by part and suggests its potential as a drug candidate for antioxidant-related diseases.

## 1. Introduction

Oxidative stress is a harmful condition caused by an imbalance between free radical accumulation and cellular antioxidant functions. It can be triggered by various physiological and environmental factors such as overfatigue, stress, viral infections, and exposure to pollution or hazardous chemicals [1]. Under oxidative stress, highly unstable free radicals called ROS (reactive oxygen species) are generated and accumulated. Overproduction of ROS leads to damage to cell membranes and consequently contributes to human diseases like cancer, chronic inflammation, metabolic disease, or neurodegenerative diseases [2]. Therefore, antioxidants have important roles in reducing oxidative stress and maintaining healthy conditions.

NADPH oxidase (NOX) family is known as one of the major sources inducing ROS generation [3]. NOX is a transmembrane multienzyme complex composed of seven isomers: NOX1, NOX2, NOX3, NOX4, NOX5, DUOX1, and DUOX2 [4,5]. Among natural products, apocynin, berberine, EGCG, emodin, and resveratrol were suggested as promising NOX modulators [6], and strong binding potential between apocynin and NOX2 was demonstrated through in silico studies [7]. Also, studies evaluating the NOX inhibitory activity of structurally modified natural phenolic compounds have been steadily conducted [8].

*Neoshirakia japonica* (Siebold & Zuccarini) Esser, which belongs to the family Euphorbiaceae, is a perennial deciduous tree reaching an average height of 15 to 20 m, widely distributed in East Asia including Korea, China, and Japan [9]. Its original name was *Sapium japonicum*, but classified into the genus *Neoshirakia* later, and the name was revised to *Neoshirakia japonica* (*N. japonica*) [10]. The family Euphorbiaceae is a large spurge group consisting of over 8000 plant species worldwide [11,12]. Its main constituents were known as diterpenoids, sesquiterpenoids, and lupane- and oleanane-type triterpenoids. Among triterpenoids, new dammarane-type triterpenes were discovered recently [13]. Also, phenols, flavonoids, sterols, and tannins have been reported from various species in the Euphorbiaceae family [14,15,16]. In dried stems and leaves of *N. japonica*, coumarins, flavonoids, phenolic acids, and ellagitannins such as corilagin were reported as major constituents [17,18].

Traditionally, the leaves and root barks of *N. japonica* were used as folk remedies to relieve pain in the lower back and knee, overstrain and fatigue [19]. To date, studies on the discovery of bioactive chemical components have been steadily conducted. For example, 12-O-n-deca-2,4,6-trienoyl-phorbol-(13)-acetate and methyl 8-hydroxy-5,6-octadienoate were purified and exhibited piscicidal and anti-fungal activities [20,21]. From 2021 to 2026, new sesquineolignans, flavonol glycosides, phenylpropanoid glycosides, and casbane diterpenoids showing good anti-inflammatory activities in LPS-stimulated BV2 cells have been discovered by the same authors [22,23,24]. Also, the methanolic extract of *N. japonica* fruits, in which kaempferol is the main component, alleviated adipocyte inflammation and regulated adipogenic differentiation [25].

Nowadays, LC-MS analysis has become a crucial tool for the identification of natural product metabolites. Its sensitivity, high throughput, and high resolution enable the comprehensive analysis of complex phytochemical profiles [26,27]. Also, various LC-MS-based metabolite annotation tools such as Global Natural Products Social Molecular Networking (GNPS), SIRIUS, MS-finder, and Compound Discoverer have been developed for rapid molecular identification [28,29,30,31]. This approach accelerates discovery of minor components and targeted search for new metabolites, increasing the efficiency of natural product studies.

To date, various compounds have been reported to be isolated in *N. japonica*; however, a comprehensive phytochemical analysis has not yet been conducted. Therefore, the primary goal of this study was to gain an integrative overview of the distribution of chemical constituents in the root and aerial part of *N. japonica* and to discover previously missed or new bioactive metabolites by using LC-MS databases. In addition, by evaluating the antioxidant activities of *N. japonica* extracts and a new phenolic compound, we intended to propose a new antioxidant candidate for future drug development.

## 2. Materials and Methods

### 2.1. Reagents and Materials

For the evaluation of antioxidant activity, 2,2-diphenyl-1-picrylhydrazyl (DPPH), potassium persulfate, L-ascorbic acid, and Trolox were purchased from Sigma-Aldrich (St. Louis, MO, USA). 2,2′-Azino-bis(3-ethylbenzothiazoline-6-sulfonic acid) diammonium salt (ABTS) powder was obtained from Roche (Basel, Switzerland). As NMR solvents, D_2_O, CD_3_OD, CDCl_3_, DMSO-d_6_, acetone-d_6_, and pyridine-d_5_ were used (Cambridge Isotope Laboratories, Tewksbury, MA, USA). As standard references for comparing the optical rotation of sugar, α-_D_-glucose and (-)-_L_-glucose purchased from Sigma-Aldrich (USA) were used. HPLC-grade water and acetonitrile were bought from Fisher Scientific Korea Ltd. (Seoul, Republic of Korea). For open column chromatography, silica gel with particles of 0.040–0.063 mm (Millipore, MA, USA), ODS-C18 (YMC, 12 nm, S-150 μm), and Sephadex LH-20 resin (Supelco, Bellefonte, PA, USA) were used. For thin-layer chromatography (TLC), TLC silica gel 60 F254 and 60 RP-18 F254S were purchased from Supelco (USA), and 10% H_2_SO_4_ solution was prepared for the visualization of TLC spots. The microplate spectrophotometer (Agilent, Santa Clara, CA, USA) was used to measure the absorbance at 517 and 718 nm. The 1D and 2D NMR spectra were recorded using Bruker AVANCE III at 600 and 800 MHz (Bruker Co., Ltd., Billerica, MA, USA). Vanquish UHPLC system with an Orbitrap Exploris 120 mass spectrometer (Thermo Fisher, Waltham, MA, USA) was used to obtain HRESI-MS spectra. For measuring optical rotation, polarimeter P8000 (KRÜSS OPTRONIC, Hamburg, Germany) was used with a 50.0 mm cell.

### 2.2. Plant Materials

The aerial part and root of *N. japonica* were collected from Seoul National University’s medicinal plant garden (Goyang, Gyeonggi-do, Republic of Korea) in July 2023. The specimens were identified by Prof. Ki Yong Lee from the College of Pharmacy, Korea University (Sejong, Republic of Korea) and dried at room temperature. The voucher specimens (aerial part: KUP-HD-127; root: KUP-HD-128) were deposited at the Laboratory of Pharmacognosy in the College of Pharmacy, Korea University.

### 2.3. Extraction and Fractionation of N. japonica

The dried aerial part of *N. japonica* (3 kg) was chopped finely and ultrasonically extracted with 15 L of 80% MeOH for 90 min at 40 °C, and this was repeated four times. The 80% MeOH extracts were filtered, and the solvents were concentrated by a rotary vacuum evaporator, yielding 186.19 g (6.21%). Subsequently, the extracts were suspended in 1.5 L of distilled water and fractionated into *n*-hexane, EtOAc, and *n*-BuOH, respectively. The root of *N. japonica* (160 g) was cut into pieces and extracted with 100% MeOH (2 L) for 90 min using ultrasonication at 40 °C, and this was repeated three times. The 100% MeOH extracts underwent filtration, and the solvents were evaporated; the total yield was 9.13 g (5.71%). The extraction yields of extracts and solvent fractions are shown in Table S2.

### 2.4. LC-MS Data Processing of N. japonica Extracts and Fractions

#### 2.4.1. Sample Preparation and LC-MS Analysis

LC-MS samples were prepared at a concentration of 1 mg/mL, dissolved in 100% HPLC-grade MeOH and filtered through a 0.45 μm PVDF membrane. Five-microliter volumes of the samples were injected and separated by an analytical C18 column (OSAKA SODA Capcell Pak, 4.6 mm × 150 mm, 5.0 μm) at a column temperature of 20 °C. The gradient solvent conditions of HPLC-grade water (0.1% formic acid) and ACN (0.1% formic acid) were used as follows: 0–5 min, ACN 5%; 5–30 min, ACN 5–95%; and 30–40 min, ACN 95%. The flow rate was 0.6 mL/min, and a PDA detector was used for recording UV chromatograms. The parameters for HRESI-MS analysis were set up as follows: spray voltage for positive ion mode = 3500 V, and that for negative mode = 2500 V; gas pressures (arb) for sheath gas = 50, auxiliary gas = 15, and sweep gas = 1; the temperature of the ion transfer tube was 320 °C and that of the vaporizer was 275 °C; resolution of MS1 was 60,000 and of MS2 was 15,000; MS scan range (*m*/*z*) was 100–2000; and 30% normalized HCD was used for efficient fragmentation.

#### 2.4.2. Global Natural Social Molecular Networking (GNPS)

The .d data files of *N. japonica* extracts and solvent subfractions were converted into .mzML files using MSconvertGUI. The resulting .mzML files were processed by MZmine 4.3.8 to create GNPS-compatible datasets, and the following settings were used. In the mzwizard tab, UHPLC-Orbitrap-DDA was selected to perform LC-MS data processing. For the data processing of LC-MS data of aerial part and root extracts, the noise threshold level for MS1 was kept at 5.0 × 10^2^ and for MS2 at 5.0 × 10^1^. For the LC-MS data processing of aerial part solvent fractions, MS1 was kept at 3.0 × 10^2^ and MS2 at 3.0 × 10^1^. The retention time tolerance between samples was set to 0.10 min. The tolerance of *m*/*z* fluctuations between samples was 0.0015 *m*/*z*. After processing was finished, the created mgf. and csv. file was uploaded subsequently to the GNPS platform (http://gnps.ucsd.edu, accessed on 4 December 2023) to build a feature-based molecular network (FBMN). The minimum cosine score and minimum matched fragment ions were set to 0.7 and 4 for the FBMN analysis of the two extracts, and to 0.7 and 6 for the FBMN of the solvent fractions, respectively. The parameter settings were selected based on a balance between spectral matching specificity, reliable clustering of similar structures, and sufficient numbers of GNPS library hits. Precursor ion mass tolerance was 0.02 Da. Visualization of resulting networks was performed using Cytoscape 3.8.2.

#### 2.4.3. SIRIUS 6.3.3

During MZmine 4.3.8 processing, sirius.mgf files were created and imported to SIRIUS 6.3.3 software to annotate phytochemical constituents in the extracts and fractions. To compute all the datasets, the instrument option was selected as Orbitrap, and MS2 accuracy was set below 5 ppm. As fallback adducts, [M − H]^−^, [M + Cl]^−^, and [M + CH_2_O_2_ − H]^−^ were selected. For molecular formula identification based on isotope patterns, a de novo and bottom-up strategy was applied under the condition of performing de novo below 400 *m*/*z*. CSI:FingerID prediction was used to predict the molecular fingerprints from MS/MS and fragmentation trees, and compound class categorization was conducted using CANOPUS. MSNovelist was applied to generate de novo structures from combining MS/MS fragments.

### 2.5. Isolation Process of N. japonica

The EtOAc fraction (27.44 g) of the aerial part of *N. japonica* was subjected to silica gel column chromatography (CC) with a gradient of hexane/acetone (100:1→1:1, *v*/*v*) and CHCl_3_/MeOH (20:1→2:1, *v*/*v*), resulting in 11 subfractions (NJ-E1 to NJ-E11). NJ-E3 fraction was divided into 20 subfractions by C18 CC with acetone/H_2_O (2:1→8:1, *v*/*v*), and compounds **7** (52.4 mg) and **9** (75.3 mg) were obtained from NJ-E3-15 and NJ-E3-18 fractions. NJ-E3-19 fraction was eluted with acetone/H_2_O (6:1→8:1, *v*/*v*) by C18 CC, resulting in 8 subfractions (NJ-E3-19-1 to NJ-E3-19-8). From the NJ-E3-19-8 fraction, compound **8** (9.3 mg) was obtained. NJ-E4 fraction was applied to C18 CC with acetone/H_2_O (2:1→8:1, *v*/*v*), yielding 12 subfractions (NJ-E4-1 to E4-12), and compound **6** (110.7 mg) was obtained from NJ-E4-11 fraction. NJ-E6 fraction passed through silica gel CC with a CHCl_3_/MeOH (100:1→2:1, *v*/*v*), yielding 9 subfractions (NJ-E6-1 to NJ-E6-9). NJ-E6-5 was eluted with acetone/H_2_O (1:3→3:1, *v*/*v*) by C18 CC, and compound **4** (4.1 mg) was isolated. NJ-E8 fraction was applied to silica gel CC with CHCl_3_/MeOH/H_2_O (15:1:0.01→2:1:0.01, *v*/*v*), and 12 subfractions (NJ-E8-1 to NJ-E8-12) were obtained. NJ-E8-9 subfraction underwent C18 CC with MeOH/H_2_O (1:3→1:1, *v*/*v*), and compound **3** (2.7 mg) was obtained. NJ-E9 fraction was subjected to C18 CC with acetone/H_2_O (1:4→3:1, *v*/*v*), resulting in 12 subfractions (NJ-E9-1 to NJ-E9-12). From NJ-E9-8, compound **2** (170.4 mg) was obtained from the NJ-E9-8-8 fraction through Sephadex LH-20 CC with 100% MeOH. The NJ-E9-8-10 fraction was run through C18 CC with MeOH/H_2_O (1:7, *v*/*v*) to yield compound **1** (2.8 mg). NJ-E10 was eluted through C18 CC with MeOH/H_2_O (1:5→3:1, *v*/*v*), yielding 19 subfractions (NJ-E10-1 to NJ-E10-19), and compound **5** (7.8 mg) was purified from the NJ-E10-11 fraction by C18 CC with acetone/H_2_O (1:1, *v*/*v*).

The *n*-BuOH fraction (35.39 g) of the aerial part of *N. japonica* underwent DIAION HP20 CC with a step gradient of MeOH/H_2_O (0:100, 20:80, 40:60, 60:40, 80:20, 100:0, *v*/*v*), yielding 7 subfractions from NJ-B-1 to NJ-B-7. The NJ-B-6 fraction was subjected to silica gel cc with CHCl_3_/MeOH/H_2_O (12:1:0.01→2:1:0.01, *v*/*v*) to obtain 17 subfractions (NJ-B-6-1 to NJ-B-6-17). The NJ-B-6-17 subfraction was passed through C18 CC with acetone/H_2_O (1:3→1:1, *v*/*v*), and compounds **10** (14.8 mg), **11** (21 mg), **12** (8.7 mg), **13** (3.3 mg) and **14** (1.3 mg) were obtained.

The root extract of *N. japonica* was eluted with C18 CC with acetone/H_2_O (1:20→10:1, *v*/*v*), resulting in eight subfractions (NJR-1 to NJR-8), and compound **15** (2.81 g) was obtained from the NJR-4 subfraction. The NJR-5 fraction underwent silica gel CC with CHCl_3_/MeOH/H_2_O (3.5:1:0.01→1:1:0.01, *v*/*v*) yielding 10 subfractions (NJR-5-1 to NJR-5-10), and the NJR-5-3 fraction was applied to C18 CC with acetone/H_2_O (1:3.5→1:1, *v*/*v*), and compounds **16** (41.2 mg) and **20** (21.0 mg) were obtained. A gradient flow of CHCl_3_/MeOH/H_2_O (5:1:0.01→2:1:0.01, *v*/*v*) was applied to the NJR-5-7 fraction through silica gel CC, and compound **18** (10.4 mg) was obtained from the NJR-5-7-5 fraction. The NJR-5-1 fraction was passed through C18 CC with acetone/H_2_O (1:3.5→1:1, *v*/*v*), and compound **17** (14.3 mg) was obtained from the NJR-5-1-12 subfraction. Compound **19** (15.7 mg) was purified from the NJR-5-1-14 subfraction by silica gel CC with hexane/acetone (4:1→2:1, *v*/*v*). The extraction yields, isolation scheme and all spectroscopic data are available in Figures S1 and S6–S49.

Ginsenoside Ro (**10**): White amorphous powder; for ^1^H NMR (800 MHz, CD_3_OD) and ^13^C NMR (200 MHz), see Tables S1 and S2; ESIMS *m*/*z* = 955.4995 [M − H]^−^ (calculated for C_48_H_75_O_19_, 955.4994).

Ginsenoside Rb1 (**11**): White amorphous powder; for 1H NMR (600 MHz, CD_3_OD) and 13C NMR (150 MHz), see Tables S1 and S2; ESIMS *m*/*z* = 1107.6503 [M − H]^−^ (calculated for C_54_H_91_O_23_, 1107.6047).

Ginsenoside Rc (**12**): White needle-like crystal; ^1^H NMR (600 MHz, CD_3_OD) and ^13^C NMR (150 MHz), see Tables S1 and S2; ESIMS *m/z =* 1077.5950 [M − H]^−^ (calculated for C_53_H_89_O_22_, 1077.5950).

Ginsenoside Rb2 (**13**): White amorphous powder; for ^1^H NMR (600 MHz, CD_3_OD) and ^13^C NMR (150 MHz), see Tables S1 and S2; ESIMS *m*/*z* = 1077.5962 [M − H]^−^ (calculated for C_53_H_89_O_22_, 1077.5954).

Notoginsenoside R4 (**14**): Brown amorphous powder; for ^1^H NMR (800 MHz, pyr-d_5_) and ^13^C NMR (200 MHz), see Tables S1 and S2; ESIMS *m*/*z* = 1239.6509 [M − H]^−^ (calculated for C_58_H_97_O_17_, 1239.6511).

Neoshioside A (**16**): Brown amorphous powder; for UV (MeOH) λmax 254, 365 nm, ^1^H NMR (600 MHz, CD_3_OD + D_2_O) and ^13^C NMR (150 MHz), see Table 1; ESIMS *m*/*z* = 509.1298 [M − H]^−^ (calculated for C_23_H_25_O_13_, 509.1290).

### 2.6. Acid Hydrolysis of Compound ***16*** and Sugar Identification

Compound **16** (2.0 mg) was dissolved in 2 mL of 1 M aqueous HCl and heated at 80 °C for 4 h. After cooling down, the reaction mixture was partitioned with 2 mL of EtOA_C_ three times. The aqueous layer was collected and evaporated to dryness, and the residue was solubilized in water. The hydrolyzed sugar in the residue was confirmed by TLC, and its specific rotation was measured. The configuration of the sugar was identified by comparing the optical rotation with that of standard _D_-glucose.

### 2.7. DPPH Assay

A 120 μM DPPH solution was prepared by dissolving DPPH powder in 100% ethanol. The dried powder of samples was dissolved in ethanol (1% DMSO) and prepared into several final concentrations. As a negative control, 1% DMSO in ethanol was used, and L-ascorbic acid was used as a positive control, with a final concentration of 25 μM. Ten-microliter volumes of samples of each concentration were loaded into a 96-well plate, and 190 μL of DPPH solution was subsequently added. After incubating the plate for 20 min at room temperature in a dark room, the absorbance changes were measured at 517 nm using a microplate spectrophotometer. The radical scavenging activity was calculated as follows: DPPH radical scavenging activity (%) = {(OD of negative control − OD of sample-treated)/(OD of negative control)} × 100.

### 2.8. ABTS Assay

A 7 mM ABTS solution and a 2.4 mM potassium persulfate solution were prepared in distilled water and mixed in a ratio of 1:1. The mixed solution was incubated at room temperature in a dark room overnight. After incubation, the mixed ABTS solution was diluted with distilled water, and the absorbance was measured at 718 nm to ensure that the absorbance was 0.7–0.8. The samples were prepared in distilled water with 1% DMSO added at several concentrations. Furthermore, 1% DMSO was used as a negative control, and 20 μM Trolox was used as a positive control. Twenty-microliter volumes of samples were loaded into a 96-well plate, 180 μL of the mixed ABTS solution was added, and the plate was incubated for 7 min at room temperature in a dark room. After incubation, the absorbance changes were measured at 718 nm, and the radical scavenging activity was calculated as follows: ABTS radical scavenging activity (%) = {(OD of negative control − OD of sample-treated)/(OD of negative control)} × 100.

### 2.9. Molecular Docking Analysis

In silico studies were conducted using Maestro 14.7 (2026-1) from Schrödinger (New York, NY, USA). The PDB structures of NOX2 (7U8G) and NOX5 (5O0X) were downloaded from the Protein Data Bank (http://www.rcsb.org, accessed on 30 April 2026). The protein structures were preprocessed by the Protein Preparation Workflow, and ligand binding sites were designated by Receptor Grid Generation. The grid box was generated at the centroid of the ligand at X137.35_Y133.65_Z154.5 for NOX2 and X70.11_Y-2.83_Z62.76 for NOX5 with a size of 20 Å. The possible ionization forms of ligands were generated at pH 7.00 ± 2.00 through LigPrep in the OPLS4 force field. Prepared ligands were docked against the target proteins using the XP docking mode. The validation of the docking protocol was performed as follows. The co-crystallized ligand was first removed from the protein structure, and redocking was then carried out to reproduce its binding pose at the original binding site. RMSD between the redocked pose and the crystallographic pose was less than 2.0 Å, so the protocol was considered validated.

### 2.10. Molecular Dynamics Simulation

Molecular dynamics simulation was performed using Maestro Desmond. The environmental condition replicating a biological system was set up using System Builder. The Simple Point Charge (SPC) solvent model and OPLS force field were selected to calculate intermolecular interactions under water conditions. The protein complex was placed inside an orthorhombic box with a distance of 10 Å. Additionally, 4 Na^+^ ions and 0.15 M NaCl were added for neutralization. For MD analysis, the NPT ensemble class was chosen. Simulation time, temperature, and pressure were set as 100 ns, 300 K, and 1.01 bar, respectively. The model system was ensured to be relaxed before simulation to resolve steric clashes and equilibrate the environment. RMSD, RMSF, and protein–ligand contacts were monitored and reported as simulation diagrams.

## 3. Results

### 3.1. LC-MS Analysis of Major Constituents from the Aerial Part and Root of N. japonica

**Figure 1:**
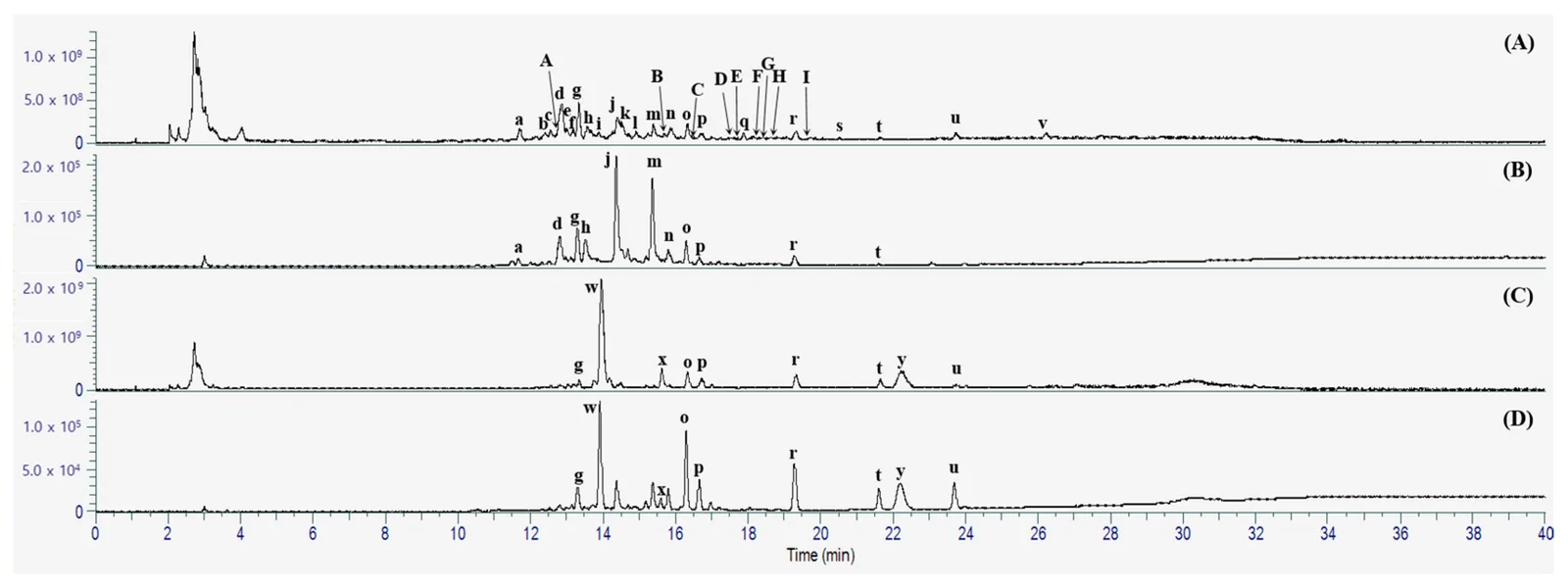
The LC-MS analysis of the aerial part and root of *N. japonica*: (**A**) the ESIMS chromatogram of the aerial part extract in negative mode; (**B**) UV chromatogram of aerial part (254 nm); (**C**) the ESIMS chromatogram of the root extract in negative mode; (**D**) UV chromatogram of the root extract (254 nm).

**Figure 2:**
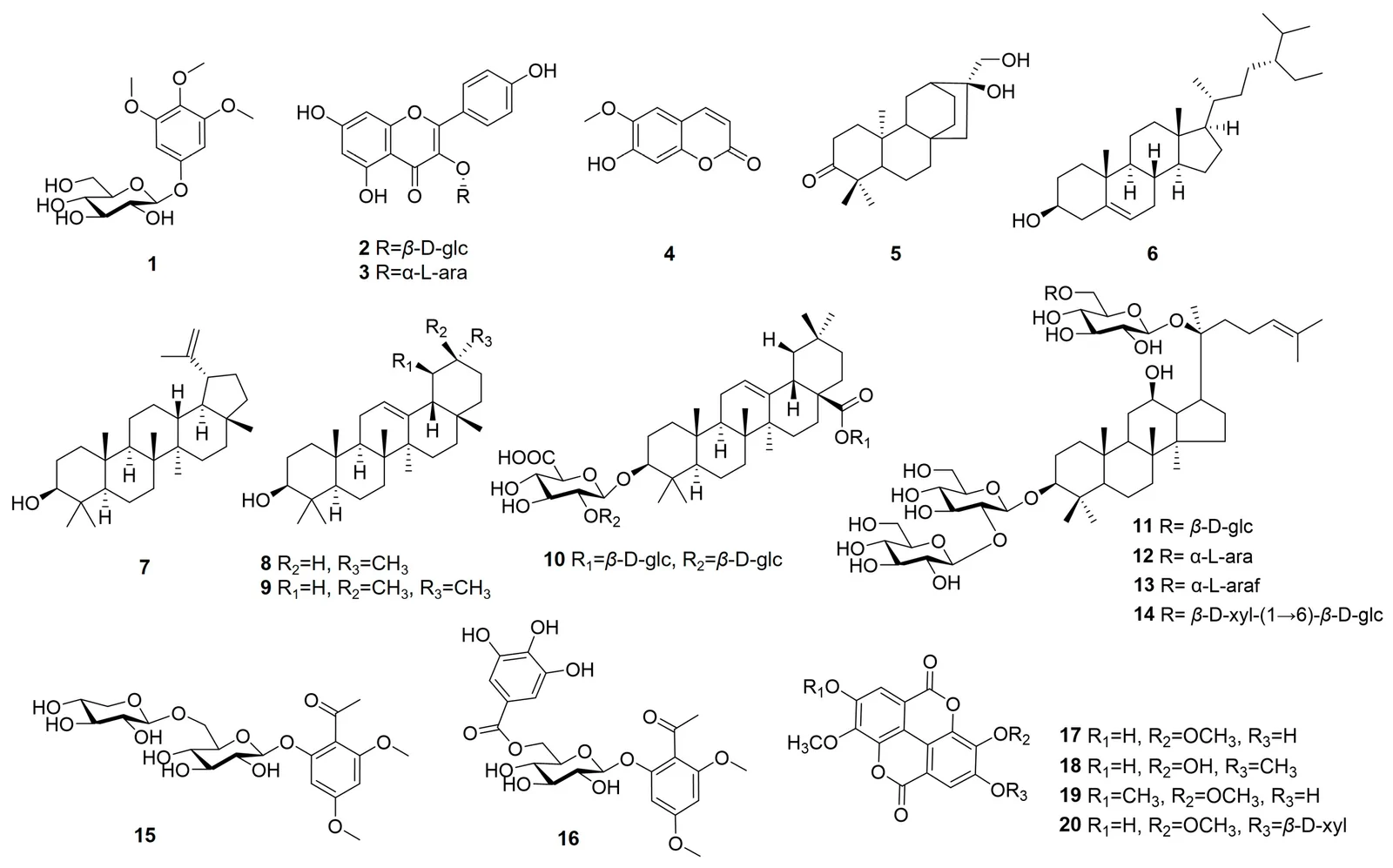
Isolated compounds from *N. japonica*.

The major peaks in MS and UV chromatograms were designated by lowercase letters (a–y), and minor constituents isolated through column chromatographic techniques were designated with arrows and capital letters, from A to I (Figure 1). The recorded data and annotations of components were listed in Table 1. Some constituents were tentatively annotated by LC-MS/MS databases such as GNPS, SIRIUS, and information reported in the literature. To show confidence levels of metabolite annotations, the system of metabolite identification confidence levels (MIC) was applied [32,33]. Using chromatographic techniques, a total of 20 compounds were isolated, and the corresponding peaks were identified in the chemical profile (Figure 2). The structures of known compounds were as follows. Nine compounds, i.e., 3,4,5-trimethoxyphenyl β-d-glucopyranoside (**1**), astragalin (**2**), juglalin (**3**), scopoletin (**4**), ent-atisane-3-one-16α-17-diol (**5**), β-sitosterol (**6**), lupeol (**7**), α-amyrin (**8**), and β-amyrin (**9**), were isolated from the EtOA_C_ fraction of the aerial part. Five saponins, i.e., ginsenoside Ro (**10**), ginsenoside Rb1 (**11**), ginsenoside Rc (**12**), ginsenoside Rb2 (**13**), and notoginsenoside R4 (**14**), were target-isolated from the *n*-BuOH fraction of the aerial part with the FBMN strategy and first identified in this species. From the root, one new compound neoshioside A (**16**) and five known compounds acetophenone-6′-xylosyl-(1-6)-glucopyranoside (**15**), 3,3′-di-O-methylellagic acid (**17**), 3,4′-di-O-methylellagic acid (**18**), 3,3′,4-tri-O-methylellagic acid (**19**), and 3,3′-di-O-methylellagic acid-4′-O-β-d-xylopyranose (**20**) were purified.

**Figure 3:**
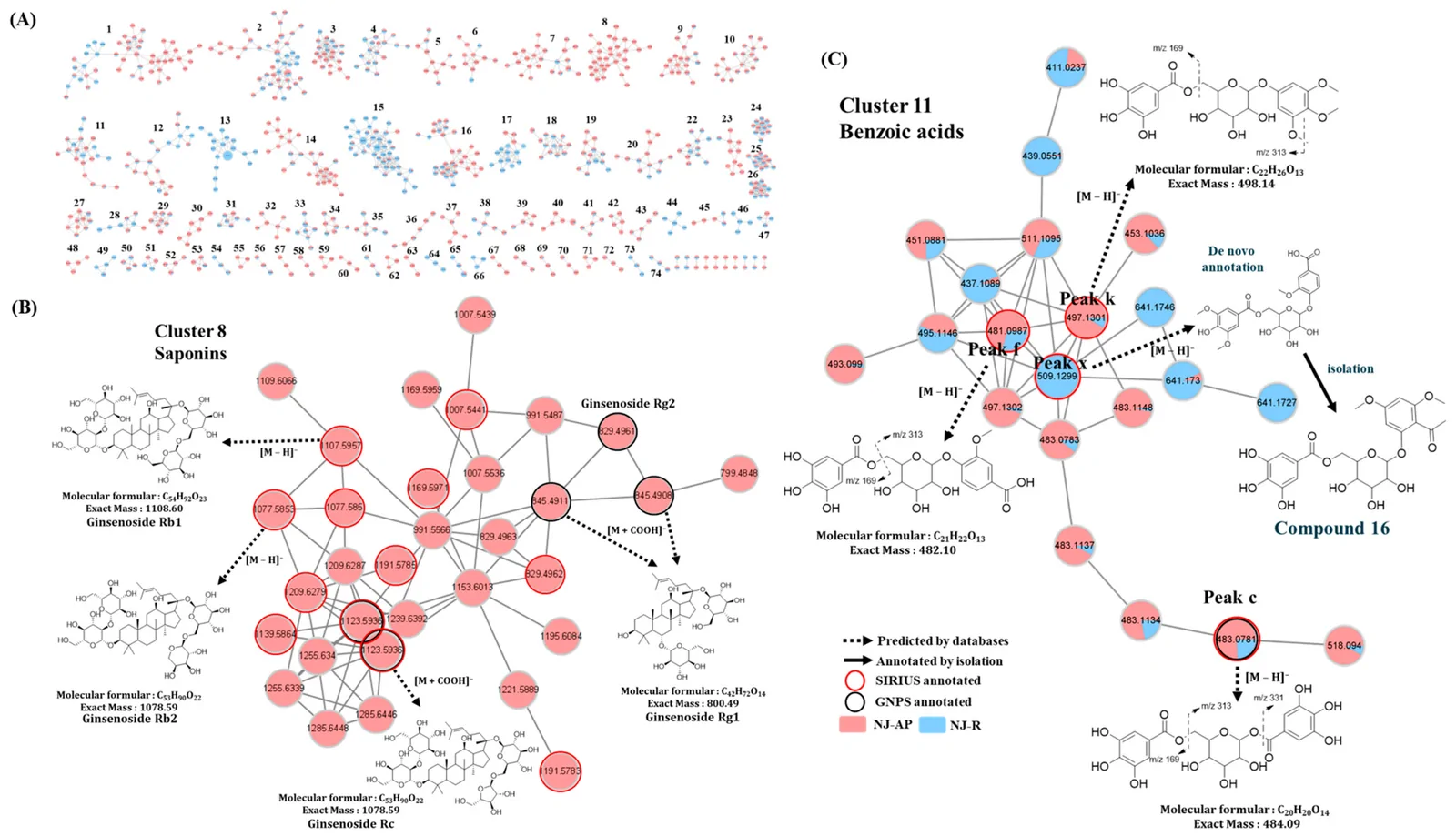
The FMBN of the aerial part and root of *N. japonica*: (**A**) 74 clusters with at least three nodes connected were made via GNPS feature-based molecular networking; (**B**) tentative annotation of ginsenosides from cluster 8; (**C**) putative prediction of molecular structures based on MS/MS fragments and identification of a novel compound from cluster 11.

The LC-MS data was processed by FBMN, and a total of 74 clusters with at least three nodes connected to each other were made (Figure 3A). The parameters of cosine score and minimum matched fragment ions used in FBMN were selected as 0.7 and 4, respectively, to ensure reliable spectral matching and sufficient library hits. The result of the parameter screening was provided in Table S1. Peaks a, d, and e were found in cluster 1, which consisted of phenolic acids and their glycosides. Using GNPS and SIRIUS software, peak a was annotated as chlorogenic acid, and peaks d and e were putatively predicted as its derivatives based on their MS/MS fragments at *m*/*z* 179 and 191 (Figure S1) [34]. In cluster 7, peaks g and h were tentatively predicted as corilagin and geraniin, respectively, based on the matching of the precursor ions and MS/MS fragments (Figure S2). Corilagin and geraniin have been reported as major components of *N. japonica* [17,18]. Loss of galloyl glucose and galloyl acid resulted in the fragments of *m*/*z* 301 and 463, which were consistent with the MS/MS fragment patterns in previously reported papers [35,36]. Other ellagitannins such as excoecariphenol were also predicted using SIRIUS. In cluster 8, the nodes with *m*/*z* values of 829.4961, 845.4908, and 1123.5936 were predicted as ginsenoside Rg2, Rg1, and Rc by GNPS and SIRIUS. Also, the nodes with *m*/*z* values of 1107.5957 and 1077.5835 were observed and expected to be ginsenoside Rb1 and Rb2 by SIRIUS (Figure 3B). These results suggested that dammarane-type saponins are distributed in the aerial part. In cluster 9, phenylpropanoids including flavonoids and ellagic acids were grouped together (Figure S3). The nodes corresponding to peak n (astragalin, compound **2**) and juglalin (compound **3**) were confirmed, while the trace of hyperin and rutin was predicted using GNPS and SIRIUS. The putatively assigned structure of peak j was ellagic acid glycoside based on its MS1 and fragment ion at *m*/*z* 301. Acetophenone derivatives from the root were found in cluster 13, and the largest node with an *m*/*z* value of 535.1668 corresponded to peak w, which was identified as compound **15** (Figure S4). In cluster 11, the nodes corresponding to peaks c, f, k, and x were identified (Figure 3B). Peaks c, f, and k were predicted as benzoic acid derivatives. Based on their fragment ions at *m*/*z* 169 and 313, galloyl glucosides and benzoic acid fragments were combined to match the MS1 precursor ions. The node corresponding to peak x, with *m*/*z* of 509.1299 and distributed mainly in the root, was initially tentatively annotated as apocynin-4-O-β-d-(6′-O-syringyl)glucopyranoside using the de novo annotation function of SIRIUS [37]. However, after the compound corresponding to peak x (compound **16**) was isolated by column chromatography, its structure was determined to be 6-galloyl glucoside linked to xanthoxylin, a previously undescribed one in nature.

### 3.2. Tracking of Dammarane-Type Saponins from the Aerial Part of N. japonica with FBMN Guidance

**Figure 4:**
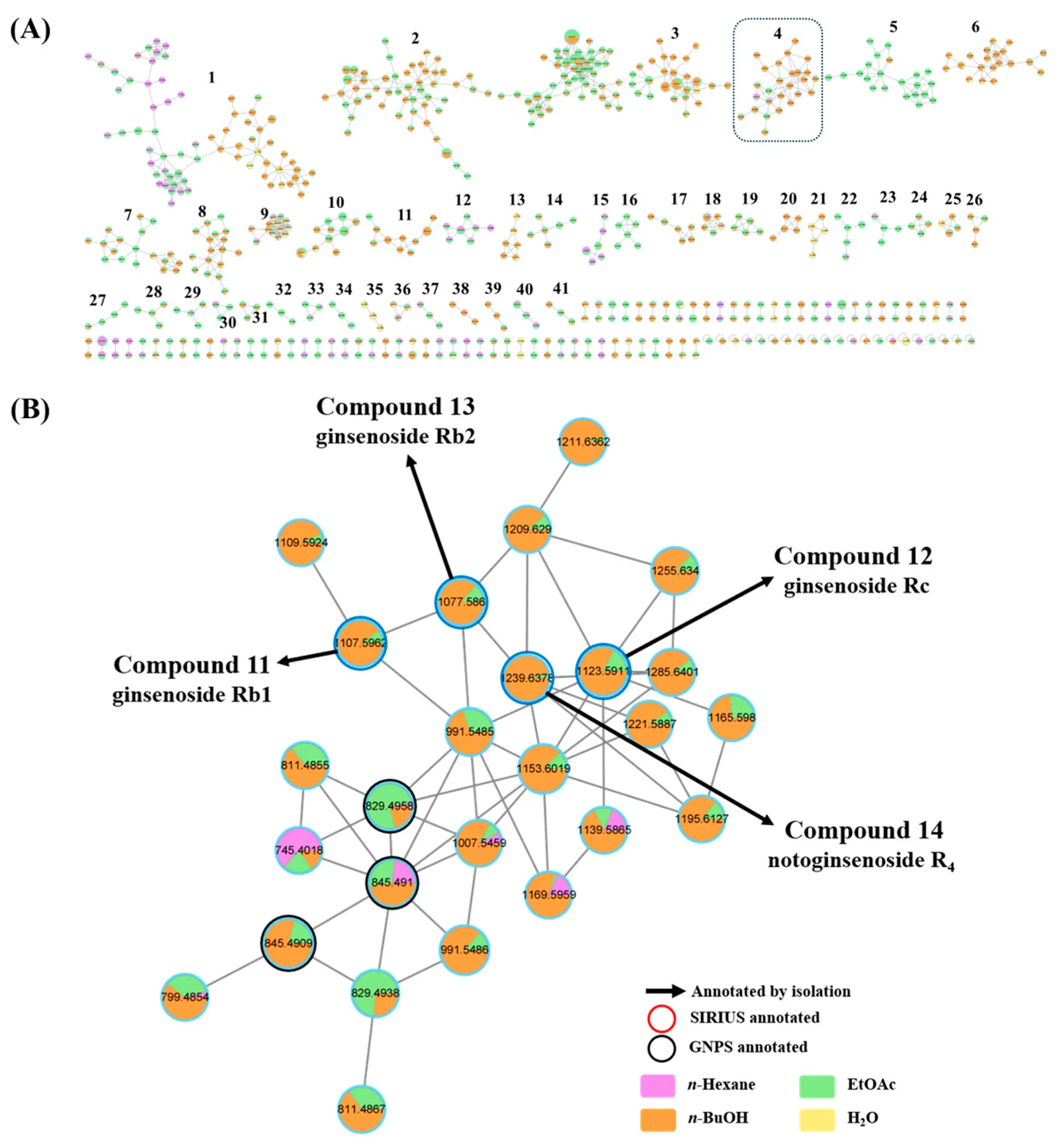
FBMN-guided ginsenosides isolation from the *n*-BuOH fraction of the aerial part of *N. japonica*: (**A**) 41 clusters with at least three nodes connected were formed by the FBMN; (**B**) finding the target saponins in the *n*-BuOH fraction and isolated nodes marked with black arrows.

To investigate which solvent fraction mainly contained the saponins, FBMN was performed on the *n*-hexane, EtOA_C_, *n*-BuOH, and H_2_O fractions of the aerial part, and a total of 41 clusters with at least three nodes connected were obtained. Among these 41 clusters, the target saponins were found in cluster 4 and mainly distributed in the *n*-BuOH fraction (Figure 4A). Guided by FBMN results, four dammarane-type saponins (**11**–**14**) were isolated from the *n*-BuOH fraction, and the corresponding nodes were designated with black arrows (Figure 4B). Additionally, one oleanane-type saponin, ginsenoside Ro (**10**), was isolated. The structures of five ginsenosides were determined by NMR and MS spectroscopy and carefully confirmed by comparison with previously reported NMR assignments of ginsenosides [38,39,40,41,42]. A detailed description of the structure elucidation can be found in Tables S3 and S4 and Figures S18–S38.

### 3.3. Identification of New Constituent from the Root of N. japonica

**Figure 5:**
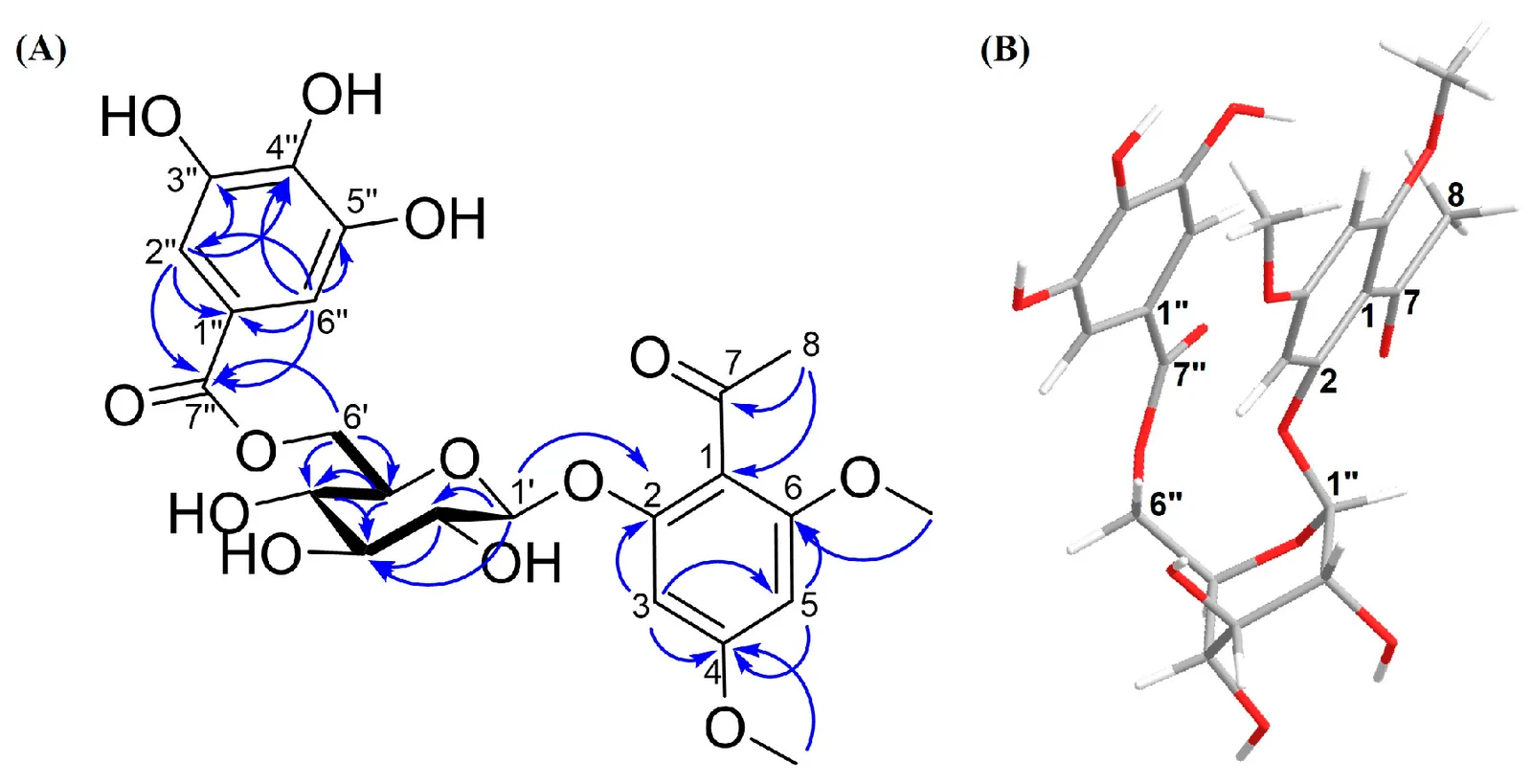
(**A**) The key HMBC (H→C) and ^1^H-^1^H COSY correlations of the novel compound **16**; (**B**) MM2-minimized 3D structure of compound **16** using Chem3D.

Compound **16** was obtained as an amorphous brown powder. The molecular formula was assigned as C_23_H_26_O_13_ based on its HRESIMS data, *m*/*z* of 509.1298 [M − H]^−^ (calculated for C_23_H_25_O_13_ 509.1290). The ^1^H-NMR spectrum included one methyl signal at δ_H_ 2.45, two methoxy signals at δ_H_ 3.64 and 3.75, four aromatic proton signals at δ_H_ 6.26, 6.34, 7.05, one anomeric proton at δ_H_ 4.91, and five oxymethine protons located in glucose at δ_H_ 3.41–4.58. ^13^C-NMR spectrum exhibited 23 carbon signals, including one carbonyl carbon at δ_C_ 196.2 and aromatic carbons. All protonated carbon signals were identified based on HSQC correlations. In the HMBC spectrum, the correlation from H-8 (δ_H_ 2.45) to C-7 (δ_C_ 196.2) and C-1 (δ_C_ 130.7) was observed, indicating the methyl group is bound to the carbonyl group and is located near the aromatic carbon. H-3 (δ_H_ 6.34) and H-5 (δ_H_ 6.26) were correlated to the surrounding aromatic carbons. The methoxy signal at δ_H_ 3.75 was assigned as 6-OCH_3_ due to its correlation to C-6 (δ_C_ 158.40), and another methoxy signal at δ_H_ 3.64 was determined as 4-OCH_3_ because correlation to C-4 (δ_C_ 162.89) was observed. This proves the presence of the xanthoxylin moiety, and the chemical shift values were consistent with the literature [43]. The ^1^H-NMR spectrum showed one anomeric proton at δ_H_ 4.91, indicating one sugar moiety, which was predicted as β-glucose based on its J value (*J* = 7.8 Hz). In the HMBC spectrum, the correlation peaks between sugar protons and carbons were observed: H-2′ (δ_H_ 3.43)/C-1′ (δ_C_ 101.87) and C-3′ (δ_C_ 76.36), H-3′ (δ_H_ 3.43)/C-4′ (δ_C_ 70.20), H-5′ (δ_H_ 4.42, 4.58)/C-3′(δ_C_ 76.36) and C-4′ (δ 70.20), and H-6′ (δ_H_ 4.42, 4.58)/C-4′ (δ 70.20). The connections between all sugar protons were confirmed via the COSY spectrum. In addition, the correlation peaks between H-1′ (δ_H_ 4.91)/H-3′ (δ_H_ 3.49)/H-5′ (δ_H_ 3.75) were observed in the NOESY spectrum, thus providing evidence of the presence of glucopyranose. Following acid hydrolysis, the sugar moiety was identified as _D_-glucose (${\left[a\right]}_{D}^{20}$ + 55.7, *c* 0.1) by comparison of its optical rotation with that of standard _D_-glucose and a previously reported paper [44]. Accordingly, the sugar moiety was determined to be β-_D_-glucopyranoside. Furthermore, the cross peak between H-1′ (δ_H_ 4.91) and C-2 (δ_C_ 156.01) was observed, suggesting that β-d-glucopyranoside was connected to C-2 of the xanthoxylin moiety. The chemical shifts in C-1″ (δ_C_ 119.93) to C-7″ (δ_C_ 166.85) were similar to the reported values of gallic acid, and the existence of the gallic acid moiety was confirmed by HMBC analysis [45,46]. The gallic acid moiety was linked to the glucopyranoside to form 6-galloyl glucoside, as evidenced by the HMBC correlation from H-6′ (δ_H_ 4.42/4.58) to C-7″ (δ_C_ 166.85) (Figure 5). Consequently, compound **16** was identified as xanthoxylin-2-O-(6-galloylglucoside). It has not previously been reported in nature and is named as neoshioside A. The NMR and HRESIMS spectra can be seen in Table 2 and Figures S40–S45.

### 3.4. Evaluation of Antioxidant Activities of N. japonica Extracts and a New Compound

To evaluate the antioxidant activity of extracts and the new compound, DPPH and ABTS assays were conducted. At five concentrations ranging from 1 to 200 μg/mL, both extracts showed good radical scavenging activities in a dose-dependent manner, and the aerial part exhibited slightly higher antioxidant activity than the root (Figure S5). This strong activity might be due to the main components of *N. japonica*. For example, corilagin and geraniin have been reported to exhibit strong DPPH radical scavenging activities [47,48], and chlorogenic acid and ellagic acid derivatives are well known as potent antioxidants [49,50,51]. Among isolated phenolic compounds, neoshioside A (**16**) showed the strongest antioxidant activity. In Table 3, neoshioside A (**16**) exhibited dose-dependent activity within the range of 1 to 100 μM. The IC_50_ values were approximately 43.7 μM in the DPPH assay and 15.8 μM in the ABTS assay. This might be due to methodological differences between the DPPH and ABTS assays, such as free radical solubility and detection wavelengths [52]. Consequently, compound **16** may contribute to the antioxidant activity of the root extract and could be considered a potential antioxidant constituent.

### 3.5. Molecular Docking of New Antioxidant Against NOX2 and NOX5

**Figure 6:**
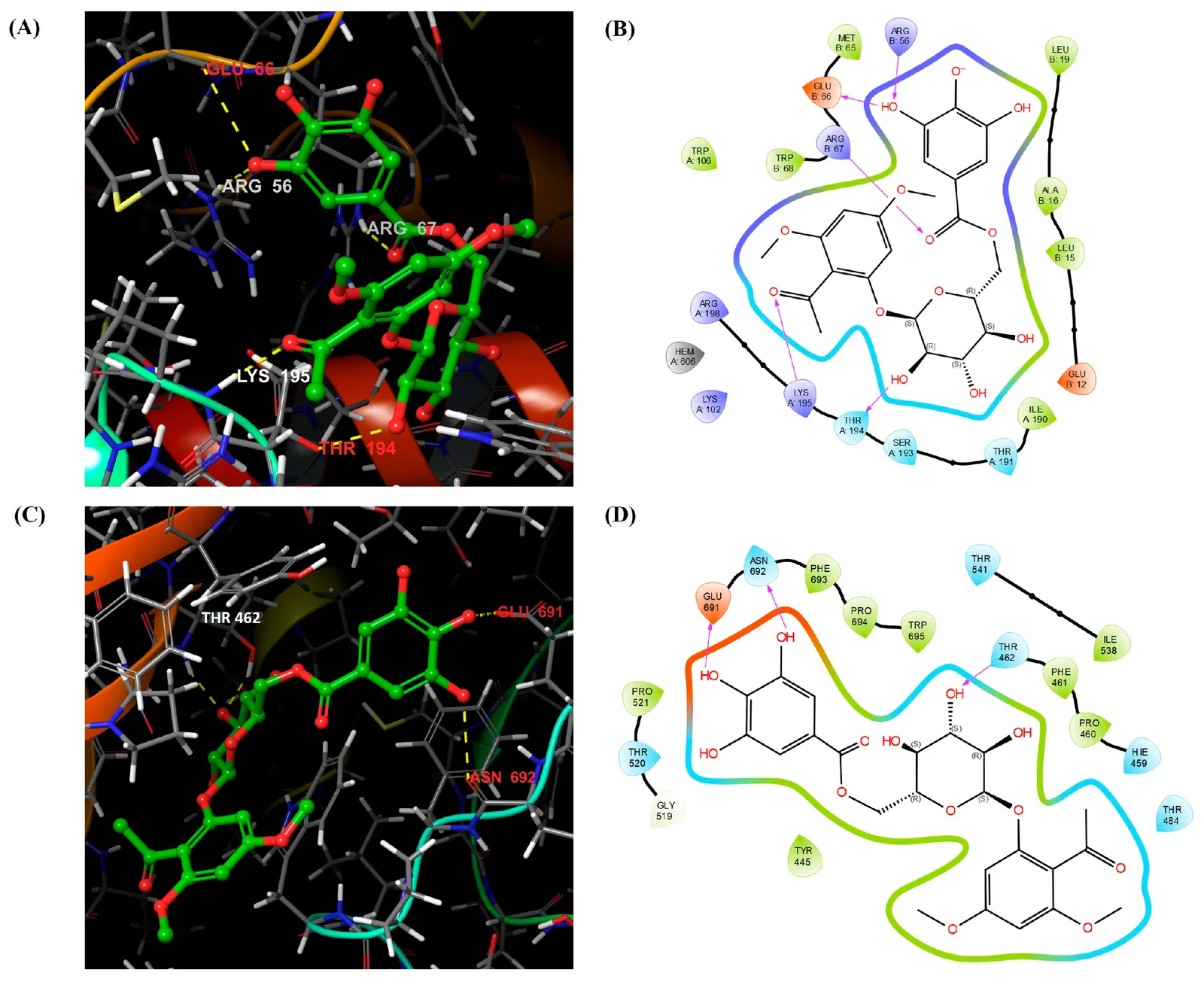
Molecular docking analysis: (**A**) 3D interaction of NOX2 with compound **16** and (**B**) 2D interaction diagram; (**C**) 3D interaction of NOX5 with compound **16** and (**D**) 2D interaction diagram.

Apocynin, which has a similar backbone structure to compound **16**, is well known as an inhibitor of the NOX family. Considering the structural similarity and antioxidant activity between the two compounds, molecular docking studies were performed to investigate the binding affinity of compound **16** against two ROS-generating targets, NOX2 and NOX5. The co-crystallized ligands and L-ascorbic acid were used as positive controls. Hydrogen-bonding interactions were formed between compound 16 and NOX2 via GLU66, ARG56, ARG67, THR194, and LYS195 residues (Figure 6). The XP docking score of compound **16** against NOX2 was −7.083 Kcal/mol, with a H-bond energy of −3.878 Kcal/mol, demonstrating favorable binding affinity (Table S5). Compound **16** formed hydrogen-bonding interactions with NOX5 through three residues, THR462, GLU691, and ASN692. The docking score against NOX5 was −11.534 Kcal/mol, indicating excellent binding affinity (Table S6). The interactions of compound **16** with two proteins were mainly dependent on hydrogen bonding. Low negative glide energy scores imply that compound **16** can form stable interactions with the binding pockets of target proteins, supporting its high potential to target the NOX family.

### 3.6. Molecular Dynamics Simulation of NOX5 and Ligand Complex

**Figure 7:**
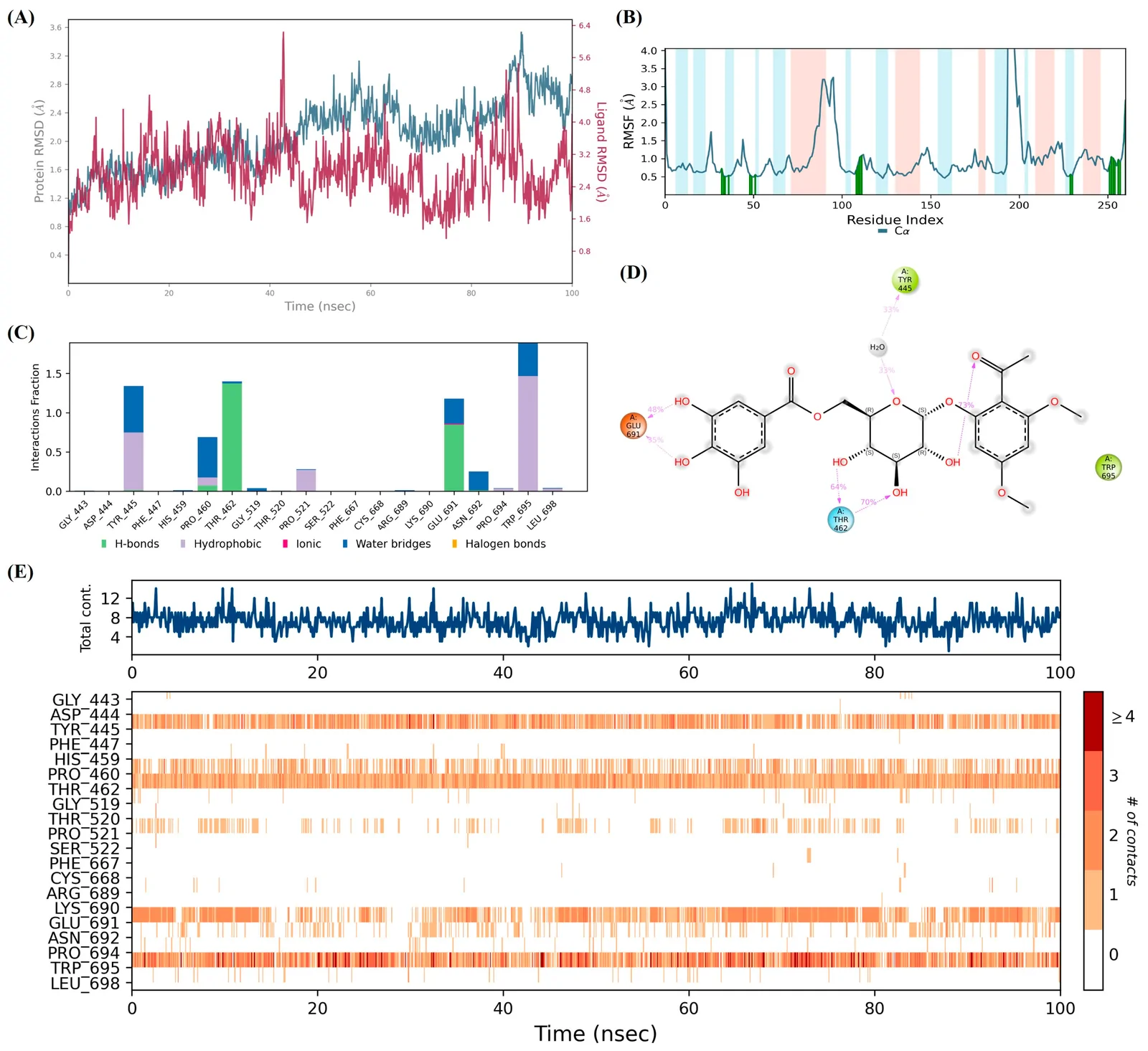
Molecular dynamics analysis of compound **16** against NOX5: (**A**) RMSD for the NOX5 protein and its ligand during 100 ns of a simulation run; (**B**) protein RMSF and the ligand-interacting residues (marked with green color); (**C**) protein–ligand contacts monitored throughout simulation; (**D**) the diagram of ligand atom interaction with NOX5 residues; (**E**) the trajectory of interactions.

In general, an XP docking score below −9 kcal/mol indicates excellent binding affinity, while scores between −9 and −7 kcal/mol and between −7 and −5 kcal/mol generally indicate good and moderate binding affinity, respectively [53,54]. Based on the docking score, the new compound was predicted to have a more favorable binding affinity towards NOX5 than towards NOX2. Therefore, molecular dynamics (MD) simulations were conducted for the NOX5–compound **16** complex to evaluate its binding stability, equilibrium state and conformation changes in protein–ligand complexes in a solvent system. During the simulation lasting 100 ns, the protein RMSD value remained between 1 and 3 Å, indicating that the NOX5 structure was relatively stable (Figure 7A). Although the ligand RMSD showed a temporary increase in fluctuation around 40 ns, this might indicate a rearrangement of the ligand binding pose in response to structural changes in the protein, as an increase in the protein RMSD was also observed around this time. After the protein–ligand complex reached an equilibrium state between 50 ns and 100 ns, the ligand RMSD decreased and remained below approximately 2~3 Å. These results suggest that the interaction between compound **16** and NOX5 remained quite stable after equilibration, with a temporary fluctuation finding an energetically favorable docking pose in a solvent system. To evaluate the structural fluctuations in NOX5, the RMSF values were analyzed (Figure 7B). The α-helix regions were colored pink, β-strands blue, and loops transparent. Compound **16** interacted with a total of 18 amino acid residues in NOX5 including THR462 and GLU691, and low RMSF values were observed at the contacted region: GLY443 (0.73 Å), ASP444 (0.53 Å), TYR445 (0.50 Å), PHE447 (0.54 Å), HIS459 (0.55 Å), PRO460 (0.51 Å), THR462 (0.56 Å), GLY519 (0.68 Å), THR520 (0.83 Å), PRO521 (1.04 Å), SER522 (1.08 Å), PHE667 (0.57 Å), CYS668 (0.53 Å), ARG689 (0.82 Å), LYS690 (1.05 Å), GLU691 (1.01 Å), PRO694 (0.98 Å), and TRP695 (0.91 Å). In Figure 7C,D, protein–ligand contacts were presented as diagrams. The interactions between NOX5 and compound **16** complexes were mainly mediated by hydrogen-bonding interactions involving the THR462 and GLU691 residues, consistent with the docking results. In addition, water bridges were formed between the TYR665 residue and the oxygen atom of the glucopyranoside in compound **16**, potentially enhancing the stability of the interaction. The detailed timeline of protein–ligand contacts during the simulation is presented in Figure 7E.

## 4. Discussion

Based on LC-MS analysis, major constituents were tentatively predicted as ellagitan-nins, ellagic acids, and benzoic acid derivatives. In the root part, the compositions were almost similar to those of the aerial part, but acetophenone derivatives (**15** and **16**) were specifically detected. Our study has limitations because the metabolite predictions relied primarily on database searches. Therefore, additional experiments might be required for precise metabolite identification and cross-validation using more diverse MS libraries. However, these results have implications for providing a standardized chemical profile for *N. japonica* for the first time. Using FBMN and MS databases, minor components that have previously been missed were found efficiently. Ginsenosides, famous for their various pharmacological activities such as immunomodulation and regulation of energy metabolism, were first discovered in this species and the Euphorbiaceae family by FBMN guidance. To date, the pharmacological activities of *N. japonica* have not been fully studied, and a few studies have reported its anti-inflammatory and anti-diabetic activities. However, by identifying the previously unreported compounds through our study, we believe that there are still many opportunities to explore the unique biological effects of this plant. Thus, our findings not only broaden the understanding of the chemical composition of this species but also might contribute to investigating its unrevealed pharmacological activities in the future.

Radical scavenging assays and MD simulations were carried out to evaluate the antioxidant activity of neoshioside A (**16**), a newly discovered compound. The results suggested compound **16** as a promising antioxidant with a good binding affinity for NOX family proteins. MD simulations were conducted for the NOX5–neoshioside A (**16**) complex to evaluate its binding stability based on its XP docking score. However, docking scores alone cannot fully explain the relative binding stability; therefore, further comparative evaluation of the binding stability of compound **16** towards different NOX isoforms may be needed. In addition, this study was based solely on computational analyses and has the limitation that the actual inhibitory activity of compound **16** against the NOX family could not be confirmed. Thus, additional experiments using cell-based and in vivo models should be conducted to confirm the inhibitory activity of the new compound against NOX proteins. NOX plays an important role in the cellular antioxidant system by regulating intracellular ROS levels. This study has the limitation that, while the free radical scavenging activity of compound **16** was confirmed, its involvement in the cellular NOX-mediated antioxidant mechanism has not been elucidated. Therefore, further investigations are needed to determine whether compound **16** can reduce ROS levels through NOX inhibition in disease models. In addition, although compound **16** formed a stable complex with NOX5 during MD simulation, further pharmacokinetics and pharmacodynamics experiments are required to evaluate its in vivo behavior and stability. Although its actual inhibitory activity against NOX proteins must be validated through additional experiments, our study implicates that neoshioside A (**16**) has the potential to serve as a promising candidate targeting the NOX family.

## 5. Conclusions

In this study, the major chemical constituents in the aerial part and root of *N. japonica* were predicted using MS/MS-based molecular networking combined with various chromatography techniques. Tannins, benzoic acids, coumarins, flavonoids, and terpenoids were identified, all of which have been reported as major constituents of the Euphorbiaceae family. Acetophenone derivatives, which have been reported in a limited number of Euphorbiaceae species, were detected from the root of *N. japonica* [55]. Saponins in the Euphorbiaceae family are predominantly oleanane- and lupane-type triterpenoid saponins, and dammarane-type saponins have not previously been reported. However, ginsenosides were identified for the first time in both this species and the Euphorbiaceae family, thereby expanding the understanding of the chemical diversity of *N. japonica*.

Furthermore, in silico analysis suggested that the new compound has the potential to target the NOX family and inhibit ROS-generating enzymes. Although its inhibitory activity against NOX proteins should be validated by further in vitro and in vivo studies, these findings indicate that *N. japonica* and its new compound can be utilized as therapeutic antioxidant candidates.

## Figures and Tables

**Table 1:** The phytochemical profile of the aerial part and root of *N. japonica*.

Peak No.	Expected Compounds	t_R_ (min)	Observed *m*/*z*	Calculated *m*/*z*	Molecular Formula [M − H]^−^	MS/MS Fragments (*m*/*z*)	UV (λmax, nm)	Comp. No.	Databases	MIC **
**a**	Chlorogenic acid	11.72	353.0870	353.0867	C_16_H_17_O_9_	191, 179	254, 365	-	GNPS, SIRIUS, the literature [17]	2
**b**	Benzoic acid derivatives	12.38	511.1094	511.1126	C_22_H_23_O_14_	467, 313, 169	254, 365	-	SIRIUS	3
**c**	3,4,5-trihydroxy-6-(3,4,5-trihydroxybenzoyl)oxyoxan-2-yl)methyl 3,4,5-trihydroxybenzoate	12.55	483.0779	483.0769	C_20_H_19_O_14_	331, 313, 169	280	-	GNPS, SIRIUS	3
**d**	Caffeoyl quinic acid derivatives	12.87	353.0871	353.0867	C_16_H_17_O_9_	191	254, 365	-	GNPS, SIRIUS	3
**e**	Caffeoyl quinic acid derivatives	13.02	353.0872	353.0867	C_16_H_17_O_9_	191, 179, 173	254, 365	-	GNPS, SIRIUS	3
**f**	3-methoxy-4-[3,4,5-trihydroxy-6-[(3,4,5-trihydroxybenzoyl)oxymethyl]oxan-2-yl]oxybenzoic acids	13.14	481.0986	481.0993	C_21_H_21_O_13_	313, 169	275	-	SIRIUS	3
**g**	Corilagin	13.33	633.0732	633.0749	C_27_H_21_O_18_	463, 301	254, 365	-	GNPS, SIRIUS, the literature [17]	2
**h**	Geraniin	13.57	951.0742	951.0765	C_41_H_27_O_27_	301	254, 365	-	SIRIUS, the literature [17,18]	2
**i**	Unidentified	13.69	433.2076	433.2068	C_20_H_33_O_10_ *	301	254, 365	-	-	-
**j**	Ellagic acid-4-O-xylopyranoside	14.41	433.0409	433.0402	C_19_H_13_O_12_	301	254, 365	-	SIRIUS	3
**k**	3,4,5-trihydroxy-6- (3,4,5-trimethoxyphenoxy)oxan-2-yl]methyl 3,4,5-trihydroxybenzoates	14.52	497.1300	497.1290	C_22_H_25_O_13_	313, 169	275	-	SIRIUS	3
**l**	Tetrahydroxyxanthones	14.92	305.0302	305.0292	C_14_H_9_O_8_ *	273, 245	254, 365	-	SIRIUS	4
**m**	Ellagic acid	15.39	301.0018	301.0012	C_14_H_5_O_8_	219	254, 365	-	GNPS, SIRIUS, the literature	2
**n**	Astragalin	15.89	447.0927	447.0922	C_21_H_19_O_11_	284	254, 365	2	GNPS, SIRIUS, the literature [17]	1
**o**	3, 3′-Di-O-methyellagic acid-4′-β-O-d-xylopyranoside	16.38	461.0722	461.0715	C_21_H_17_O_12_	446, 328, 313	254, 365	20	SIRIUS, the literature [18]	1
**p**	3, 3′-Di-O-methyellagic acid-4′-β-O-d-arabinopyranoside	16.73	461.0723	461.0715	C_21_H_17_O_12_	446, 328, 313	254, 365	-	SIRIUS, the literature [18]	3
**q**	Unidentified	17.88	599.3000	599.2995	C_21_H_43_O_12_N_8_	447, 313	-	-	-	-
**r**	3, 3′-Di-O-methyl ellagic acid	19.33	329.0301	329.0292	C_16_H_9_O_8_	314, 298	254, 365	17	SIRIUS, the literature [18]	1
**s**	Kaempferol	20.52	285.0404	285.0394	C_15_H_9_O_6_	189, 171, 137	254, 365	-	GNPS, SIRIUS, the literature [25]	2
**t**	3, 3′, 4-Tri-O-methyl ellagic acid	21.64	343.0453	343.0448	C_17_H_11_O_8_	328, 313	254, 365	19	SIRIUS	1
**u**	Glycolipids	23.76	721.3655	721.3666	C_34_H_57_O_16_	415, 397, 277	-	-	GNPS, SIRIUS	4
**v**	Fatty acids	26.25	559.3124	559.3113	C_28_H_47_O_11_ *	277, 253	-	-	GNPS	4
**w**	Acetophenone-6′-xylosyl-(1-6)-glucopyranoside	13.96	535.1664	535.1658	C_22_H_31_O_15_ *	195	254	15	SIRIUS	1
**x**	Xanthoxylin-2-O-(6-galloylglucoside)	15.63	509.1298	509.1290	C_23_H_25_O_13_	313, 169	254, 365	16	SIRIUS	1
**y**	3, 4′-Di-O-methyl ellagic acid	22.28	329.0303	329.0292	C_16_H_9_O_8_	313	254, 365	18	-	1
**A**	3,4,5-Trimethoxyphenyl β-d-glucopyranoside	12.64	391.1241	391.1235	C_16_H_23_O_11_	345, 262, 174	275	1	SIRIUS	1
**B**	Scopoletin	15.94	191.0374	191.0369	C_10_H_7_O_4_	174	254, 365	4	SIRIUS	1
**C**	Juglalin	16.47	417.0825	417.0816	C_20_H_17_O_10_	284	254, 365	3	GNPS, SIRUS	1
**D**	Notoginsenoside R4	17.86	1239.6509	1239.6511	C_58_H_97_O_27_	1107, 945, 783	-	14	-	1
**E**	Ginsenoside Rb1	17.89	1107.6053	1107.6047	C_54_H_91_O_23_	945, 783	-	11	SIRIUS	1
**F**	Ginsenoside Rc	18.19	1077.5948	1077.5950	C_53_H_89_O_22_	945, 783, 621	-	12	SIRIUS	1
**G**	Ginsenoside Rb2	18.45	1077.5962	1077.5954	C_53_H_89_O_22_	945, 783, 621	-	13	SIRIUS	1
**H**	Ginsenoside Ro	18.78	955.4995	955.4994	C_48_H_75_O_19_	793	-	10	-	1
**I**	ent-Atisane-3ß, 16α, 17-triol	20.61	365.1966	365.1959	C_21_H_35_O_5_ *	-	-	5	-	1

* [M + HCOO]^−^; ** MIC: metabolite identification confidence levels (1–5). Level 1: metabolites are validated by comparing with reference standard. Level 2: putative identification of metabolites based on MS/MS fragmentation data using literature, databases and libraries. Level 3: tentative match of MS1 data through literature, databases and libraries. Level 4: completion of molecular formula identification based on adduct ion type, isotope abundance distribution, and charge state. Level 5: accurate measurement of exact mass has been established.

**Table 2:** ^1^H- and ^13^C-NMR data of novel compound 16 (CD_3_OD + D_2_O).

Position	δ_H_ (600 MHz)	δ_C_ (150 MHz)
**1**	-	114.43
**2**	-	156.01
**3**	6.34 (1H, d, 2.11)	94.44
**4**	-	162.89
**5**	6.26 (1H, d, 2.11)	92.63
**6**	-	158.40
**7**	-	196.2
**8**	2.45 (3H, s)	31.47
**1′**	4.91 (1H, d, 7.8)	101.87
**2′**	3.43 (1H, m)	73.49
**3′**	3.49 (1H, m)	76.36
**4′**	3.41 (1H, m)	70.20
**5′**	3.75 (1H, m)	74.44
**6′**	4.42 (1H, dd, 11.92, 6.57), 4.58 (1H, dd, 11.92, 2.20)	63.43
**1** **″**	-	119.93
**2** **″** **, 6** **″**	7.05 (2H, s)	108.82
**3** **″** **, 5** **″**	-	145.13

**Table 3:** DPPH and ABTS radical scavenging activity of compound **16**.

	Conc. (μM)	Radical Scavenging Activity (%)	IC_50_ (μM)
**DPPH assay**	NC	0.00 ± 0.21	
	1	4.08 ± 0.21 ***	
	10	22.89 ± 0.81 ***	
	20	34.26 ± 4.76 ***	43.7
	50	53.36 ± 2.29 ***	
	100	63.38 ± 0.62 ***	
	PC ^a^	42.99 ± 1.06 ***	
**ABTS assay**	NC	0.00 ± 0.70	
	1	4.42 ± 0.30 ***	
	5	21.13 ± 0.25 ***	
	10	37.80 ± 2.10 ***	15.8
	20	67.77 ± 2.78 ***	
	50	93.77 ± 0.14 ***	
	PC ^b^	55.54 ± 1.33 ***	

^a^ L-ascorbic acid (25 μM). ^b^ Trolox (20 μM). Note: The radical scavenging activity was calculated as the percentage compared with the NC (negative control). L-ascorbic acid and Trolox were used as positive controls. The data were expressed as mean ± S.D, *n* = 3. The significant differences between NC and treated groups were analyzed by Student’s *t*-test (*** *p* < 0.005).

## Data Availability

The original contributions presented in this study are included in the article. Further inquiries can be directed to the corresponding author.

## Supplementary Materials

The following supporting information can be downloaded at https://www.mdpi.com/article/10.3390/antiox15081023/s1 [38,39,40,41,42,56,57].

## Abbreviations

The following abbreviations are used in this manuscript:

FBMN	Feature-based molecular networking
GNPS	Global natural social molecular networking
ROS	Reactive oxygen species
NOX	NADPH oxidase
DPPH	2,2-diphenyl-1-picrylhydrazyl
ABTS	2,2′-Azino-bis(3-ethylbenzothiazoline-6-sulfonic acid) diammonium salt
CC	Column chromatography
MIC	Metabolite identification confidence level
