# Supplementary material for: Exploration of Phytochemical Constituents in Neoshirakia japonica by LC-MS and In Silico Evaluation of a New Antioxidant Targeting the NOX Family
Source: Antioxidants (Basel). 2026 Aug 17;15(8):1023. doi: 10.3390/antiox15081023 (PMC13509714; doi:10.3390/antiox15081023)
Supplement: Supplementary file 1 [file antioxidants-15-01023-s001.zip › antioxidants-4479787-supplementary.pdf]

Exploration of phytochemical constituents in *Neoshirakia japonica* by LC-MS and *in silico* evaluation of a new antioxidant targeting the NOX family

Kyeong Seon Lee [1,2], Suzy Kim [1], Le Ba Vinh [1,3], Jae Sang Han [4], Bang Yeon Hwang [4], Mi Kyeong Lee [4], and Ki Yong Lee [1,2*]

[1] College of Pharmacy, Korea University, Sejong 30019, Republic of Korea
[2] Interdisciplinary Major Program in Innovative Pharmaceutical Sciences, Korea University, Sejong 30019, Republic of Korea
[3] Department of Chemistry, University of Bergen, Bergen 5007, Norway
[4] College of Pharmacy, Chungbuk National University, Cheongju 28160, Republic of Korea
* Correspondence: kylee@korea.ac.kr; Tel.: +82-44-860-1623; Fax: +82-44-860-1606

1. Supplementary Result

1.1 Structure determination of five ginsenosides isolated from the aerial part of *N. japonica*

The molecular formula of compound **10** was assigned as $C_{48}H_{76}O_{19}$ based on its HRESIMS data, *m/z* of 955.4995 [M-H]- (calculated for $C_{48}H_{75}O_{19}$ 955.4994). In the aglycone part, total 30 carbon signals, including seven methyl groups were detected in ^{13}C- NMR. One double bond including C-12 (δ_C 123.98) and C-13 (δ_C 145.00) was observed. Correlation between C-20 (δ_C 31.69) and H-29 (δ_H 0.93)/H-30 (δ_H 0.95) was exhibited in the HMBC spectrum. This indicated compound **10** was an oleanane saponin. Three anomeric proton signals were observed and one glycosidic proton at δ_H 5.40 (d, *J* = 8.2 Hz) showed an HMBC correlation with C-28 (δ_C 178.24), indicating that β-D-glucopyranose was connected to the carboxyl group at C-28. Although no HMBC correlation was observed between H-1′ and C-3, a NOESY correlation between the anomeric proton at δ_H 4.49 (H-1′) and H-3 supported the linkage of this sugar unit at C-3. The COSY spectrum showed a correlation between H-1′ (δ_H 4.49) and H-2′ (δ_H 3.62). In the HMBC spectrum, H-2′ correlated with the anomeric carbon C-1″ (corresponding to δH 4.70, d, J = 7.8 Hz), indicating that the second sugar unit was linked to C-2′ (δ_c 76.51) of the first sugar unit. Based on the observed *m/z* 955.4995 [M-H]$^-$, the presence of one carboxylic acid in the sugar moiety was expected to match the exact mass. However, no corresponding carboxyl signal was detected. According to previous literature, sometimes the COOH signal in the glucuronopyranosyl moiety may not be detected in HMBC or 13C NMR depending on the solvent environment [56]. Therefore, it was predicted that the sugar moiety of [β-D-glucopyranosyl(1→2)-β-D-glucuronopyranosyl] was linked to the 3-OH. Accordingly, compound **10** was assigned as ginsenoside Ro. The chemical shift values of compound **10** were accompanied by the previous NMR assignment, and consistent with the reported values [42].

Compound **11**, its molecular formula was assigned as $C_{54}H_{92}O_{23}$ based on HRESIMS data, *m/z* of 1107.6053 [M-H]$^-$ (calculated for $C_{54}H_{91}O_{23}$ 1107.6047). In ^{1}H- NMR, one hydroxyl proton at δ_H 3.73 (H-12), one methine signal at δ_H 5.14 (t, J=7.6) (H-24), and eight methyl groups were observed. In ^{13}C- NMR, the aglycone part exhibited thirty carbon signals, including three hydroxyl carbons and two olefinic carbons. The aglycone structure of **11** was further confirmed by 2D NMR. Key HMBC correlations, including H-3 (δ_H 3.18)/C-28(δ_C 28.55) and C-29 (δ_C 16.84), H-12 (δ_H 3.73)/C-11(δ_C 30.97) andC-13 (δ_C 49.85), H-13 (δ_H 1.74)/C-30 (δ_C 16.88), and C-20 (δ_C 85.12)/H-17 (δ_H 2.29), H-21 (δ_H 1.36), H-22 (δ_H 1.52/1.79) confirmed its aglycone moiety as a dammarane-type skeleton. This aglycone moiety was consistent across compounds **11–14**. Four β-D glucopyranose were identified in compound **11**. The configurations of sugar moieties were determined by J-values of their anomeric protons, which ranged from 7.7 to 7.8 Hz. The sugar linkages were identified by HMBC and COSY. H-1' (δ_H 4.43, d, *J* = 7.7 Hz) was correlated with C-3 (δ_C 3.18), and H-1''' (δ_H 4.58, d, *J* = 7.8 Hz) was correlated to C-20 (δ_C 85.12). Correlation peaks between H-1'' (δ_H 4.67, d, *J* = 7.7 Hz)/C-2' (δ_C 81.21) and H-1'''' (δ_H 4.35, d, *J* = 7.8 Hz)/C-6''' (δ_C 70.39) were also exhibited in the HMBC spectrum. The chemical shift values of compound **11** were consistent with the previous literature, so it was determined as ginsenoside Rb1 [38–40]

Compound **12**, the molecular formula was assigned as $C_{53}H_{90}O_{22}$ based on its HRESIMS data, *m/z* of 1077.5948 [M-H]$^-$ (calculated for $C_{53}H_{89}O_{22}$ 1077.5950). In the HMBC spectrum, the linkages between sugar moieties and the aglycone were found to be the same as those of compound **11**, but the *J*-value of the anomeric proton signal at δ_H 4.92 (H-1'''') was 1.4 Hz, which means the existence of L-arabinofuranose. This proton signal showed an HMBC correlation with C-5''' (δ_C 63.27). The sugar linkages were confirmed by COSY correlations. By carefully comparing the chemical shift values with the previous reports, the structure of compound **12** was assigned as ginsenoside Rc [38,40].

Compound **13**, the molecular formula was assigned as $C_{53}H_{90}O_{22}$ based on its HRESIMS data, *m/z* of 1077.5962 [M-H]$^-$ (calculated for $C_{53}H_{89}O_{22}$ 1077.5954). Analysis of the 1D and 2D NMR data revealed

the presence of four sugar residues. The sugar moiety associated with the anomeric proton H-1'''' (δ_H 4.34, d, J = 6.2 Hz) was identified as L-arabinopyranose based on its coupling constant and 2D NMR correlations. Consequently, compound **13** was identified as ginsenoside Rb2. The chemical shift values of compound **13** were accompanied by the previous literature which reported the NMR assignment of ginsenoside Rb2 [39,41]

Compound **14**, its molecular formula was determined as $C_{59}H_{100}O_{17}$ based on its HRESIMS data, *m/z* of 1239.6509 $[M-H]^-$ (calculated for $C_{59}H_{99}O_{17}$ 1239.6511). The aglycone moiety was confirmed as dammarane-type triterpenoid by comparing its NMR signals with reported assignments and those of other three ginsenosides (**11**-**13**). In the sugar region of the 1D NMR spectra, five anomeric proton signals were observed. All the sugar linkages were confirmed through HMBC and COSY analysis. The sugar moiety, [β-D-glucopyranosyl(1→2)-β-D-glucuronopyranosyl], was linked to the hydroxyl group of C-3 (δ_H 3.25, δ_C 88.75), while the sugar moiety of [β-D-xylopyranosyl (1→6)-β-D-glucuronopyranosyl(1→6)-β-D-glucuronopyranosyl] was linked to the hydroxyl group of C-20 (δ_C 83.23). The correlation peaks between H-1′ (δ_H 4.89, d, J = 7.7 Hz)/C-3 (δ_C 88.75), H-1″(δ_H 5.35, d, J = 7.7 Hz)/C-2′ (δ_C 83.23), H-1‴ (δ_H 5.10, d, J = 7.8 Hz)/C-20 (δ_C 83.23), H-1⁗ (δ_H 5.00, d, J = 7.8 Hz)/ C-6‴ (δ_C 70.10), and H-1‴″(δ_H 4.93, d, J = 7.4 Hz)/ C-6⁗ (δ_C 69.66) were observed in HMBC spectrum. In addition, the fifth sugar unit was identified as β-D-xylopyranose based on its correlations with five proton and carbon signals. These substitution patterns identified compound **14** as notoginsenoside R4. The chemical shift values of compound **14** were accompanied by the previous literature and structure was determined to be notoginsenoside R4 [41,57].

Table S1. Parameter screening for FBMN

	Cosign score	Fragment matched ion numbers	Total numbers of nodes/edges	Numbers of nodes/edges in tannin clusters	Numbers of nodes/edges in saponin clusters	GNPS library hits
NJAP and NJR extract	0.8	≥6	5353/5572	nd	14/19, 14/20	1
	0.7	≥6	5358/5818	48/112	30/69	4
	0.7	≥4	5353/5972	23/35	32/70	86
	0.6	≥6	5353/5951	50/119	43/107	4
NJAP solvent fractions	0.7	≥6	4433/4671	49/135	26/52	67
	0.7	≥4	4433/4770	50/119	29/58	18

Table S2. Extraction yields of the extracts and solvent fractions

	Amount	Extraction yield (%)[a]
**Dried aerial part of *N. japonica***	3 kg	-
**Aerial part (AP) extract**	186.2 g	6.21 %
***n*-Hexane fraction of AP extract**	7.7 g	0.25 %

**EtOAC fraction of AP extract**	27.4 g	0.91 %
***n*-BuOH fraction of AP extract**	35.4 g	1.2 %
**Water fraction of AP extract**	96.8 g	3.2 %
**Dried root part of *N. japonica***	160 g	-
**Root extract**	9.13 g	5.71 %

[a] Extraction rate = (amount of extract or each solvent phase / amount of dried plant material) *100

Table S3. ^{1}H- NMR of five isolated saponins (600 MHz).

Position	10[a,b]	11[c,b]	12[c,b]	13[c,b]	14[a,d]
**1**	0.99, 1.61	1.04, 1.73	1.04, 1.71	1.03, 1.72	0.70, 1.52
**2**	1.71, 1.99	1.74	1.75	1.74	1.80, 2.19
**3**	3.21	3.18	3.19	3.20	3.25 (1H, dd, 11.7, 4.2)
**4**	-	-	-	-	-
**5**	0.78 (1H, d, 11.8)	0.78 (1H, d, 11.5)	0.78 (1H, d, 11.6)	0.78 (1H, d, 10.7)	0.63 (1H, d, 11.2)
**6**	1.40, 1.55	1.50	1.48	1.49	1.33, 1.42
**7**	1.32, 1.49	1.29	1.28	1.25	1.16, 1.42
**8**	-	-	-	-	-
**9**	1.57	1.44	1.44	1.44	1.31
**10**	-	-	-	-	-
**11**	1.89	1.23, 1.79	1.25, 1.80	1.24, 1.77	1.54, 1.93
**12**	5.27 (1H, t, 3.8)	3.73	3.72	3.71	4.17
**13**	-	1.74	1.74	1.74	1.97
**14**	-	-	-	-	-
**15**	1.81	1.04, 1.57	1.04, 1.56	1.04, 1.56	0.94, 1.49
**16**	1.72, 2.05	1.31, 1.89	1.29, 1.91	1.29, 1.88	1.31, 1.80
**17**	-	2.29	2.28	2.29	2.57
**18**	2.87	1.01 (3H, s)	1.01 (3H, s)	1.00 (3H, s)	0.93 (3H, s)
**19**	1.14, 1.72	0.92 (3H, s)	0.92 (3H, s)	0.92 (3H, s)	0.79 (3H, s)

**20**	-	-	-	-	-
**21**	1.22, 1.40	1.36 (3H, s)	1.36 (3H, s)	1.36 (3H, s)	1.64 (3H, s)
**22**	1.62, 1.73	1.52, 1.79	1.57, 1.77	1.56, 1.79	1.79, 2.37
**23**	1.09 (3H, s)	2.04, 2.13	2.04, 2.13	1.99, 2.13	2.35, 2.57
**24**	0.87 (3H, s)	5.14 (1H, t, 7.6)	5.14 (1H, 7.3)	5.13 (1H, t, 7.7)	5.28 (1H, t, 7.6)
**25**	0.96 (3H, s)	-	-	-	-
**26**	0.81 (3H, s)	1.69 (3H, s)	1.70 (3H, s)	1.69 (3H, s)	1.58 (3H, s)
**27**	1.17 (3H, s)	1.63 (3H, s)	1.63 (3H, s)	1.63 (3H, s)	1.62 (3H, s)
**28**	-	1.07 (3H, s)	1.07 (3H, s)	1.07 (3H, s)	1.26 (3H, s)
**29**	0.93 (3H, s)	0.86 (3H, s)	0.86 (3H, s)	0.86 (3H, s)	1.09 (3H, s)
**30**	0.95 (3H, s)	0.92 (3H, s)	0.92 (3H, s)	0.92 (3H, s)	0.95 (3H, s)
**3-O-glc-1'**	4.49 (1H)	4.43 (1H, d, 7.7)	4.43 (1H, d, 7.3)	4.43 (1H, d, 7.3)	4.89 (1H, d, 7.7)
**2'**	3.62	3.56	3.57	3.54	4.23
**3'**	3.21	3.27	3.39	3.20-3.36	4.31
**4'**	3.23-3.30	3.20-3.28	3.19-3.29	3.54	4.12
**5'**	3.63	3.20-3.36	3.27	3.18-3.28	3.91
**6'**	-	3.59-3.67, 3.78-3.87	3.64, 3.85	3.64, 3.84	4.32, 4.54
**2'-O-glc-1''**	4.70 (1H, d, 7.8)	4.67 (1H, d, 7.7)	4.68 (1H, d, 7.7)	4.67 (1H, d, 7.7)	5.35 (1H, d, 7.7)
**2''**	3.23	3.21	3.20	3.23	4.11
**3''**	3.36	3.33-3.36	3.35	3.20-3.36	4.23
**4''**	3.24	3.20-3.28	3.19-3.29	3.20-3.28	4.2-4.31
**5''**	3.25	3.20-3.36	3.20	3.18-3.28	4.30
**6''**	3.63, 3.84	3.59-3.67, 3.78-3.87	3.64, 3.85	3.64, 3.84	4.46
**20-O-glc-1'''**	-	4.58 (1H, d, 7.8)	4.58 (1H, d, 7.8)	4.59 (1H, d, 7.8)	5.10 (1H, d, 7.8)
**2'''**	-	3.12	3.10 (1H, dd, 7.8, 1.3)	3.11	3.88
**3'''**	-	3.32-3.36	3.34	3.20-3.36	4.15
**4'''**	-	3.20-3.28	3.19-3.29	3.20-3.28	4.03
**5'''**	-	3.43	3.27	3.40	4.00
**6'''**	-<br>-	3.79, 4.06	3.55, 3.98	3.80, 4.01	4.30, 4.74
**6'''-O-sugar-1''''**	-	4.35 (1H, d, 7.8)	4.92 (1H, d, 1.4)	4.34 (1H, d, 6.2)	5.00 (1H, d, 7.8)
**2''''**	-	3.21	3.97	3.56	3.96
**3''''**	-	3.33-3.36	3.54	3.20-3.36	4.11
**4''''**	-	3.20-3.28	3.97	3.20-3.28	4.15
**5''''**	-	3.20-3.36	3.71, 3.82	3.52, 3.80	4.01
**6''''**	-<br>-	3.63, 3.84	-	-	4.26, 4.78
**1'''''**	-	-	-	-	4.93 (1H, d, 7.4)
**2'''''**	-	-	-	-	3.99
**3'''''**	-	-	-	-	4.12
**4'''''**	-	-	-	-	4.18

**5'''''**	-	-	-	-	3.64, 4.30
**28-O-glc-1**	5.40 (1H, d, 8.2)		-	-	-
**-2**	3.33		-	-	-
**-3**	3.41		-	-	-
**-4**	3.36		-	-	-
**-5**	3.36		-	-	-
**-6**	3.69, 3.82		-	-	-

[a] Recorded with 800 MHz NMR

[b] dissolved in Methanol-d_4

[c] recorded with 600 MHz NMR

[d] dissolved in Pyr-d_5

Table S4. ^{13}C- NMR of four isolated saponins (150 MHz).

**Position**	**10[a,d]**	**11[c,d]**	**12[c,d]**	**13[c,d]**	**14[b,c]**
**1**	40.04	40.40	40.39	40.39	38.97
**2**	27.13	27.39	27.40	27.40	26.54
**3**	91.54	91.50	91.49	91.50	88.75
**4**	40.57	40.73	40.74	40.74	39.46
**5**	57.18	57.69	57.70	57.69	56.15
**6**	19.48	19.39	19.40	19.39	18.19
**7**	34.11	35.99	36.00	36.00	34.90
**8**	40.87	41.13	41.13	41.13	39.80
**9**	49.65	51.24	51.24	51.24	49.99
**10**	38.02	38.07	38.07	38.06	36.66
**11**	24.73	30.97	30.98	36.00	30.49
**12**	123.98	71.82	72.08	72.34	69.90
**13**	145.00	49.85	49.99	49.87	49.28
**14**	43.08	52.55	52.57	52.56	51.29
**15**	29.05	31.64	31.65	31.63	30.60
**16**	24.15	27.39	27.40	27.40	26.40
**17**	48.18	53.06	53.07	49.87	51.16
**18**	42.76	16.88	17.49	17.51	15.78
**19**	47.37	16.46	16.46	16.46	16.04
**20**	31.69	85.12	85.07	85.17	83.23
**21**	35.05	22.63	22.58	22.53	22.13

**22**	33.30	36.93	36.97	36.99	35.99
**23**	28.62	24.04	24.00	24.03	22.93
**24**	17.09	126.18	126.20	126.11	125.77
**25**	16.21	132.40	132.28	132.49	130.80
**26**	17.89	26.09	26.06	26.06	25.58
**27**	26.46	18.17	18.12	18.11	17.74
**28**	178.24	28.55	28.54	28.53	27.86
**29**	33.65	16.84	16.84	16.84	16.37
**30**	24.12	16.88	16.88	16.88	17.19
**3-O-Glu-1'**	105.61	105.55	105.55	105.56	104.89
**2'**	81.42	81.21	81.25	81.21	83.23
**3'**	76.51	78.04	78.67	76.71	78.06
**4'**	73.74	71.67	71.88	71.71	71.43
**5'**	78.40	78.09	77.86	78.67	78.06
**6'**	Nd	63.27	63.00	62.99	62. 64
**2'-O-glc-1''**	104.81	104.64	104.67	104.65	105.84
**2''**	76.51	76.50	76.48	74.13	76.93
**3''**	77.98	78.49	78.50	78.50	77.74
**4''**	72.01	71.74	72.13	71.75	71.43
**5''**	78.40	78.09	78.04	77.86	78.13
**6''**	63.19	62.98	63.14	63.27	62.46
**20-O-glc-1'''**	-	98.25	98.21	98.29	97.83
**2'''**	-	75.43	75.50	75.43	74.63
**3'''**	-	78.66	78.74	78.67	79.08
**4'''**	-	71.85	71.76	71.84	71.25
**5'''**	-	76.95	76.58	76.47	76.71
**6'''**	-	70.39	68.40	69.11	70.10
**6'''-O-sugar-1''''**	-	105.14	110.05	98.29	105.25
**2''''**	-	75.29	85.92	72.08	74.84
**3''''**	-	77.86	79.09	78.04	77.91
**4''''**	-	72.08	83.38	69.36	71.31
**5''''**	-	78.69	63.27	66.07	76.76
**6''''**	-	62.97	-	-	69.66
**6''''-O-sugar-1'''''**	-	-	-	-	105.74
**2'''''**	-	-	-	-	74.63
**3'''''**	-	-	-	-	77.89
**4'''''**	-	-	-	-	70.90
**5'''''**	-	-	-	-	66.88
**28-O-glc-1**	95.87			-	
**-2**	74.08			-	
**-3**	78.46			-	
**-4**	71.26			-	

**-5**	78.82	-
**-6**	62.56	-

[a] Recorded with 200 MHz NMR

[b] dissolved in Methanol-d_4

[c] recorded with 150 MHz NMR

[d] dissolved in Pyr-d_5

Table S5. Docking score and glide energy scores of compound **16** with NOX2.

Compound	XP dock score (Kcal/mol)	XP_Hbond (Kcal/mol)	Glide energy (Kcal/mol)	Glide evdw (Kcal/mol)	Glide ecoul (Kcal/mol)	Glide emodel (Kcal/mol)	H-bond
**PC[a]**	-10.691	-1.023	-48.435	-33.738	-14.697	-52.280	ARG56, ARG67
**Compound 16**	-7.083	-3.878	-42.115	-30.436	-11.679	-51.771	ARG45, ARG67, GLU66, THR194, LYS195
**L-ascorbic acid**	-5.145	-1.678	-20.267	-14.786	-5.481	-20.431	ALA109

NOTE : [a]Co-crystallized ligand of PDB 7U8G : (2S)-3-(hexadecanoyloxy)-2-[(9Z)-octadec-9-enoyloxy]propyl 2-(trimethylammonio)ethyl phosphate

Table S6. Docking score and glide energy scores of compound **16** with NOX5.

Compound	XP dock score (Kcal/mol)	XP_Hbond (Kcal/mol)	Glide energy (Kcal/mol)	Glide evdw (Kcal/mol)	Glide ecoul (Kcal/mol)	Glide emodel (Kcal/mol)	H-bond	Pi-Pi stacking
**PC**[a]	-17.579	-7.625	-106.318	-68.196	-38.122	-205.914	THR445, THR462, THR484 THR541, PRO460, HIE459, HIE 476, ILE477, SER482, TRP695	TRP695
**Compound 16**	-11.543	-4.197	-59.696	-40.746	-18.950	-92.287	THR462, GLU691, ASN692	-
**L-ascorbic acid**	-7.650	-3.930	-27.881	-13.768	-14.113	-35.535	PRO460, THR462, HIE476, ILE477, ARG478	-

NOTE : [a]Co-crystallized ligand of PDB 5O0X: flavin-adenine dinucleotide

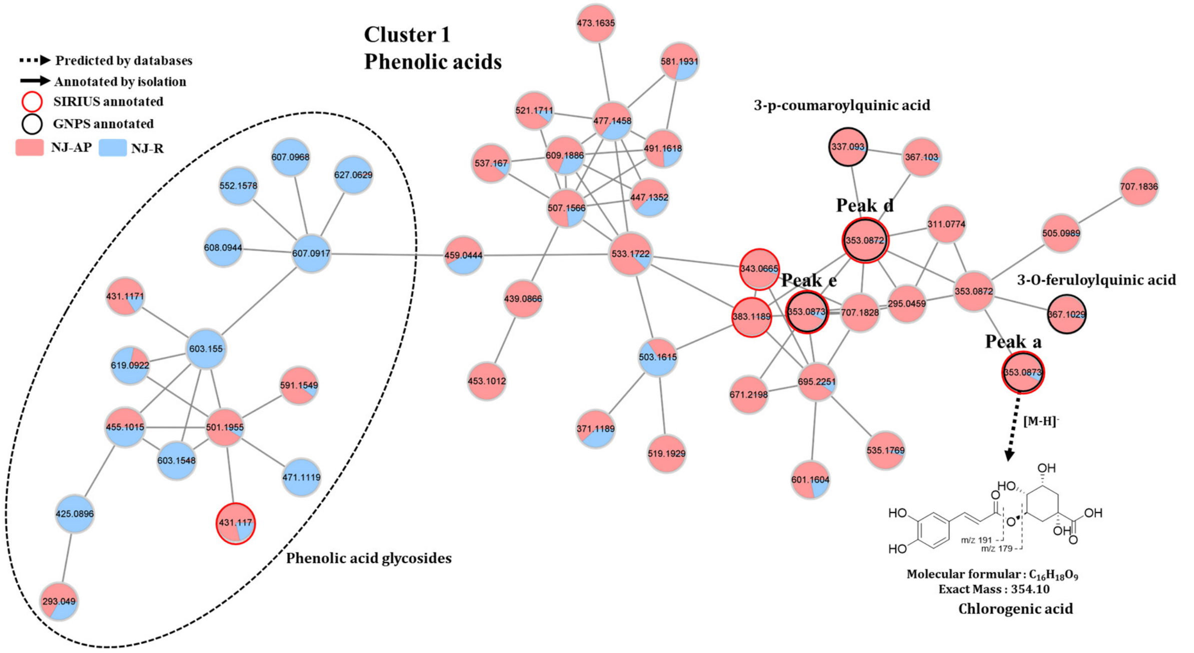

Figure S1. Putative prediction of phenolic acids based on ms/ms fragmentation in cluster 1, and the peak **a**, **d**, and **e** which were detected in chemical profiles were marked.

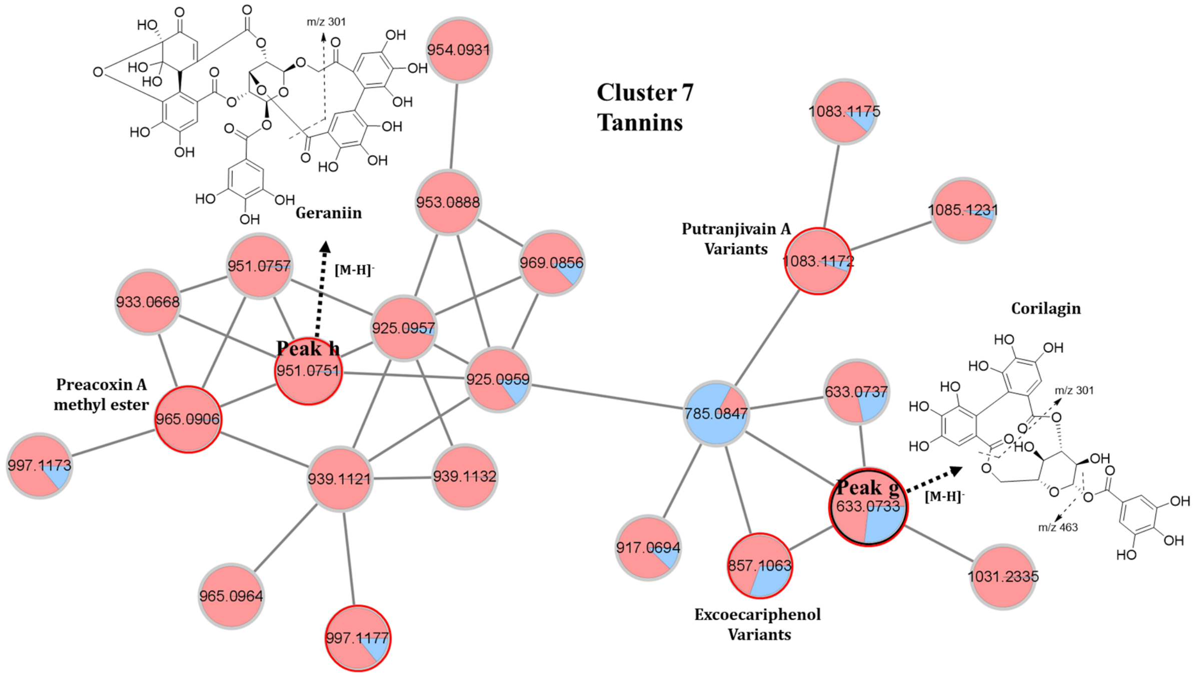

Figure S2. Tentative molecular prediction of ellagitannins in cluster 7. The peak **g** and **h** werdesignated with black dashed arrows.

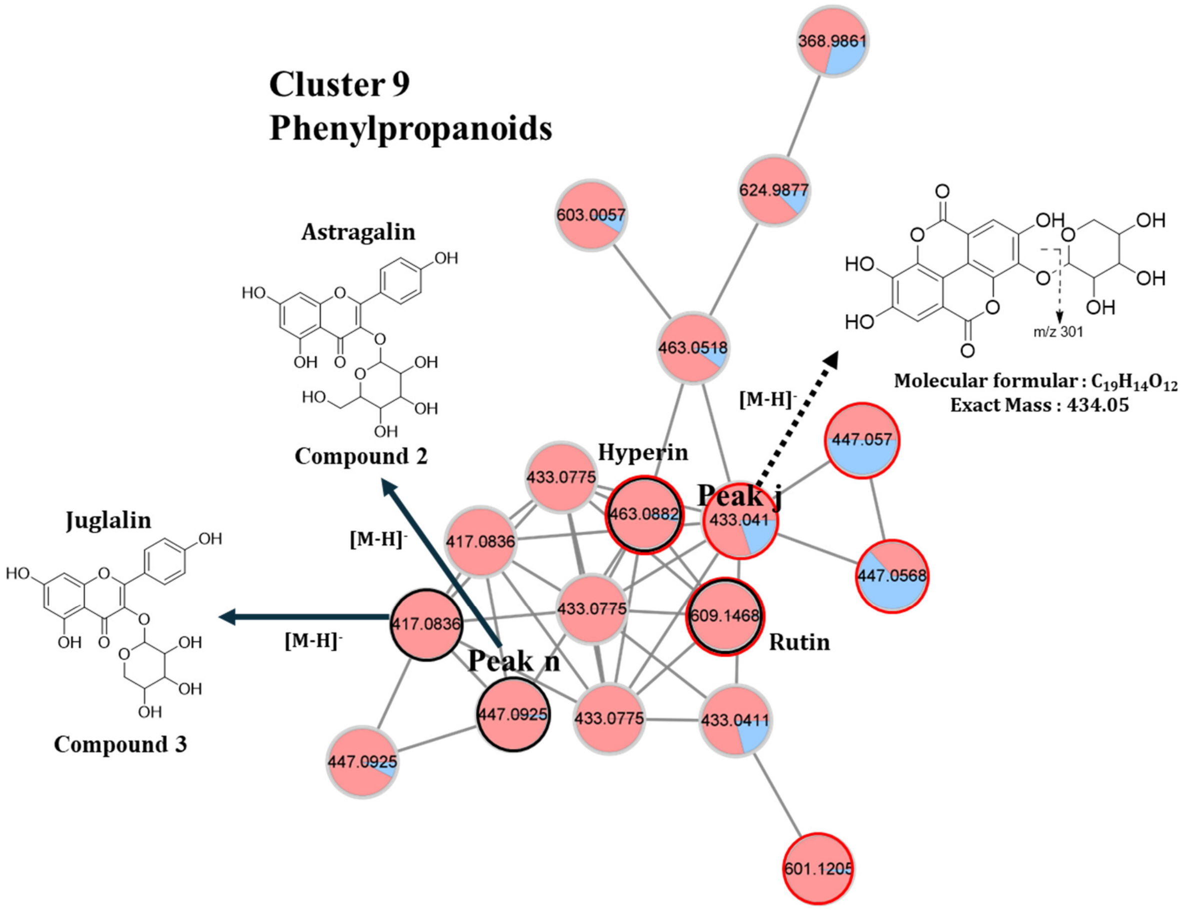

Figure S3. Putative prediction of flavonoids and ellagic acids based on ms/ms fragmentation in cluster 9. The peak **n** and **j** were indicated and isolated compounds were displayed with black arrows.

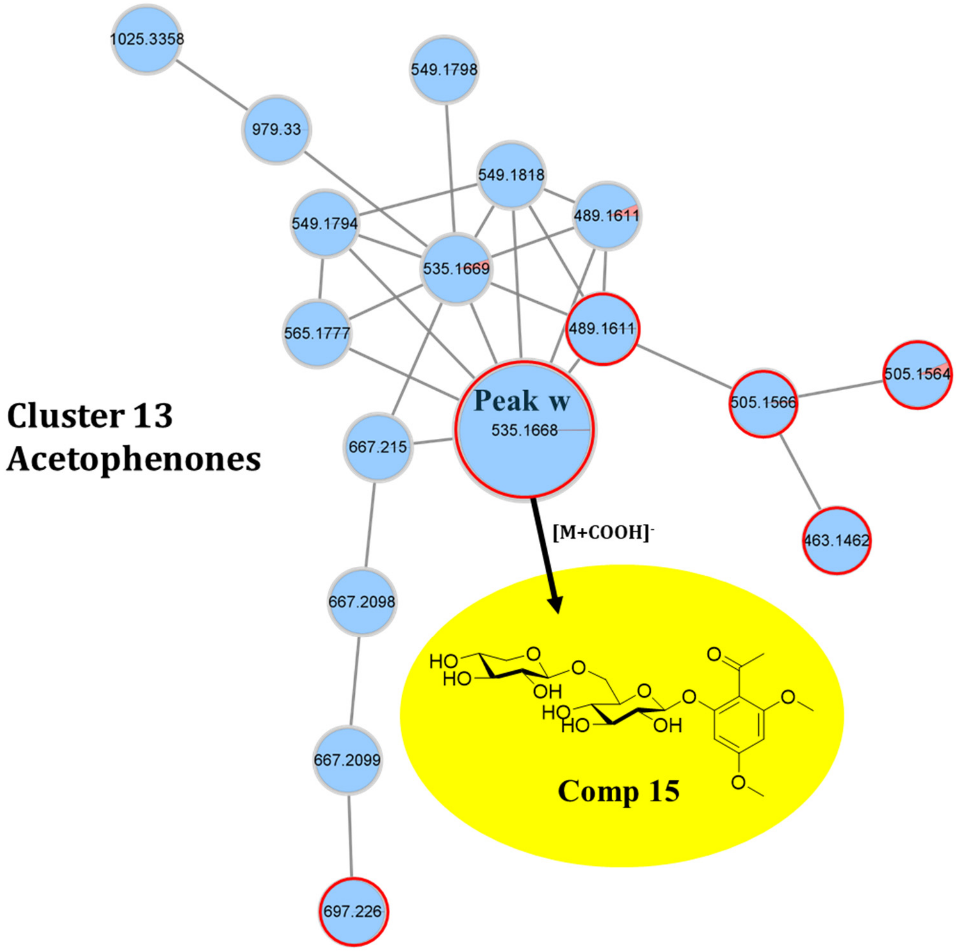

Figure S4. The peak **w** was designated with black arrow in cluster 13, and the molecular structure displayed.

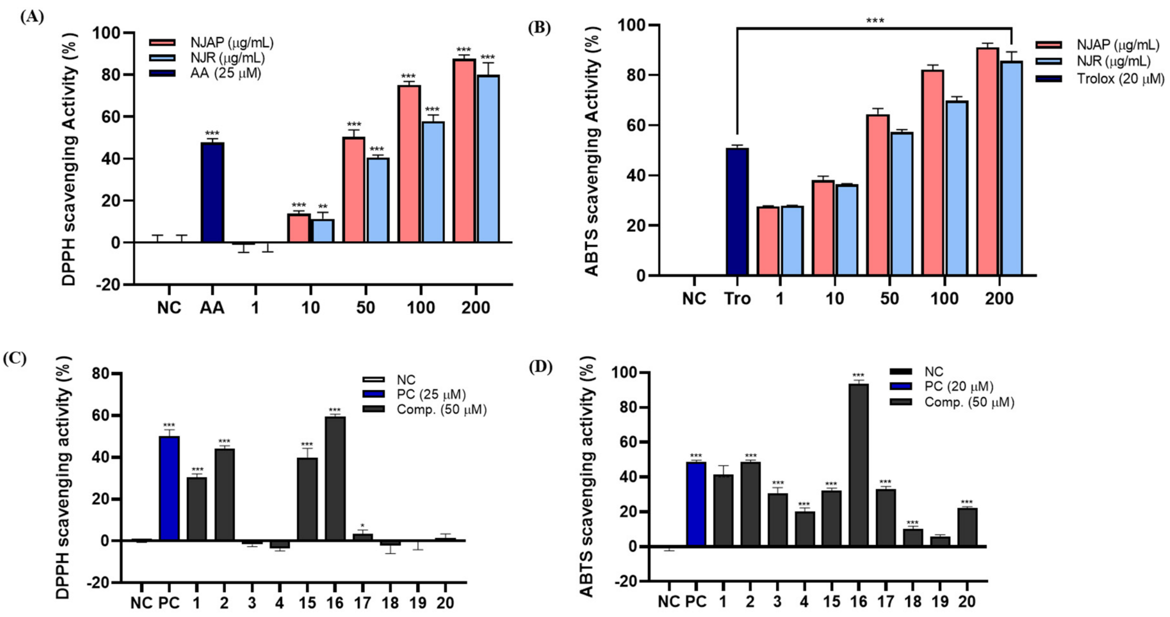

Figure S5. Radical scavenging activities of *N. japonica* extracts and phenolic compounds; (A)(B) The radical scavenging activities of *N. japonica* extracts; (C)(D) antioxidant activity screening results of isolated phenolic compounds **1-4** and **15-20**.

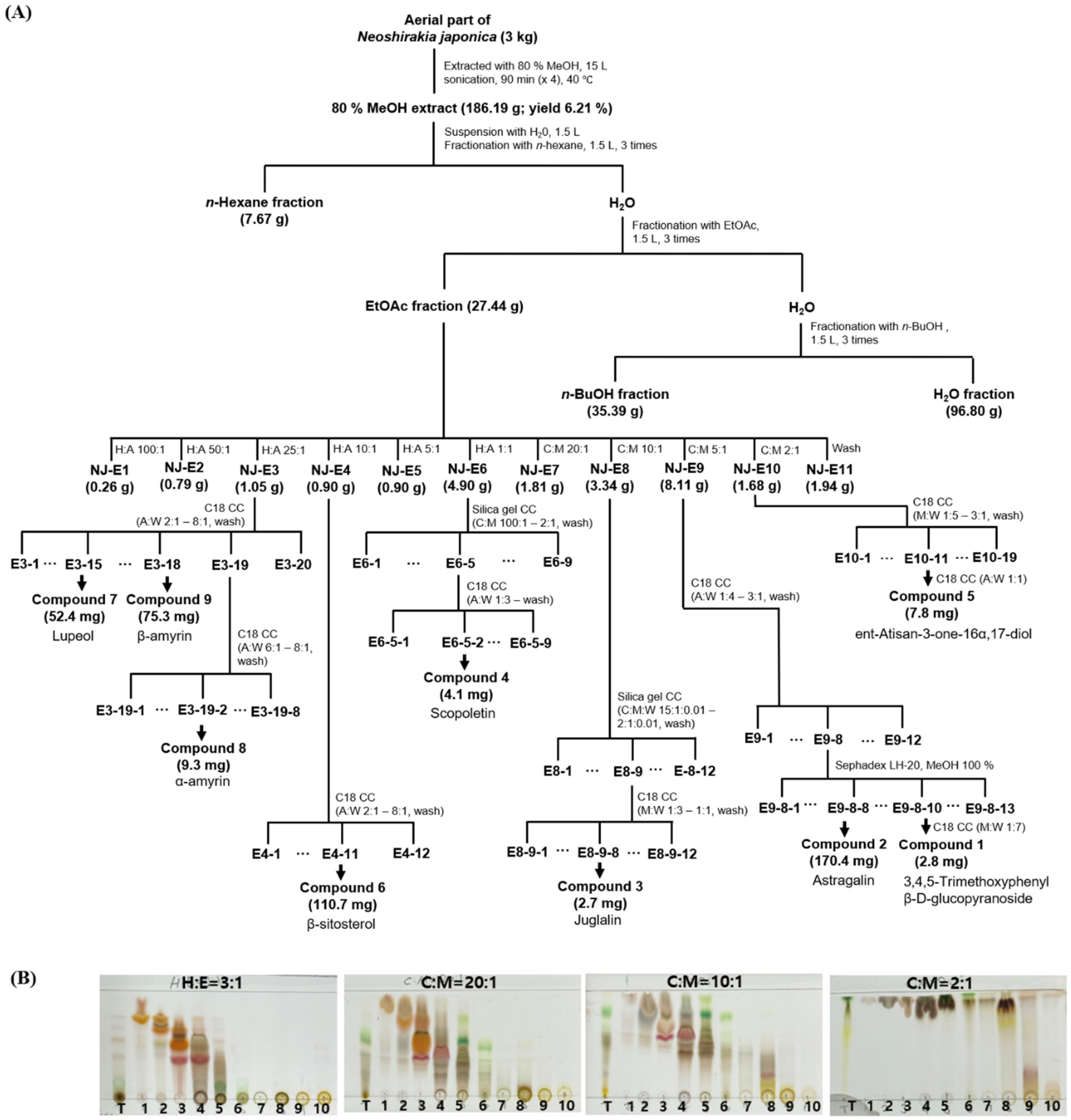

Figure S6. Isolation scheme of *N. japonica* aerial part; (A) a diagram of isolation process; (B) NP TLC analysis of the EtOAC subfraction (NJ-E1 to NJ-E10).

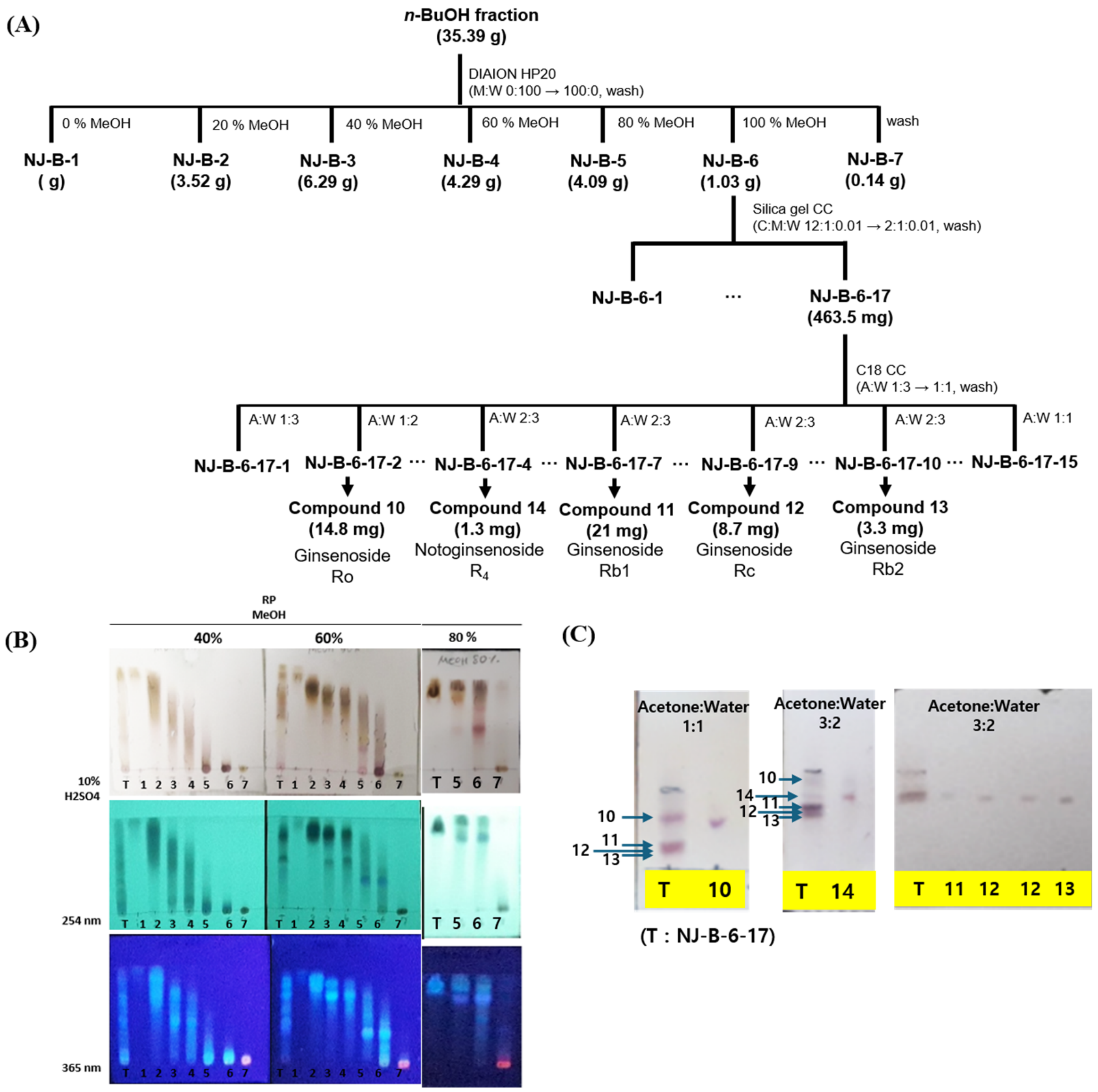

Figure S7. Isolation scheme of n-BuOH fraction from aerial part of *N. japonica*; (A) a diagram of isolation process; (B) NP TLC analysis of seven subfractions, NJ-B-1 to NJ-B-7; (C) RP TLC showing the distribution of compound **10-14** in NJ-B-6-17 fraction.

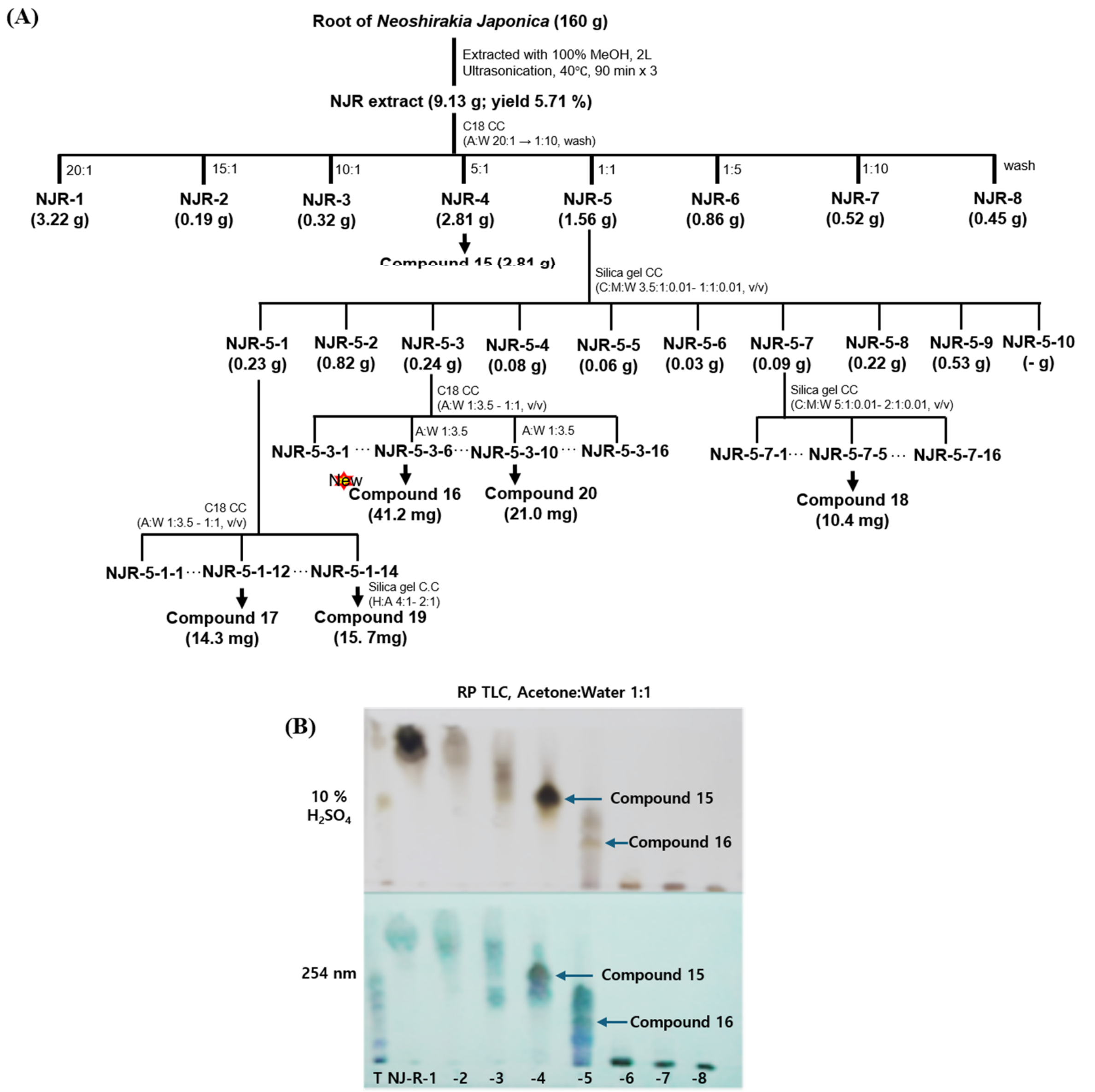

Figure S8. Isolation scheme of *N. japonica* root extract; (A) a diagram of isolation process; (B) TLC analysis of eight subfractions showing the distribution of acetophenone derivatives.

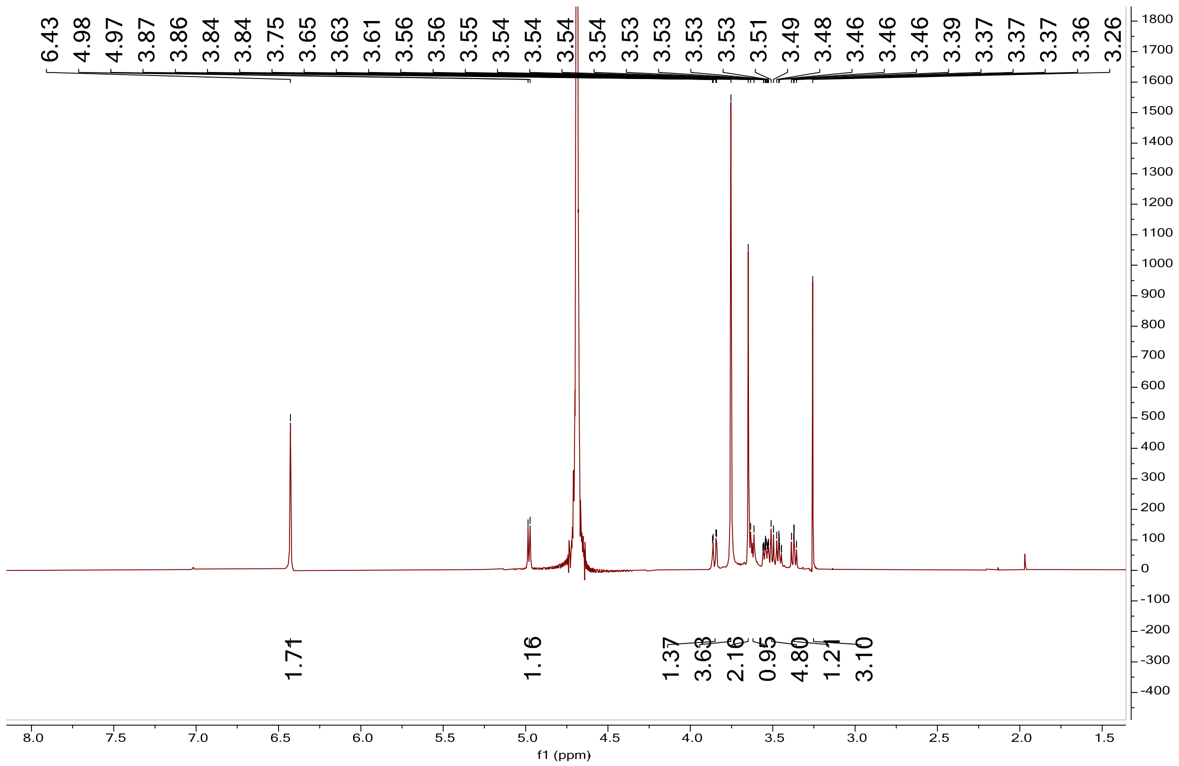

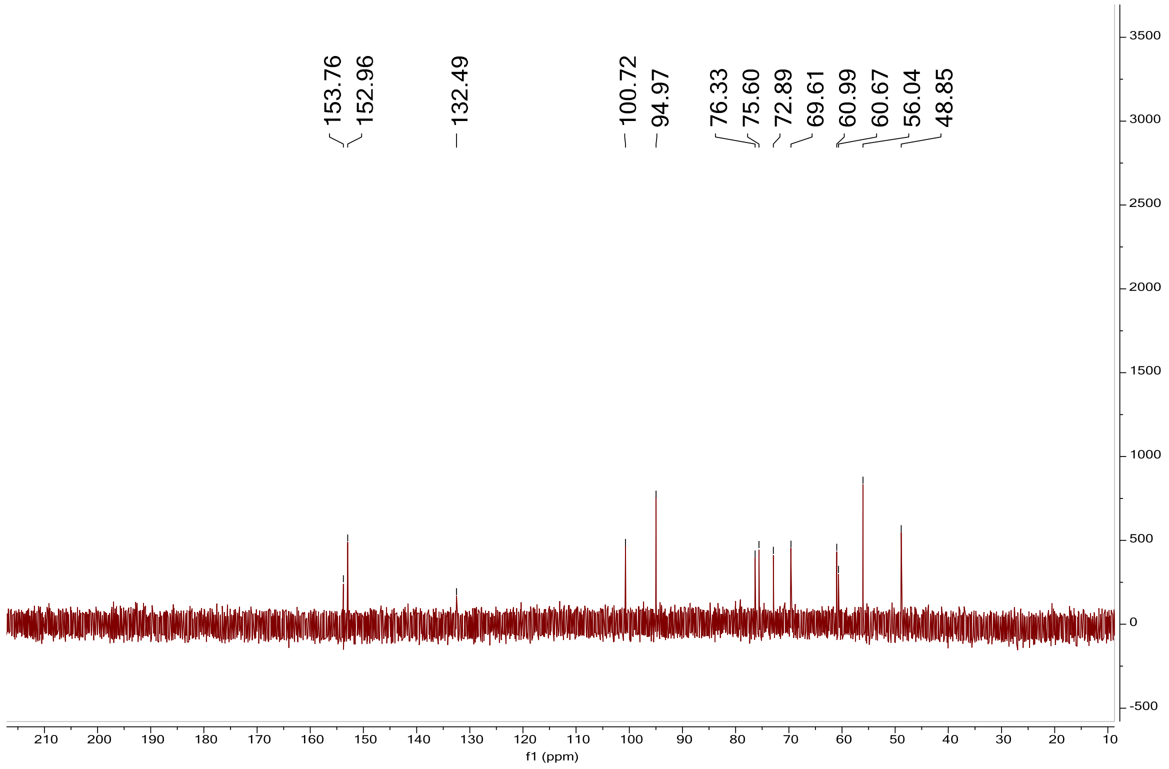

Figure S9. ^{1}H- and ^{13}C-NMR spectra of compound **1** (600 and 150 MHz, D_2O).

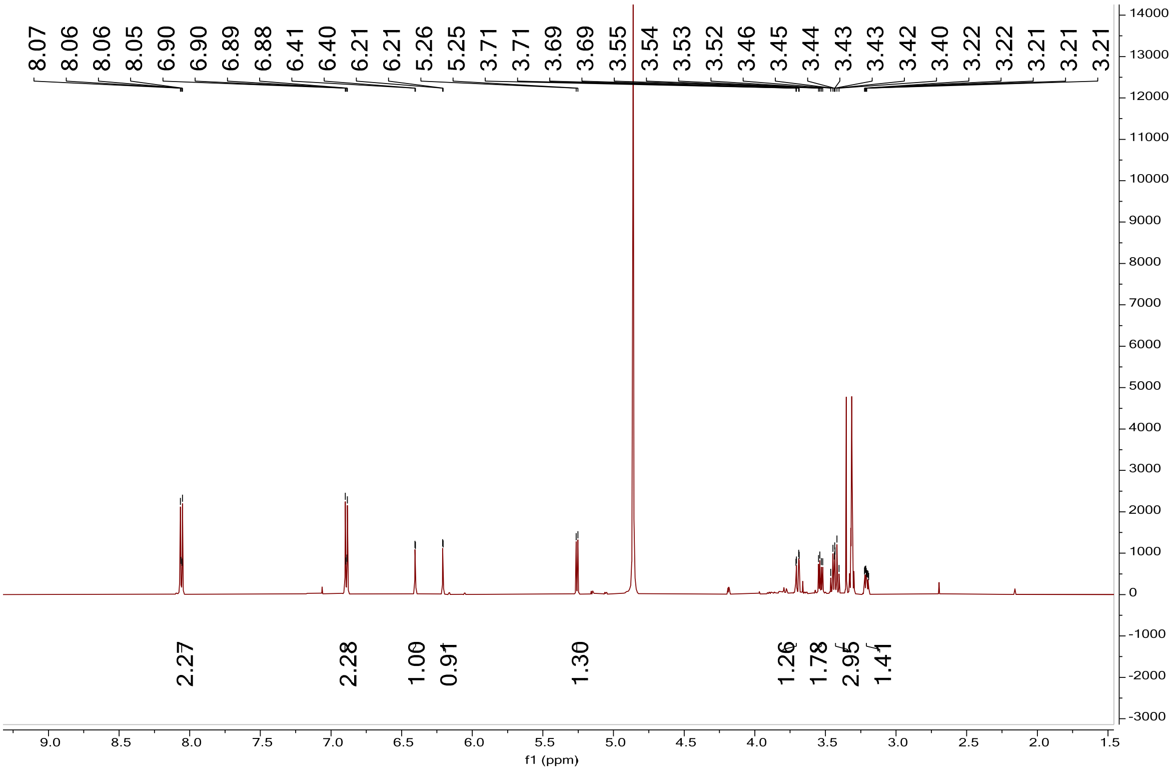

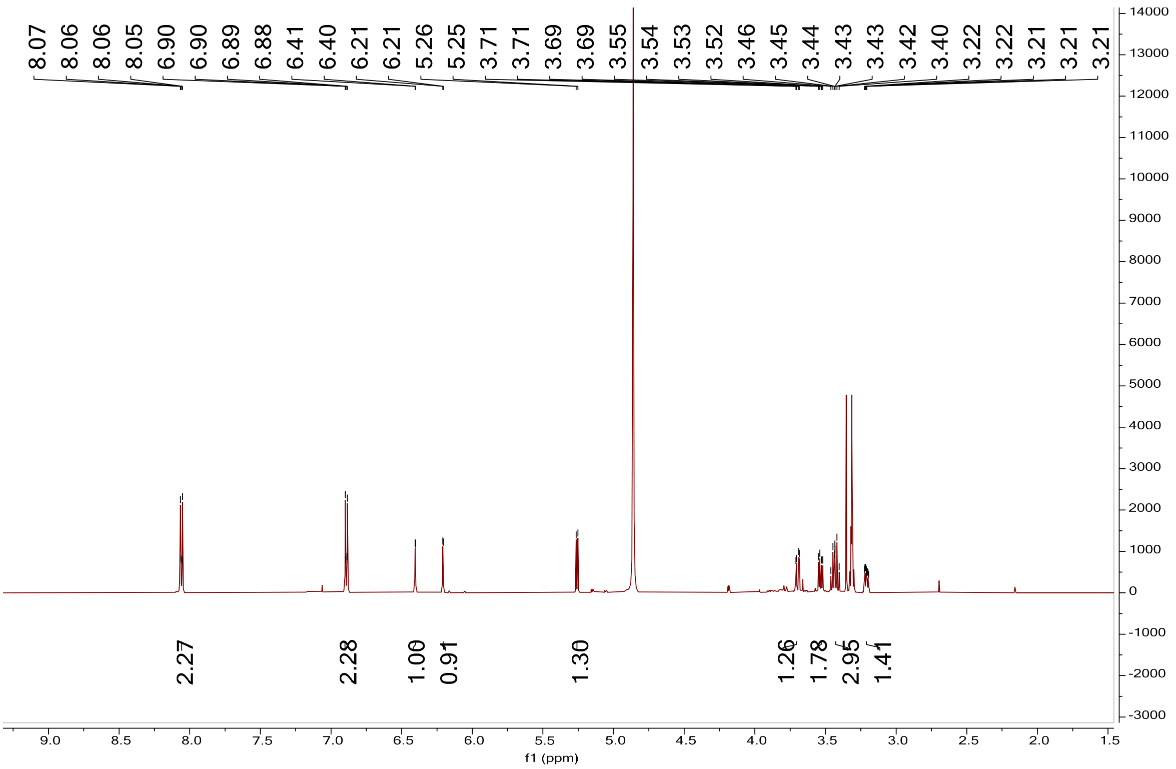

Figure S10. ^{1}H- and ^{13}C-NMR of compound **2** (600 and 150 MHz, CD_3OD).

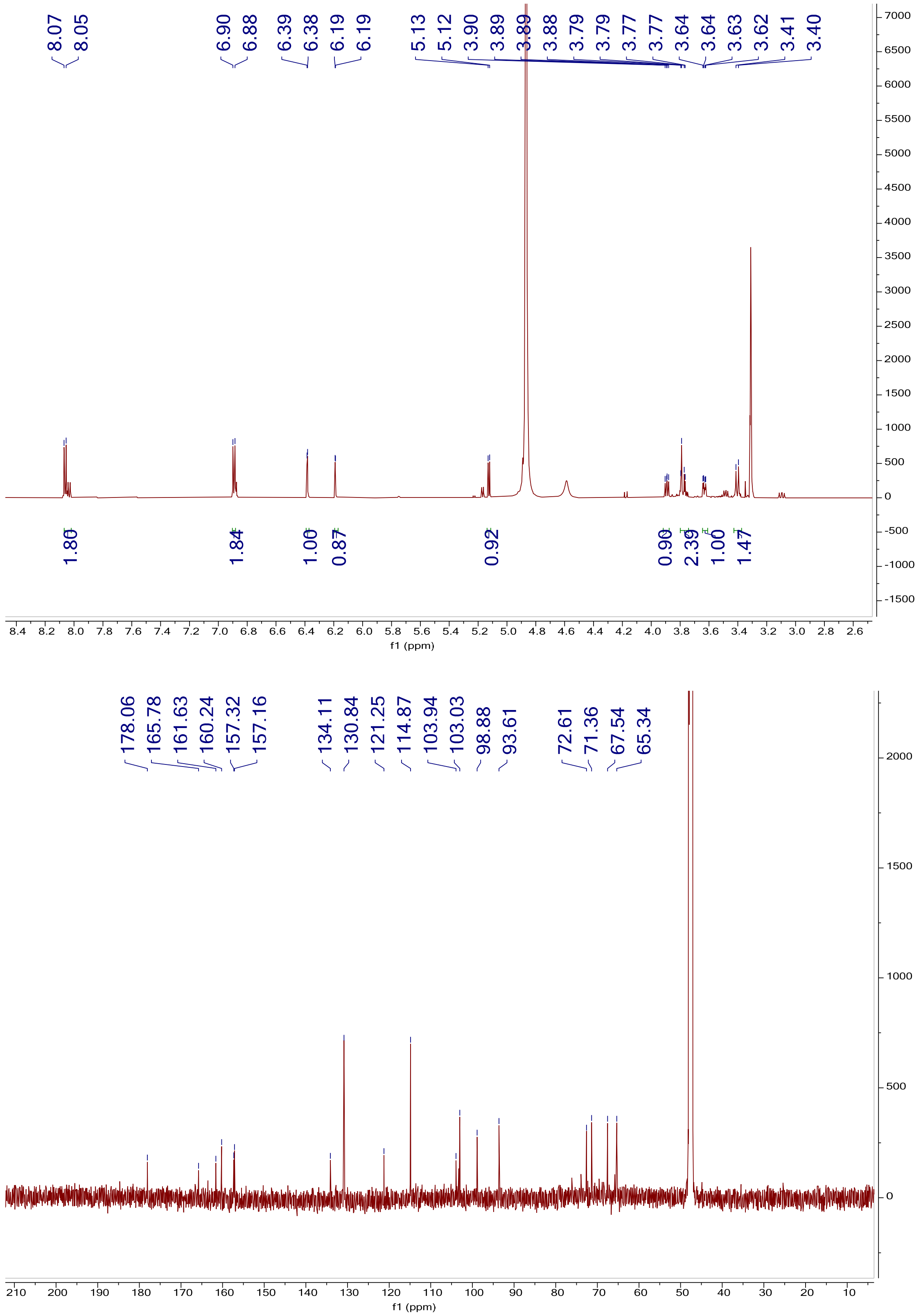

Figure S11. ^{1}H- and ^{13}C-NMR spectra of compound **3** (600 and 150 MHz, CD_3OD).

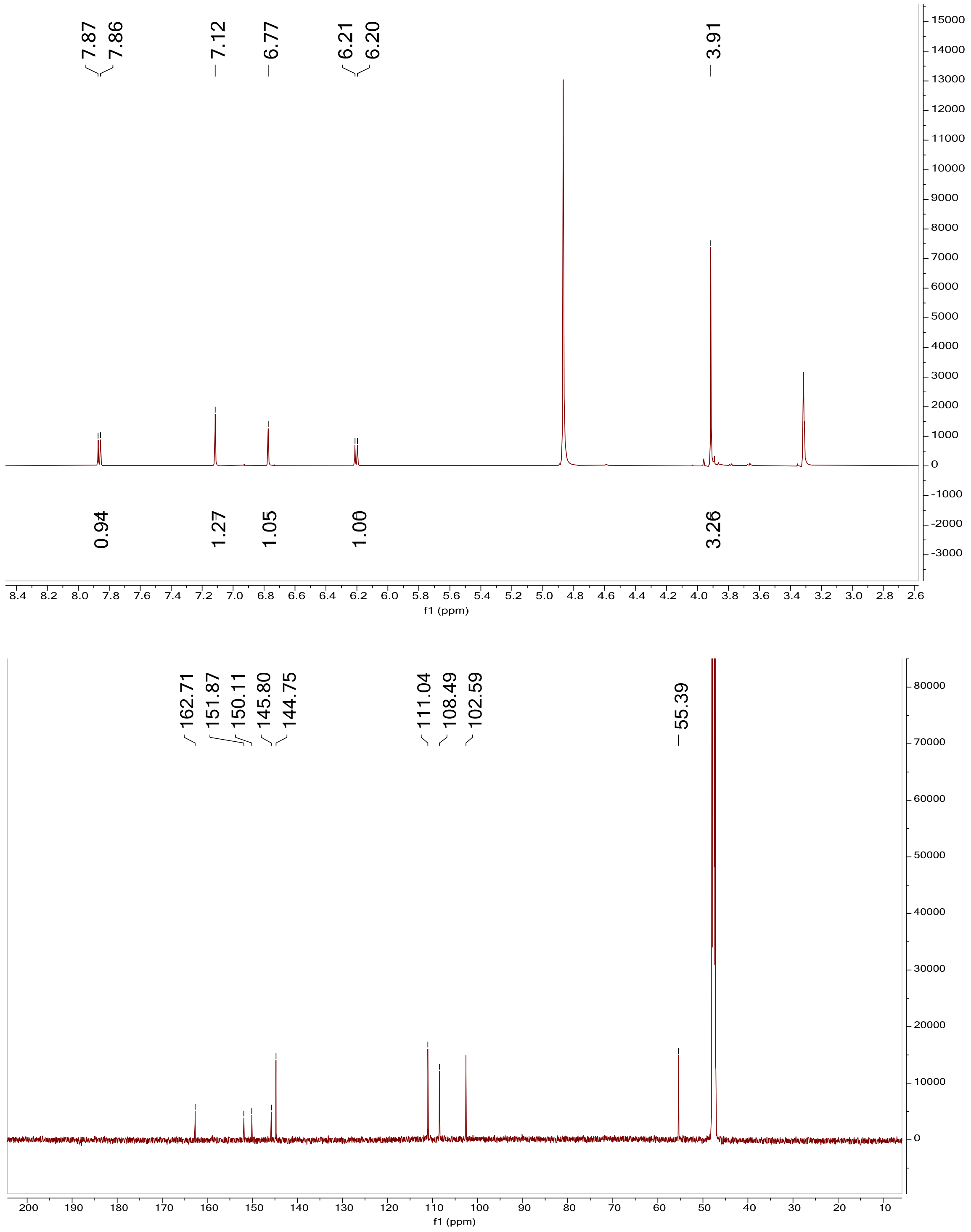

Figure S12. ^{1}H- and ^{13}C-NMR spectra of compound **4** (600 and 150 MHz, CD_3OD).

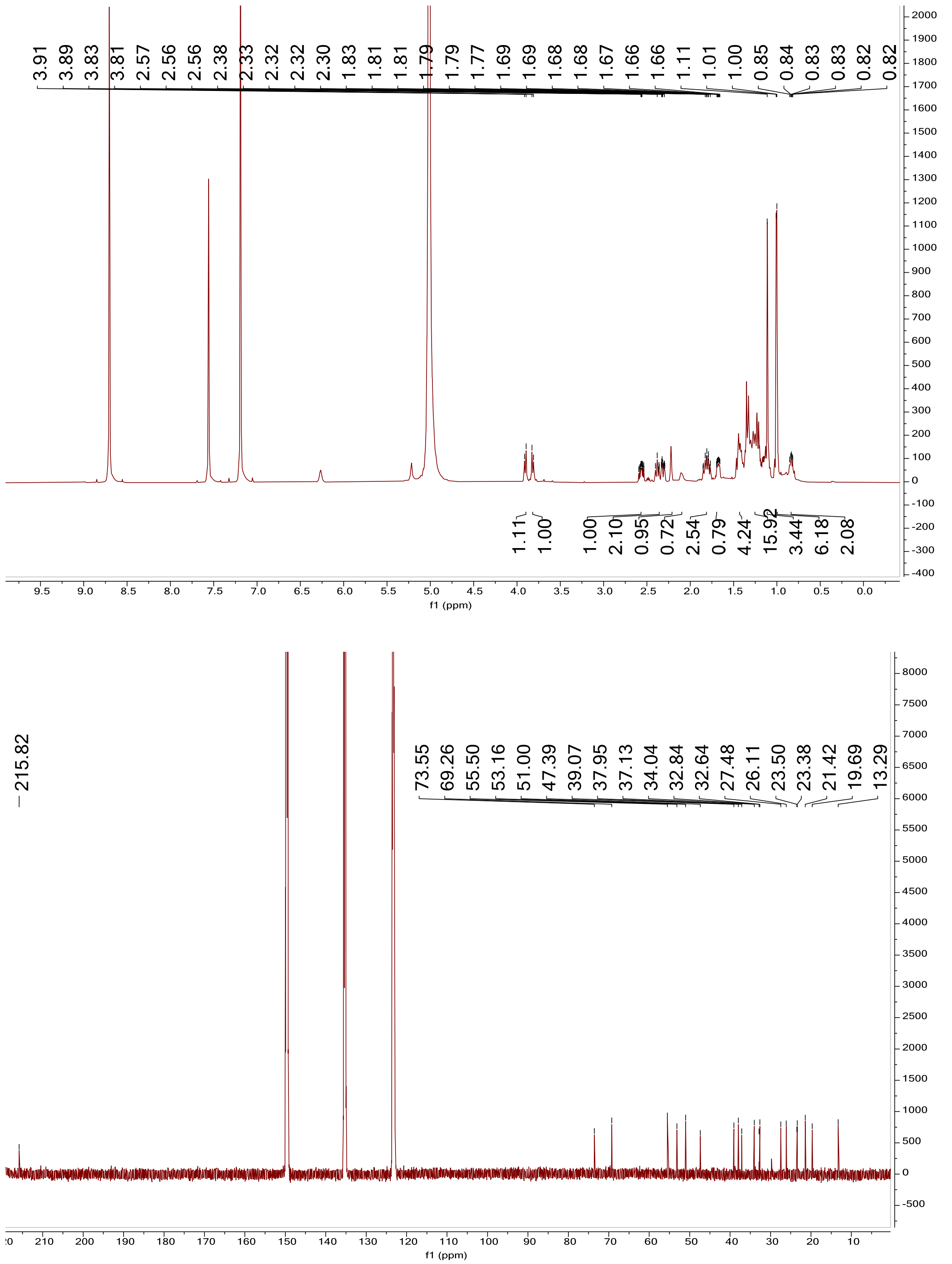

Figure S13. ^{1}H- and ^{13}C-NMR spectra of compound **5** (600 and 150 MHz, Pyr-d_5).

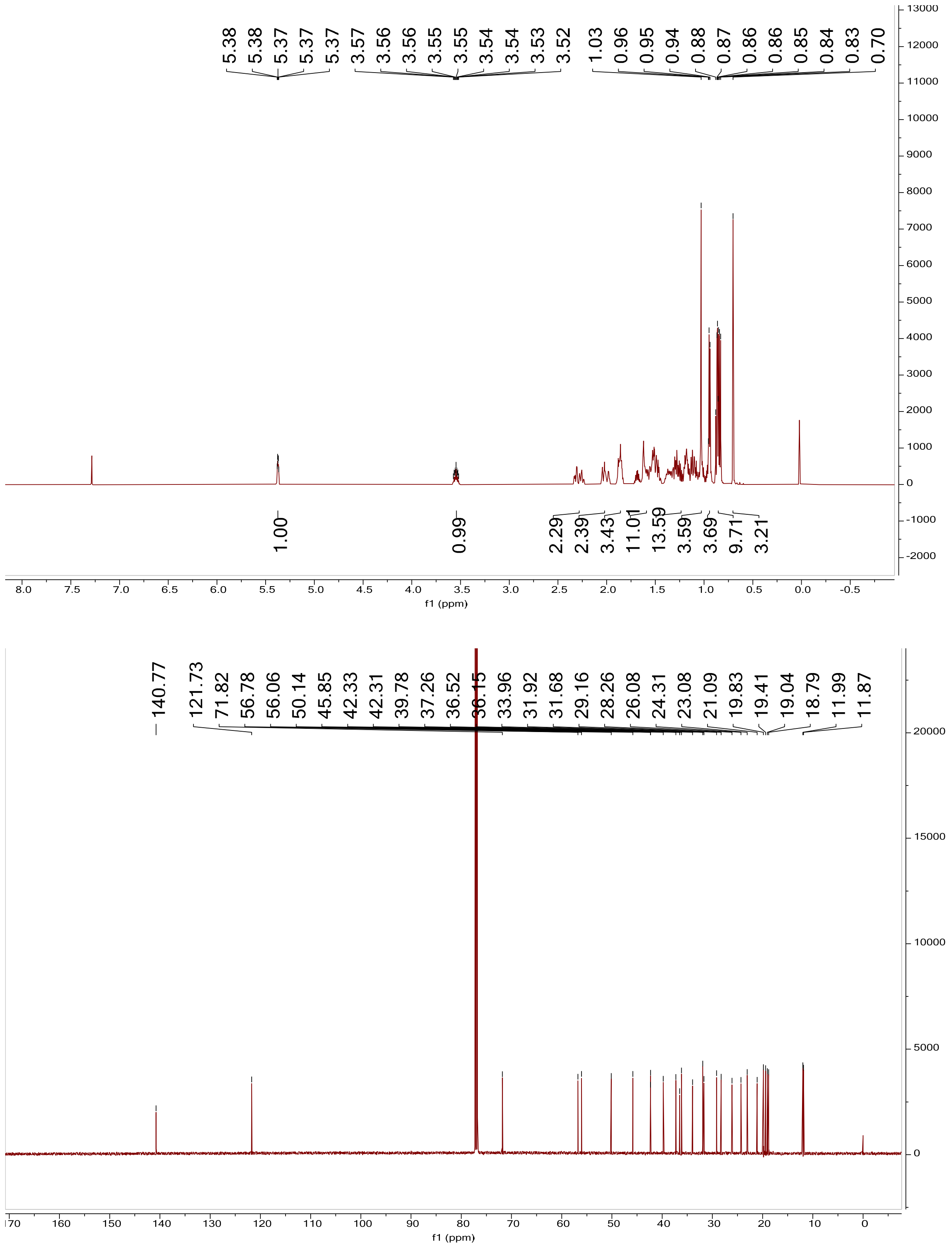

Figure S14. ^{1}H- and ^{13}C-NMR spectra of compound **6** (600 and 150 MHz, $CDCl_3$).

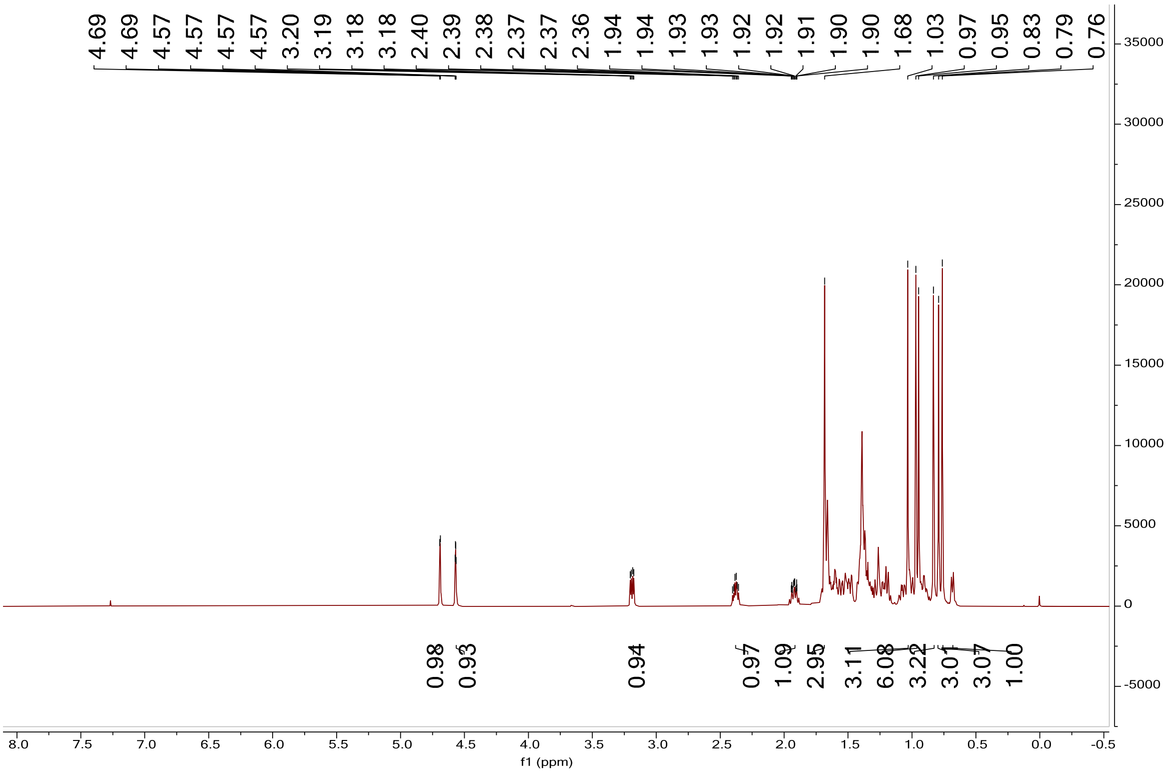

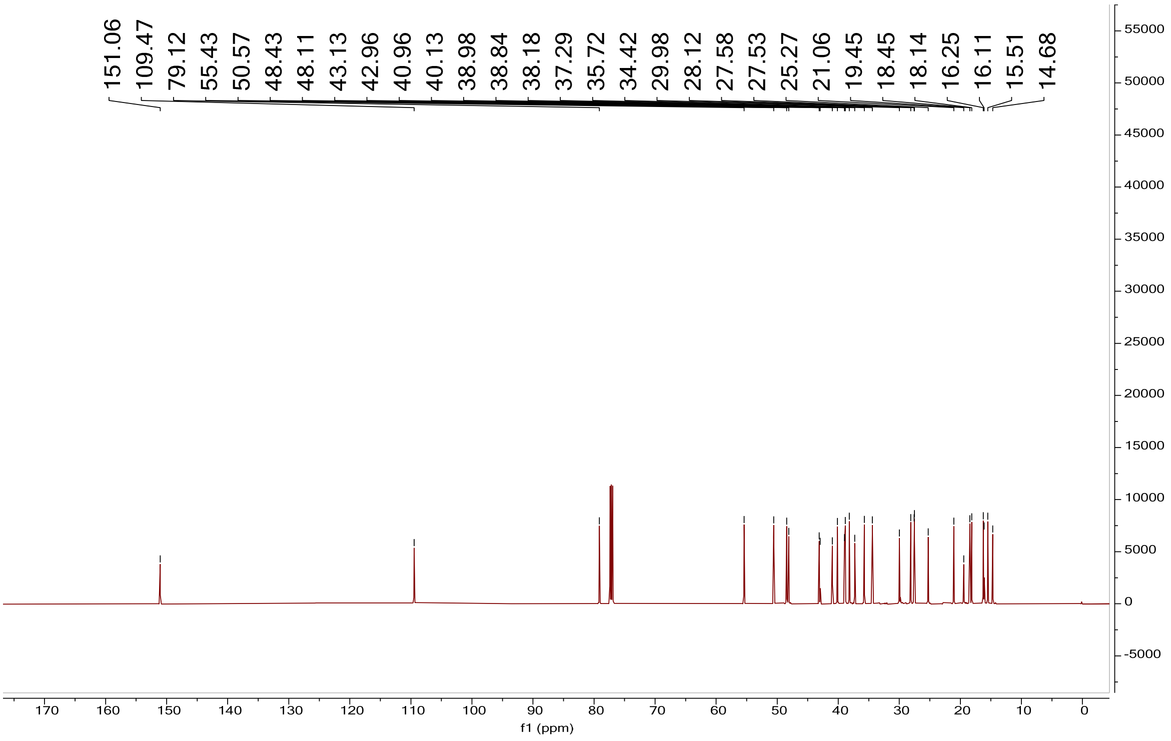

Figure S15. ^1H- and ^{13}C-NMR spectra of compound **7** (600 and 150 MHz, $CDCl_3$).

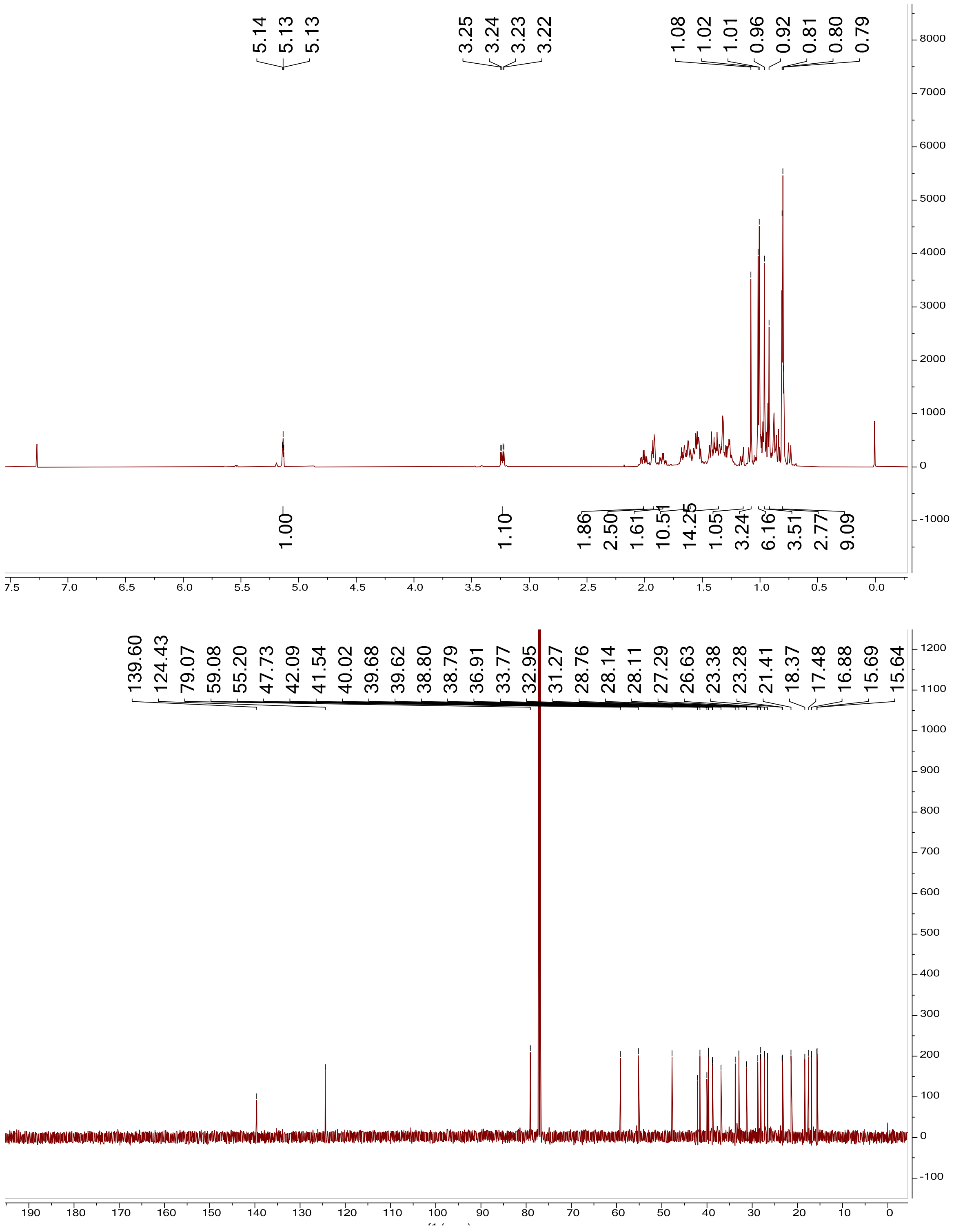

Figure S16. ^{1}H- and ^{13}C-NMR spectra of compound **8** (600 and 150 MHz, $CDCl_3$).

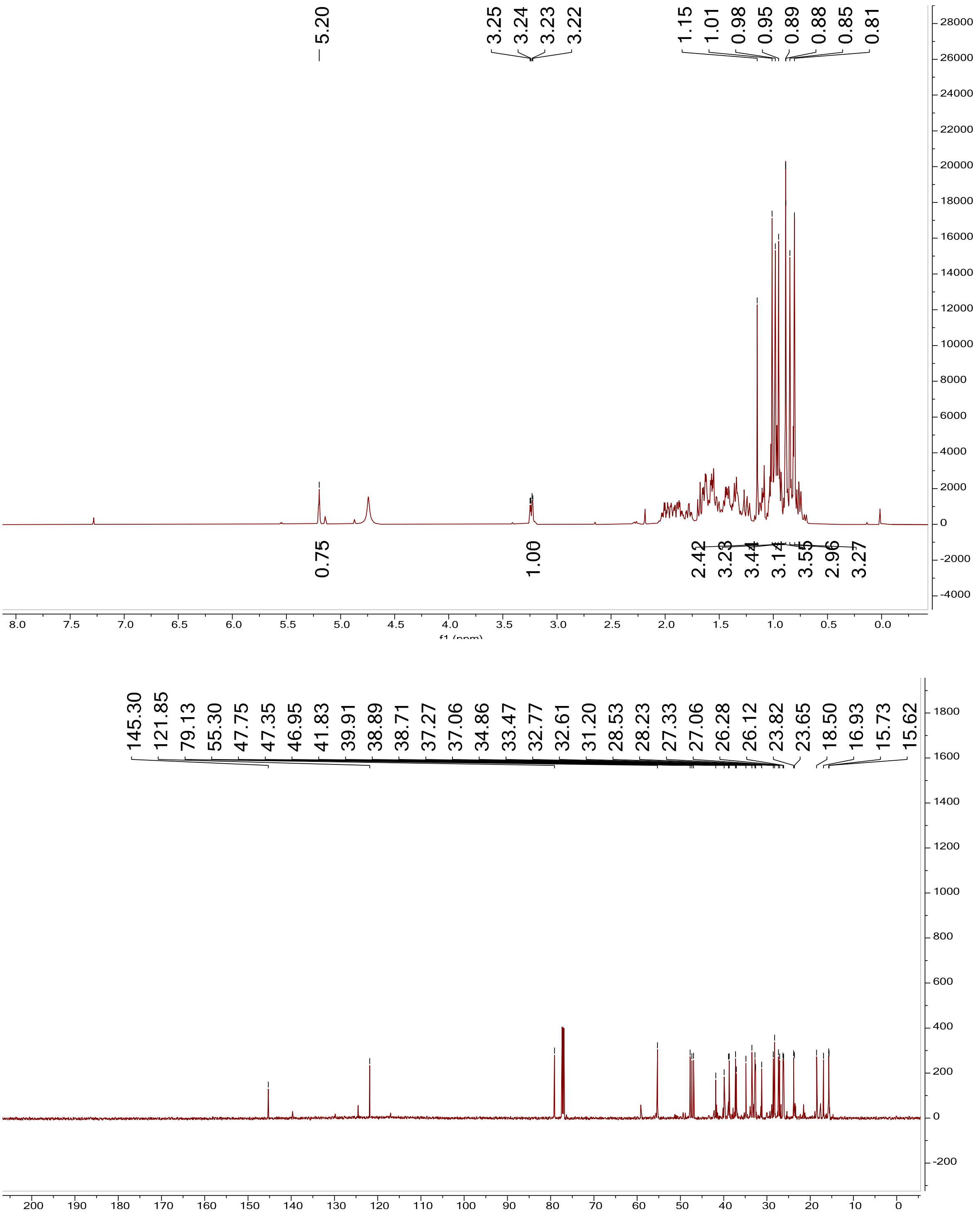

Figure S17. ^{1}H- and ^{13}C-NMR spectra of compound **9** (600 and 150 MHz, $CDCl_3$).

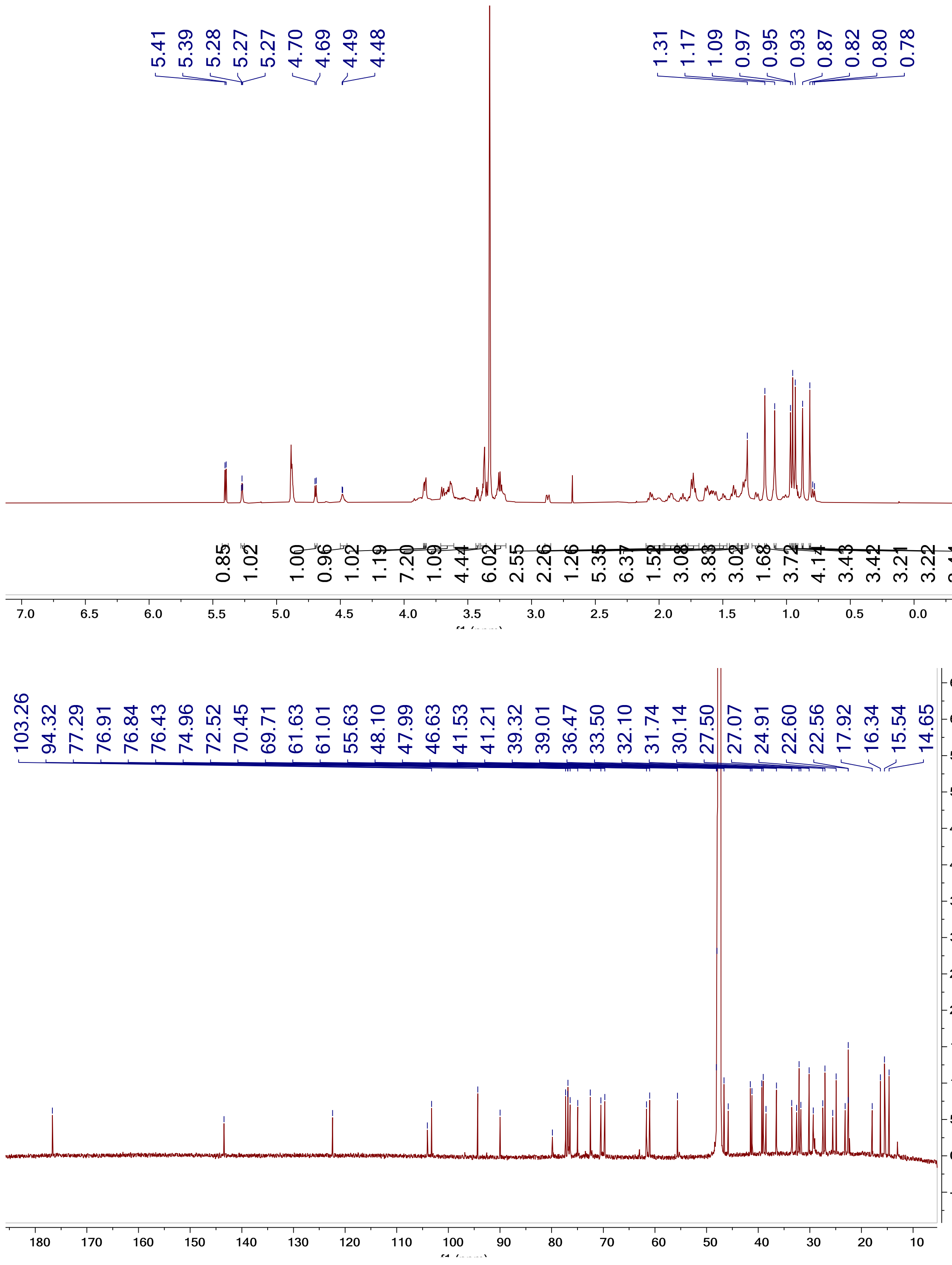

Figure S18. ^{1}H- and ^{13}C-NMR spectra of compound **10** (800 and 200 MHz, CD_3OD).

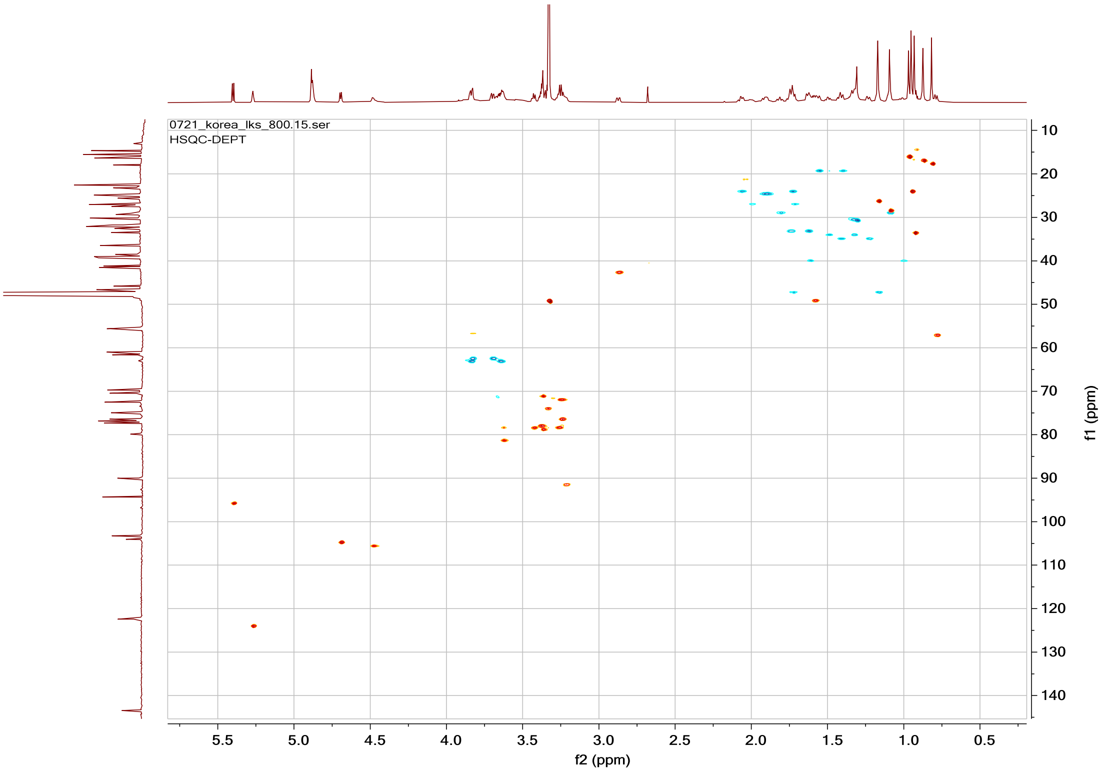

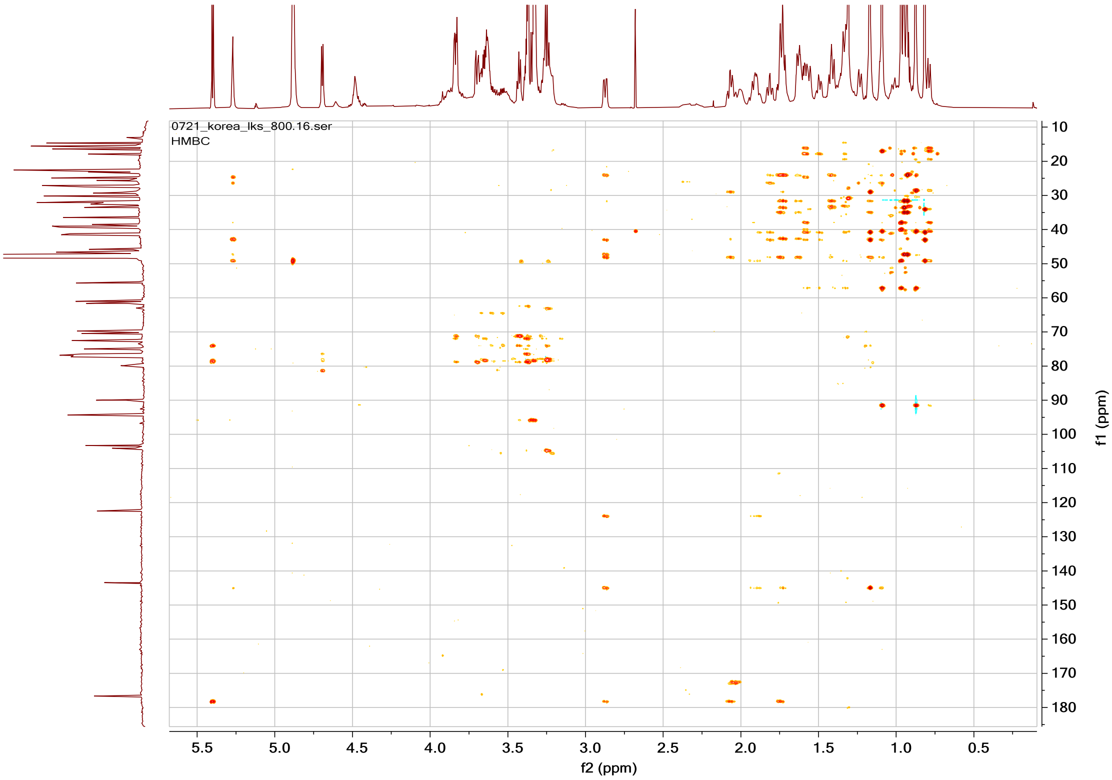

Figure S19. HSQC and HMBC spectra of compound **10** (800 MHz, CD_3OD).

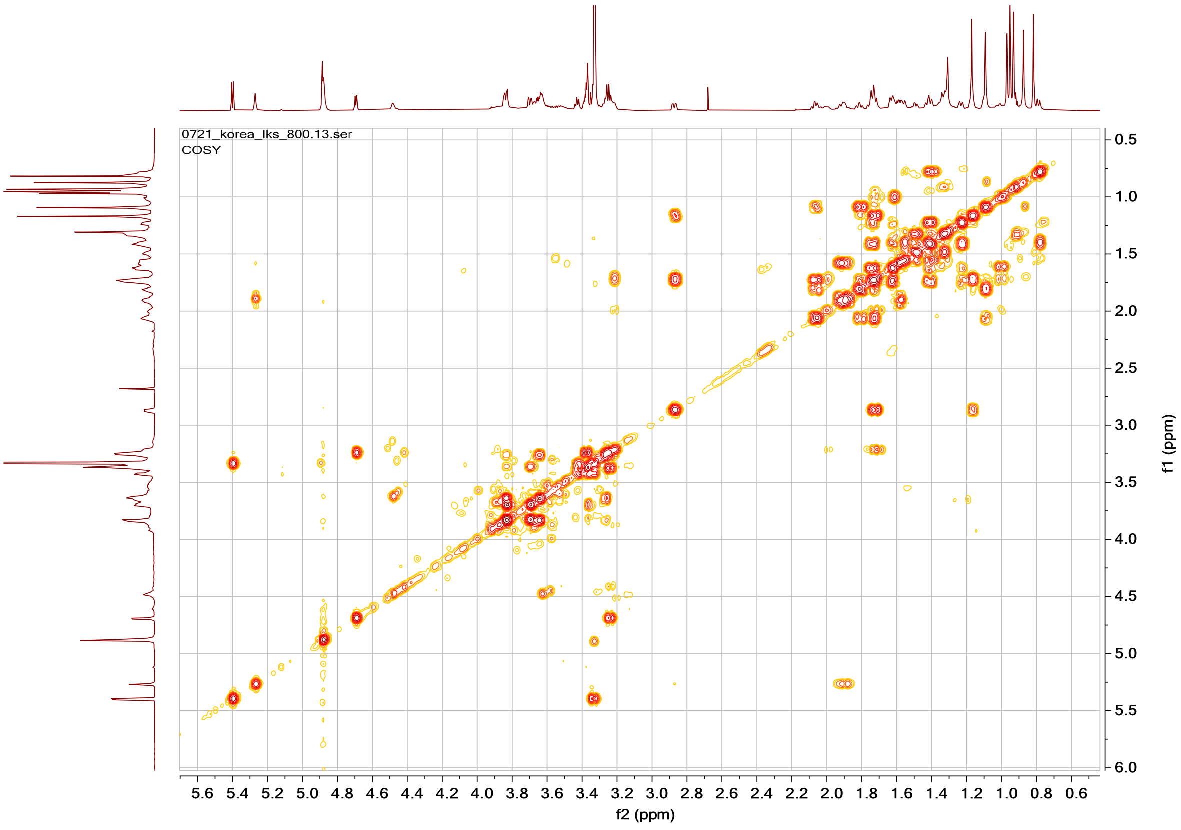

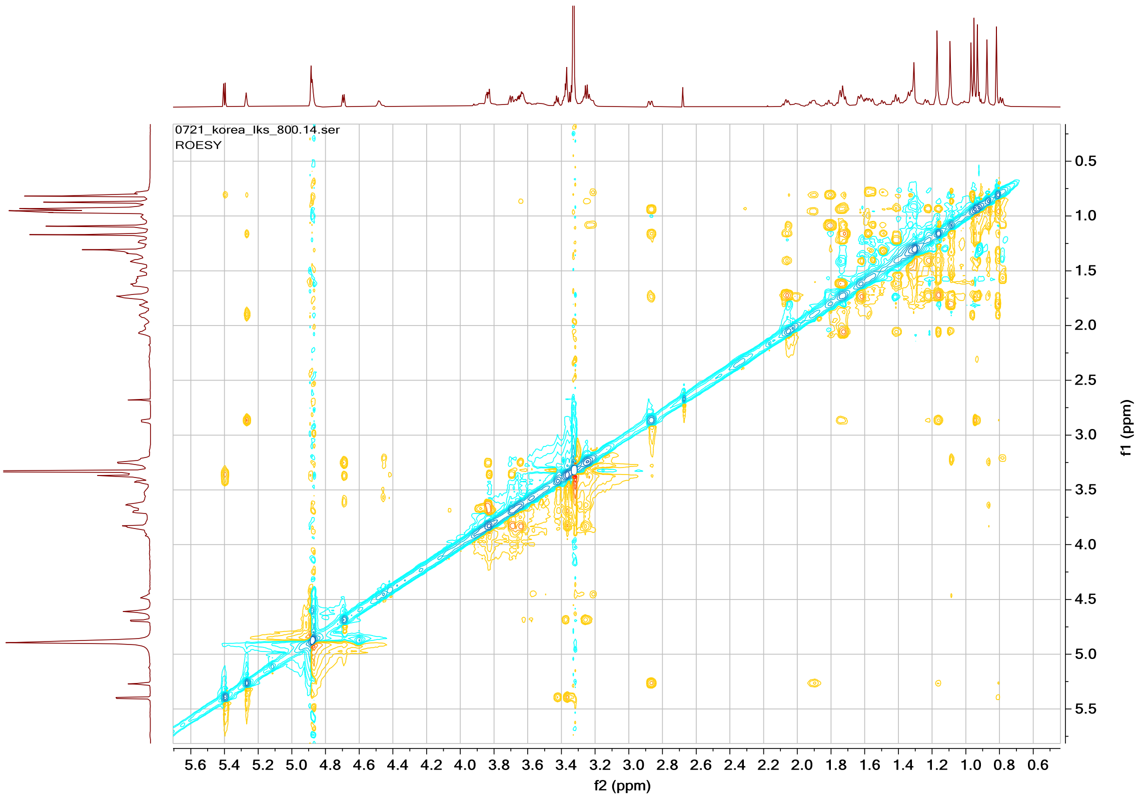

Figure S20. COSY and NOESY spectra of compound **10** (800 MHz, CD_3OD).

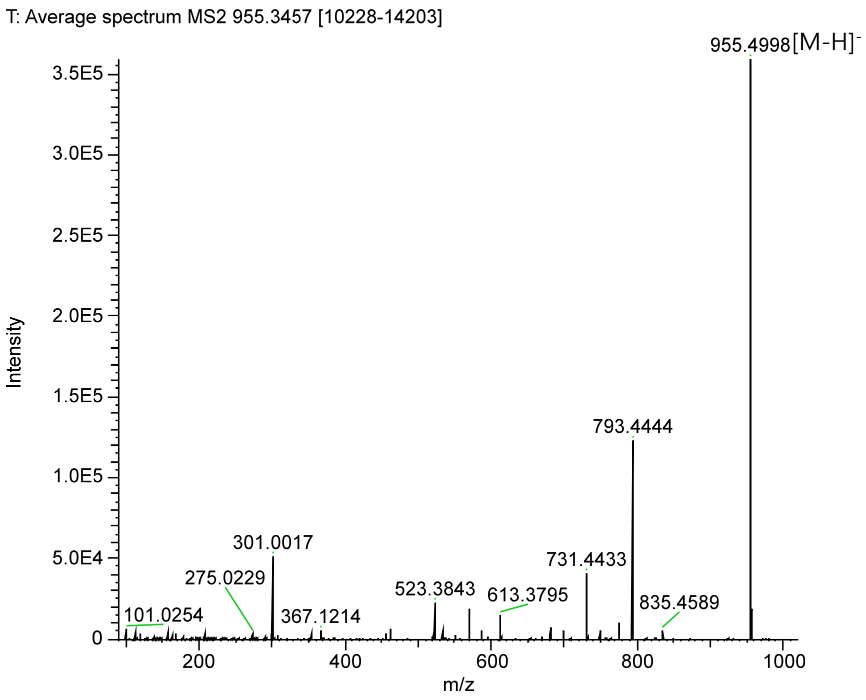

Figure S21. HRESIMS spectrum of compound **10.**

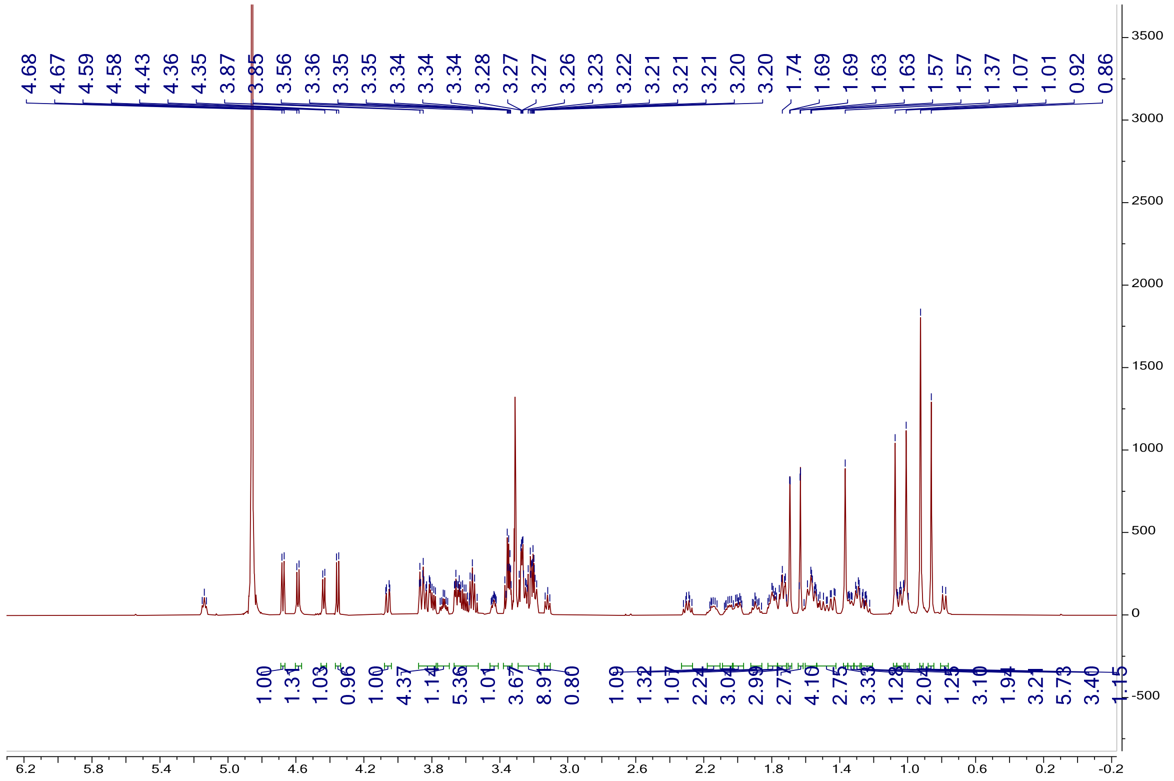

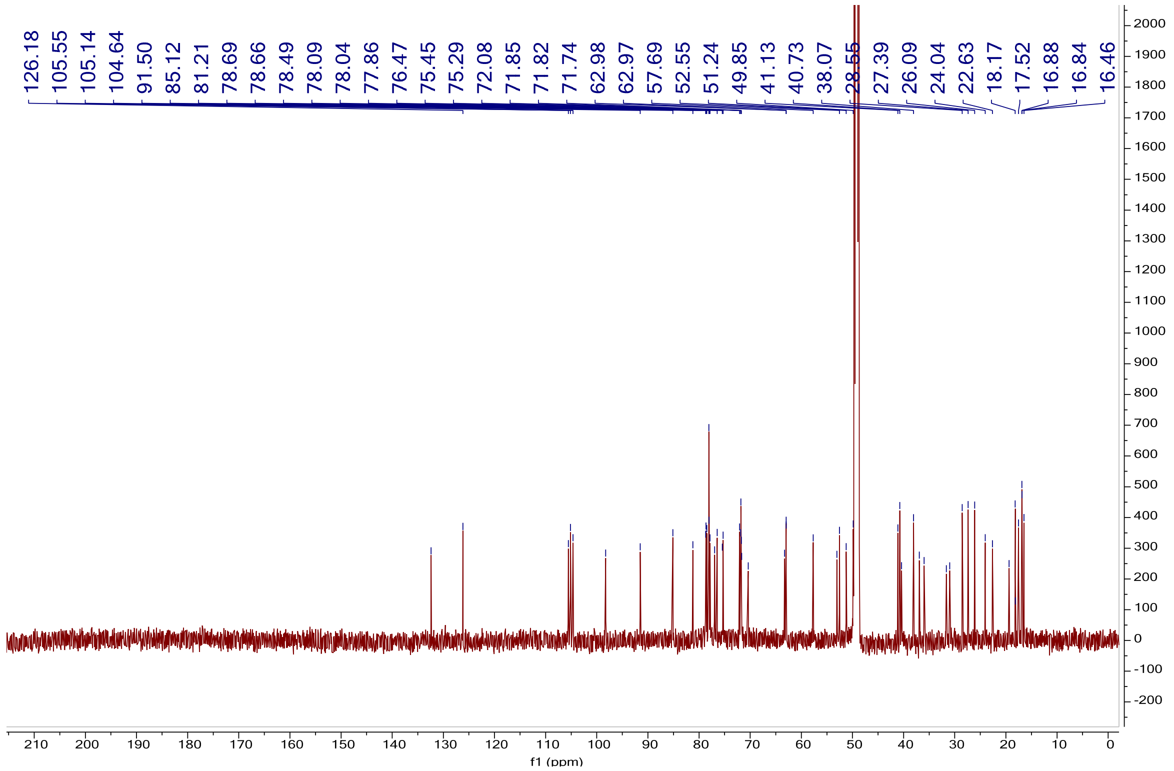

Figure S22. ^{1}H- and ^{13}C-NMR spectra of compound **11** (600 and 150 MHz, CD_3OD).

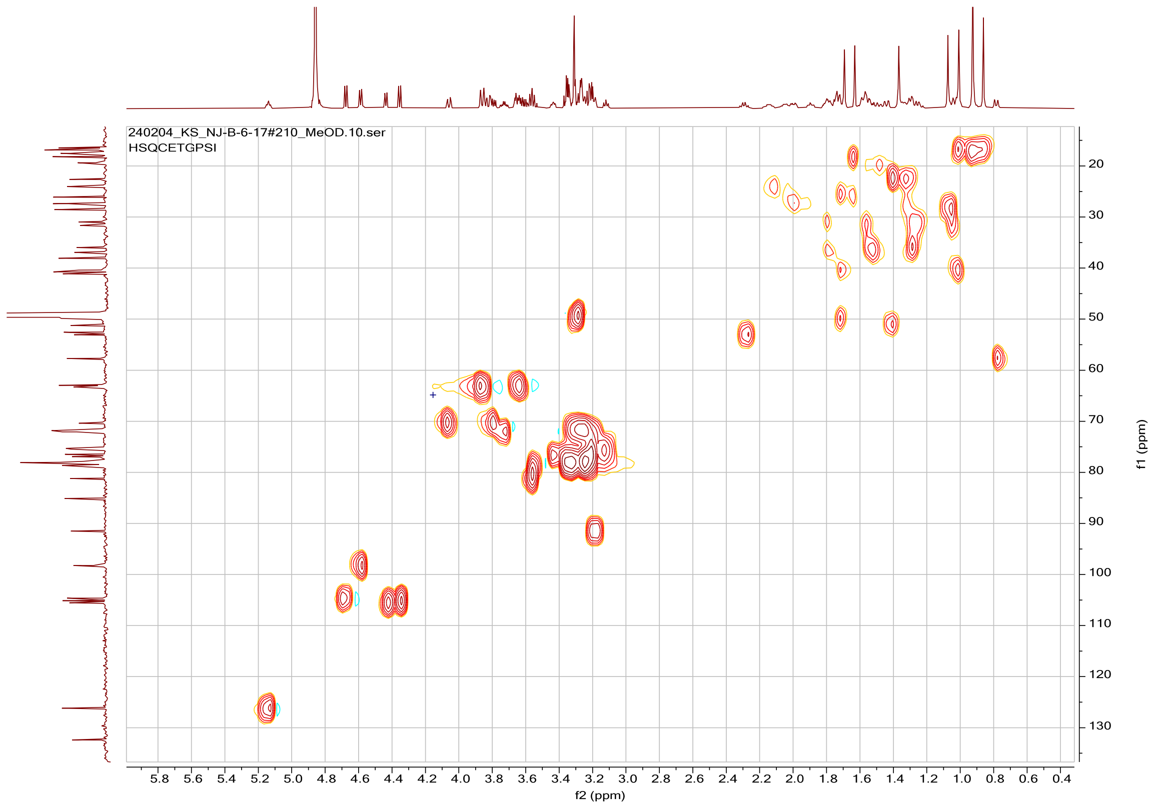

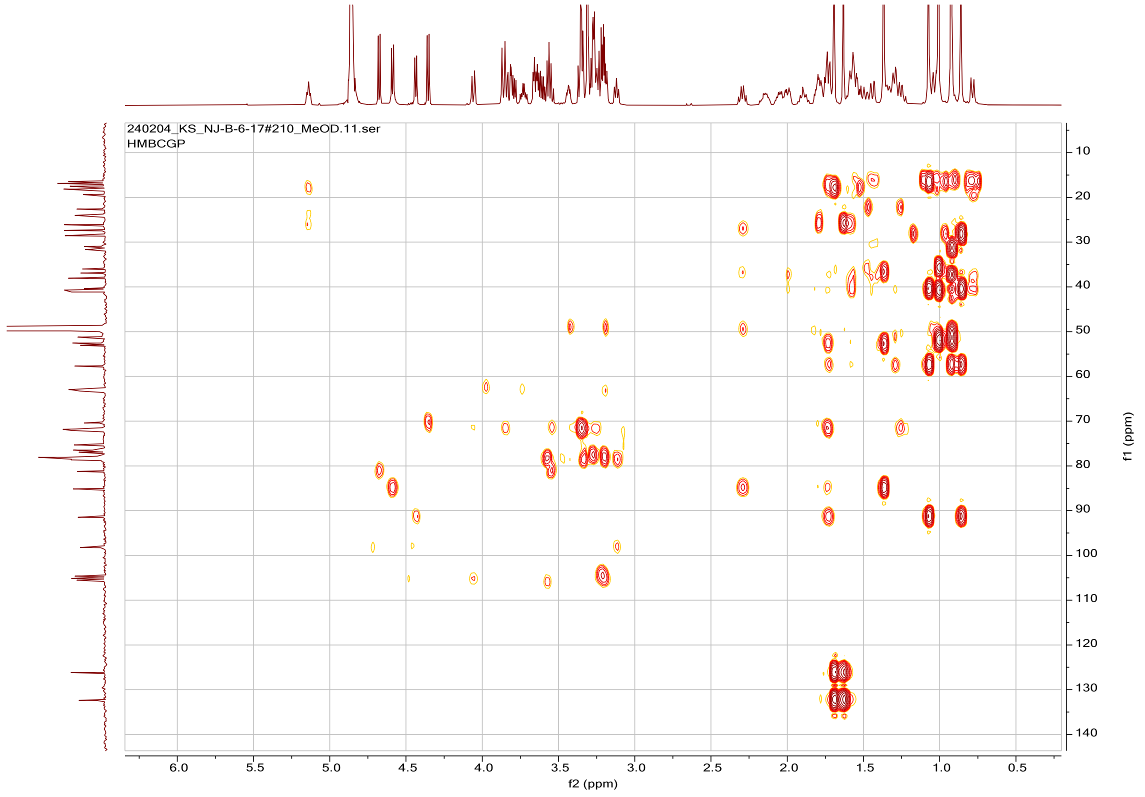

Figure S23. HSQC and HMBC spectra of compound **11** (600 MHz, CD_3OD).

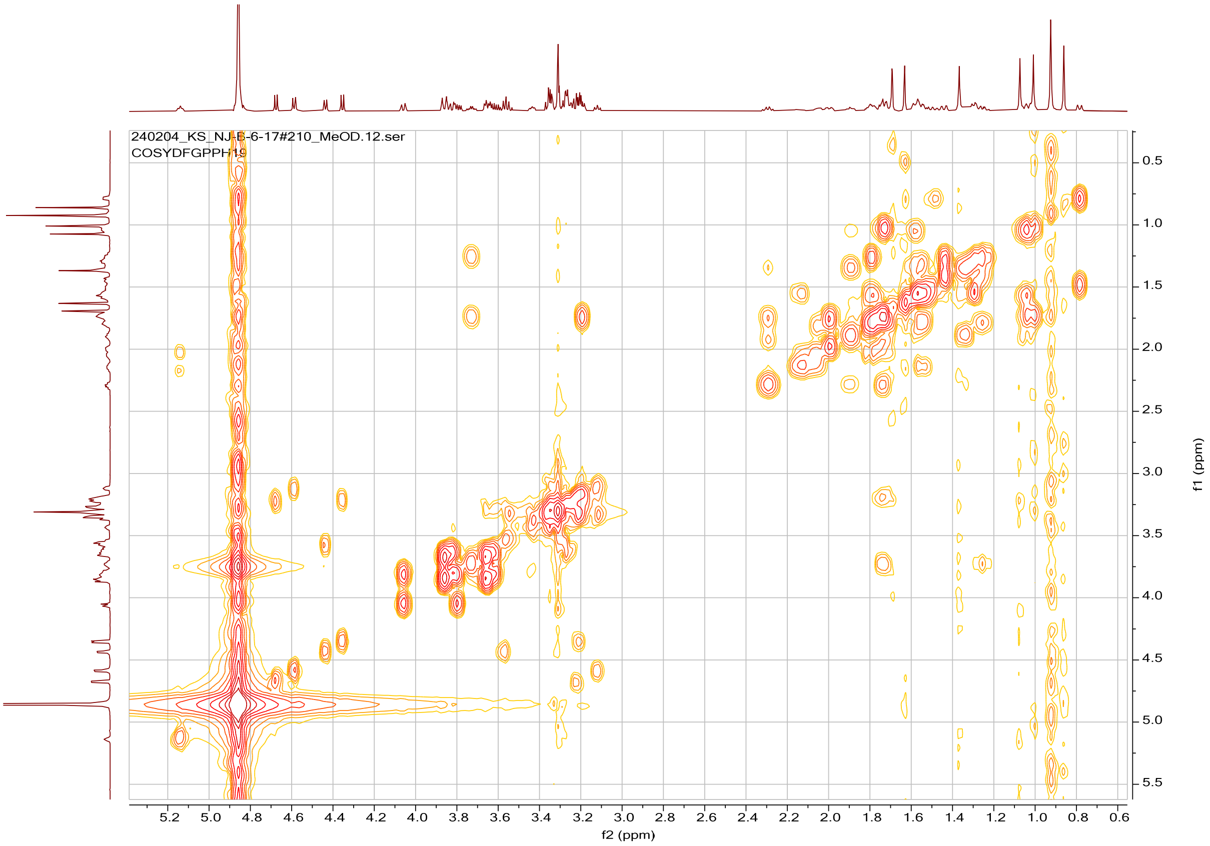

Figure S24. COSY spectrum of compound **11** (600 MHz, CD_3OD).

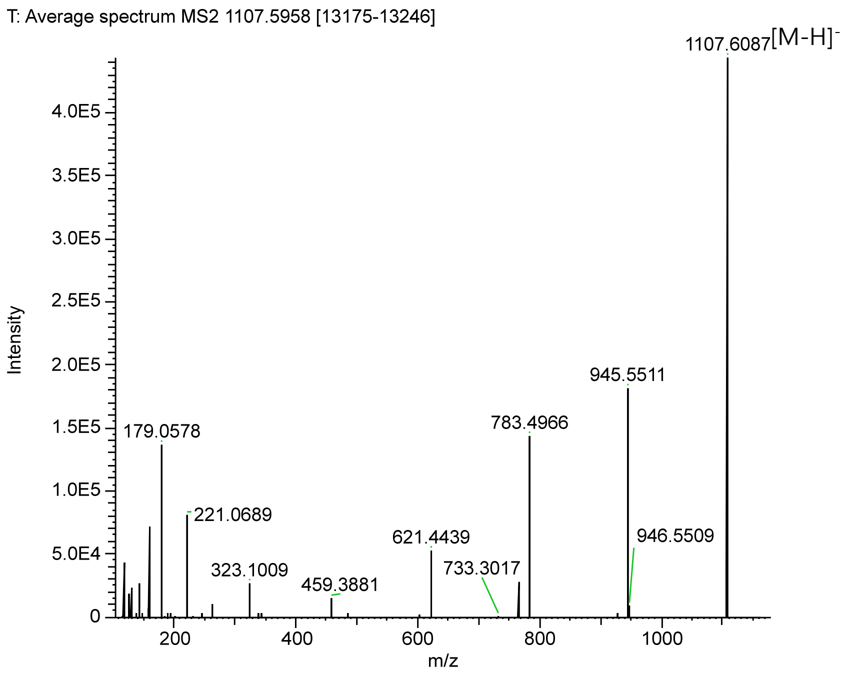

Figure S25. HRESIMS spectrum of compound **11.**

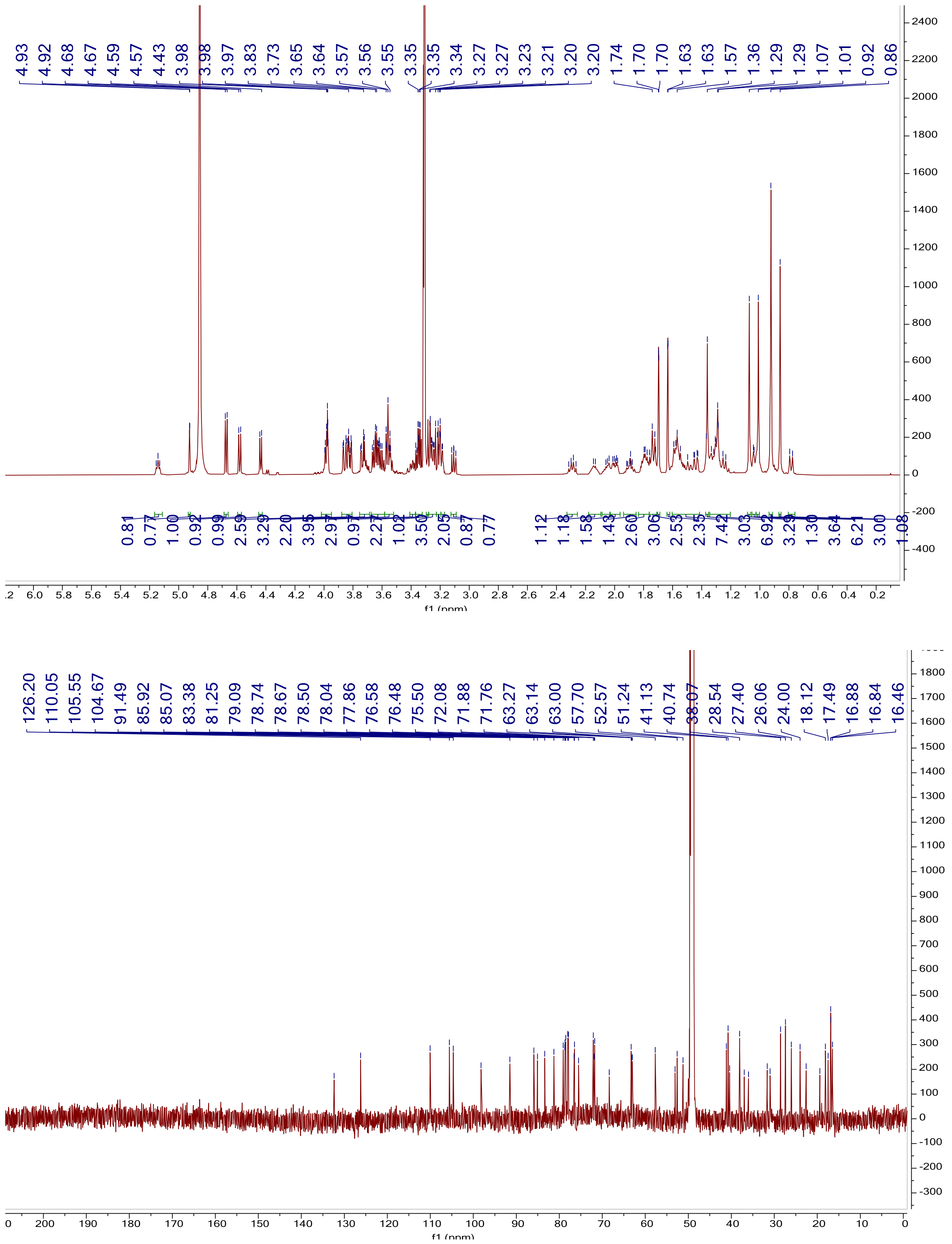

Figure S26. ^{1}H- and ^{13}C-NMR spectra of compound **12** (600 and 150 MHz, CD_3OD).

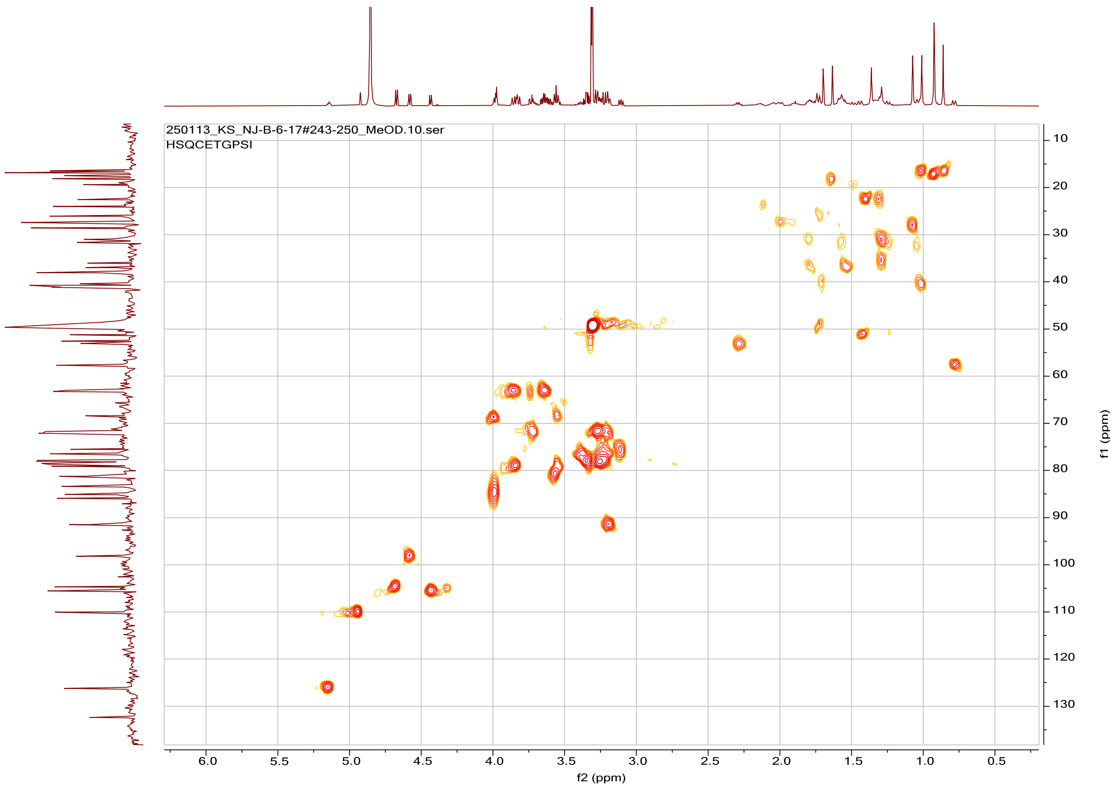

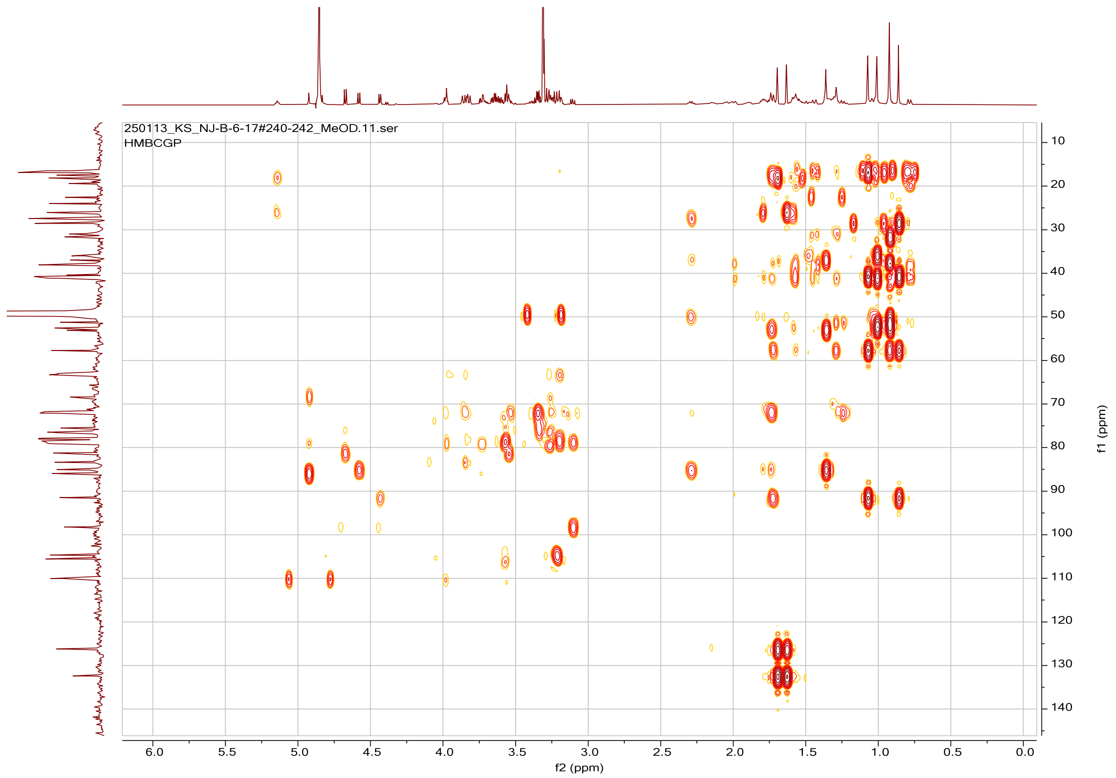

Figure S27. HSQC and HMBC spectra of compound **12** (600 MHz, CD_3OD).

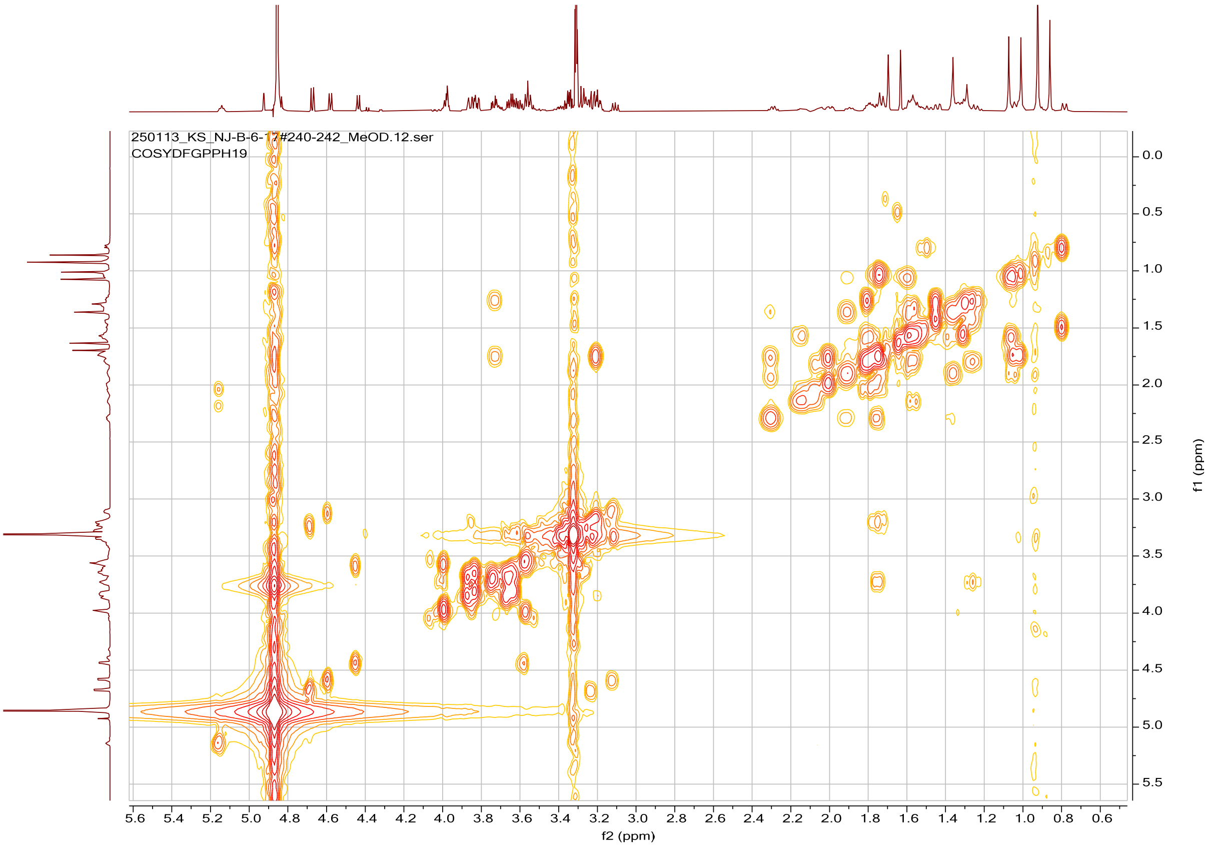

Figure S28. COSY spectrum of compound **12** (600 MHz, CD_3OD).

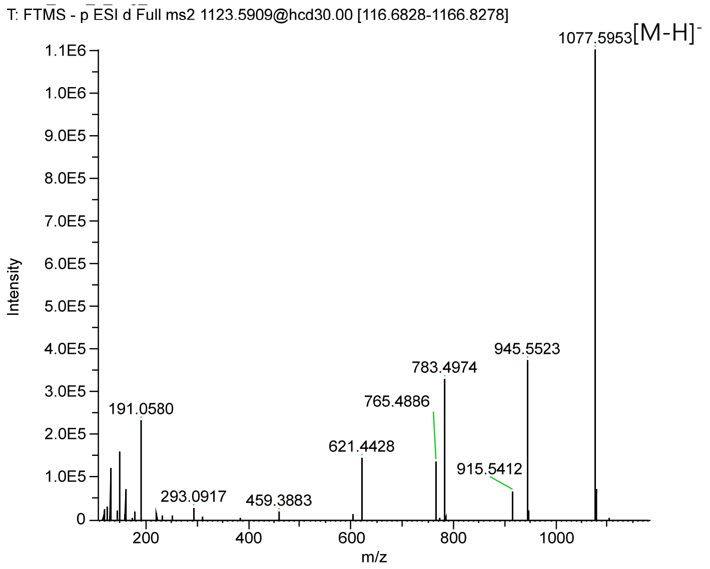

Figure S29. HRESIMS spectrum of compound **12.**

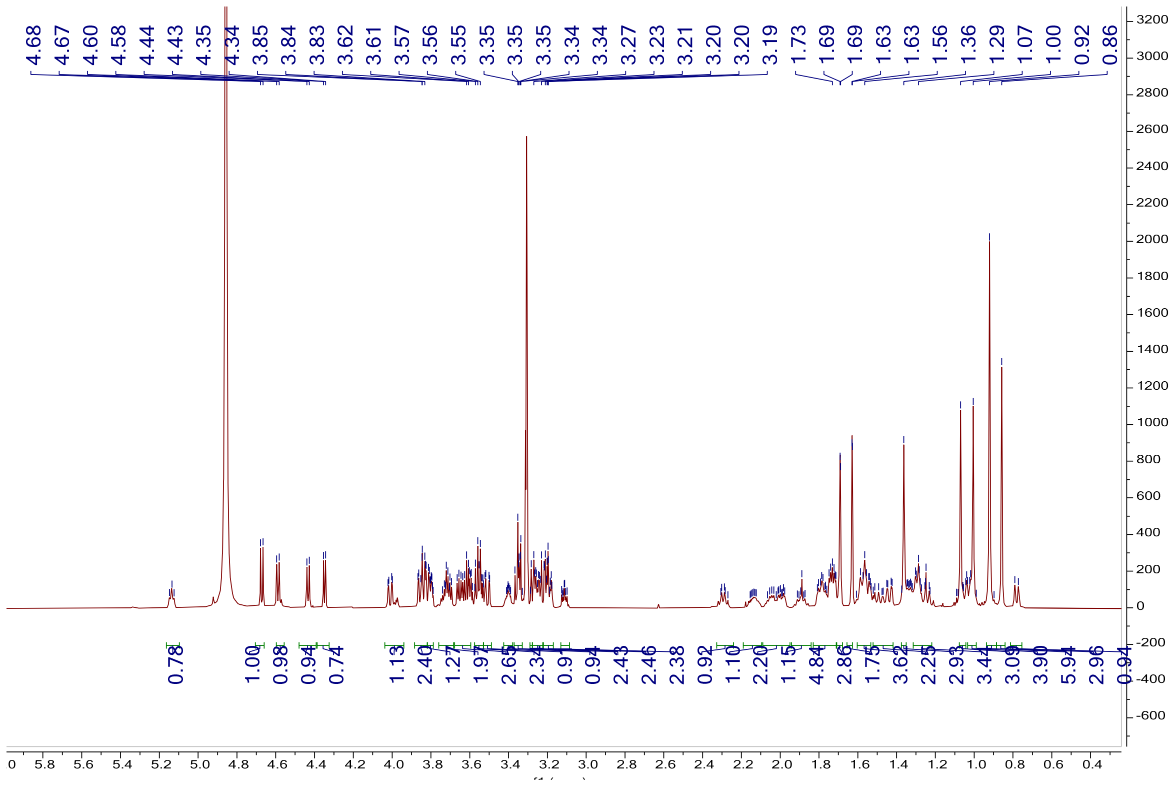

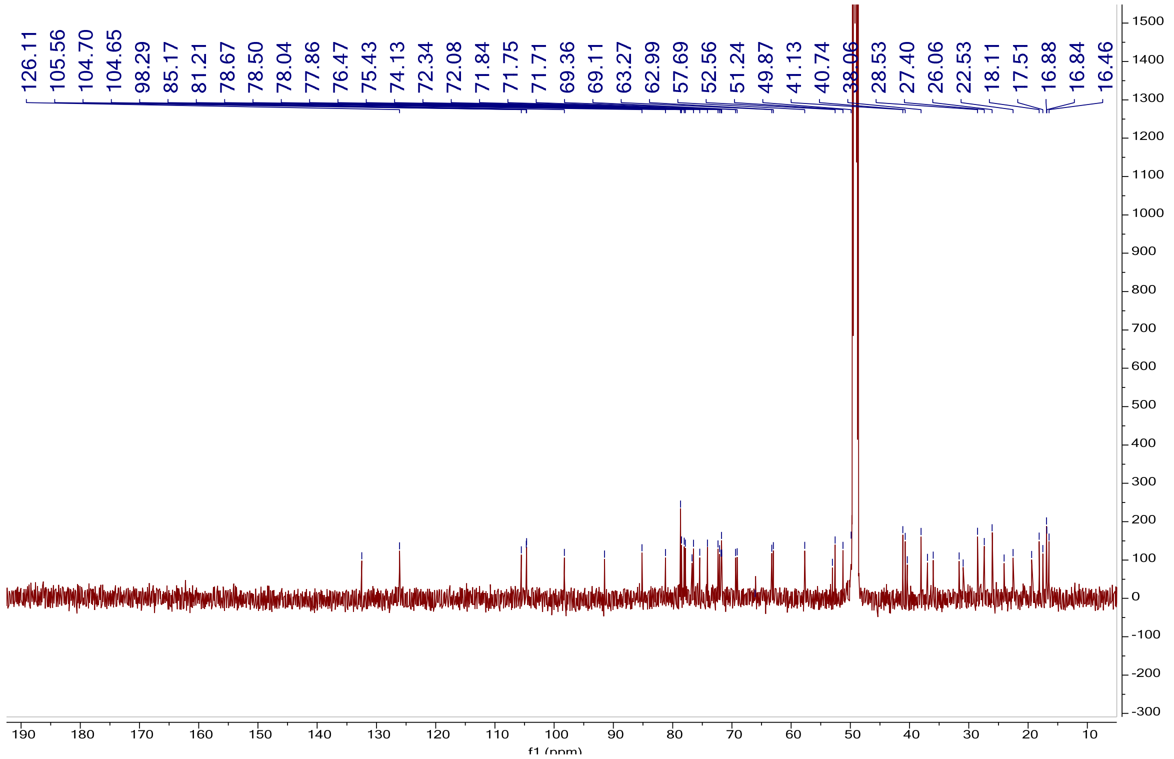

Figure S30. ^{1}H- and ^{13}C-NMR spectra of compound **13** (600 and 150 MHz, CD_3OD).

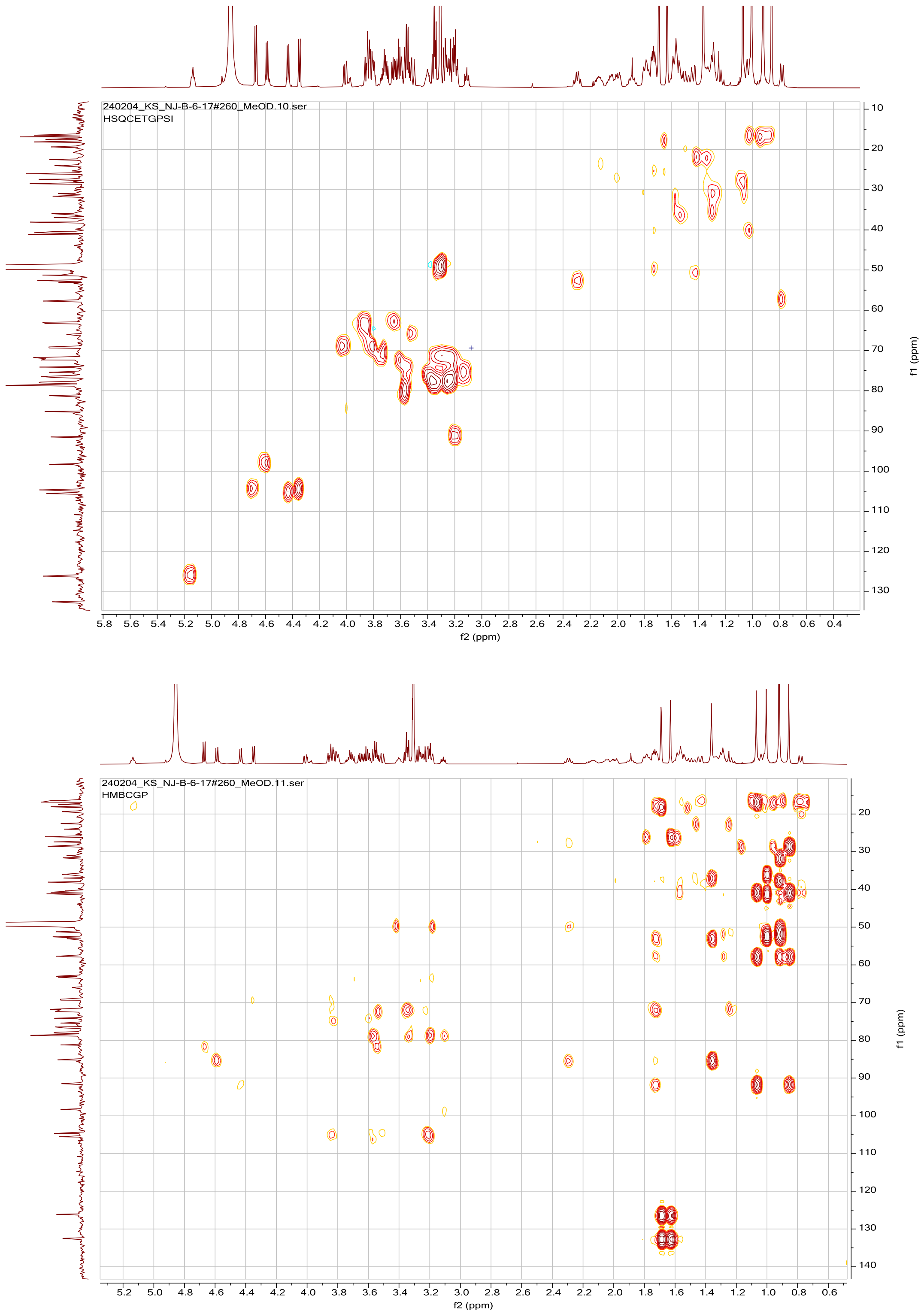

Figure S31. HSQC and HMBC spectra of compound **13** (600 MHz, CD_3OD).

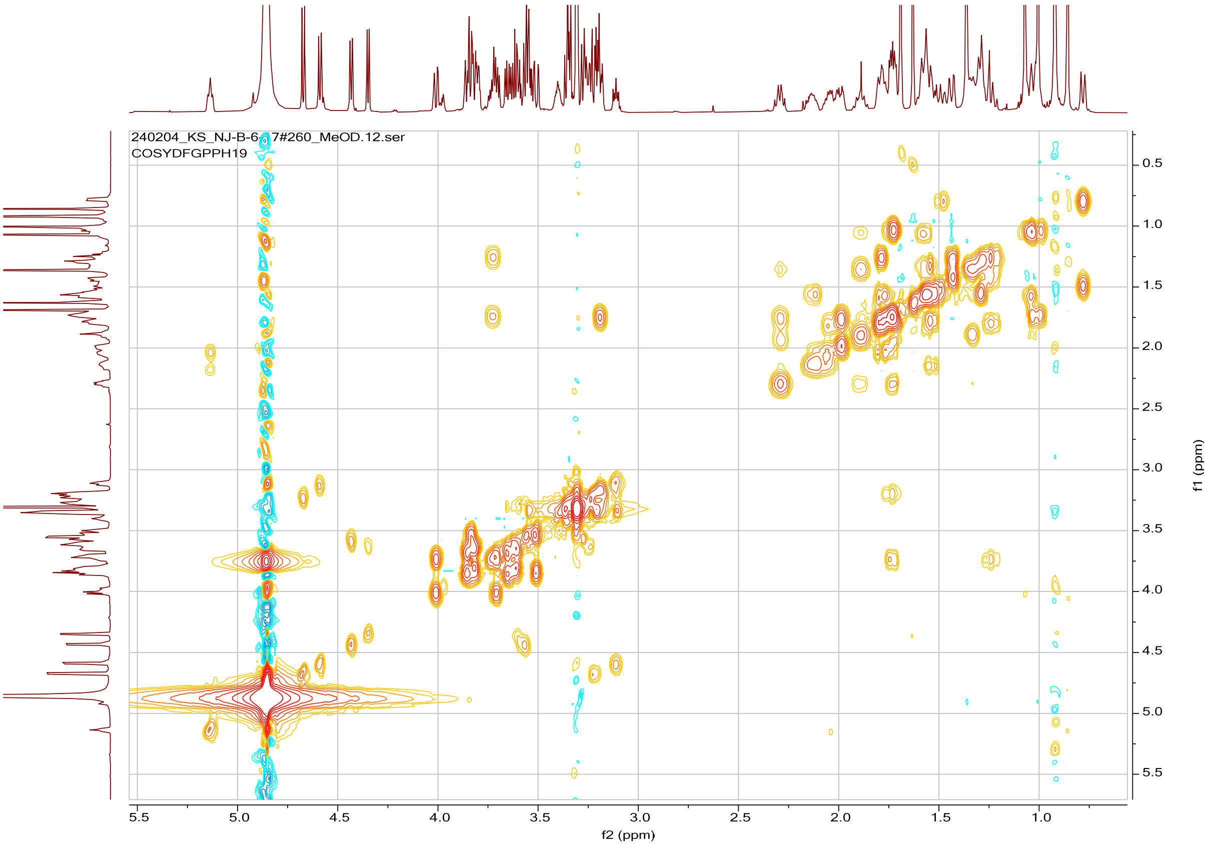

Figure S32. COSY spectrum of compound **13** (600 MHz, CD_3OD).

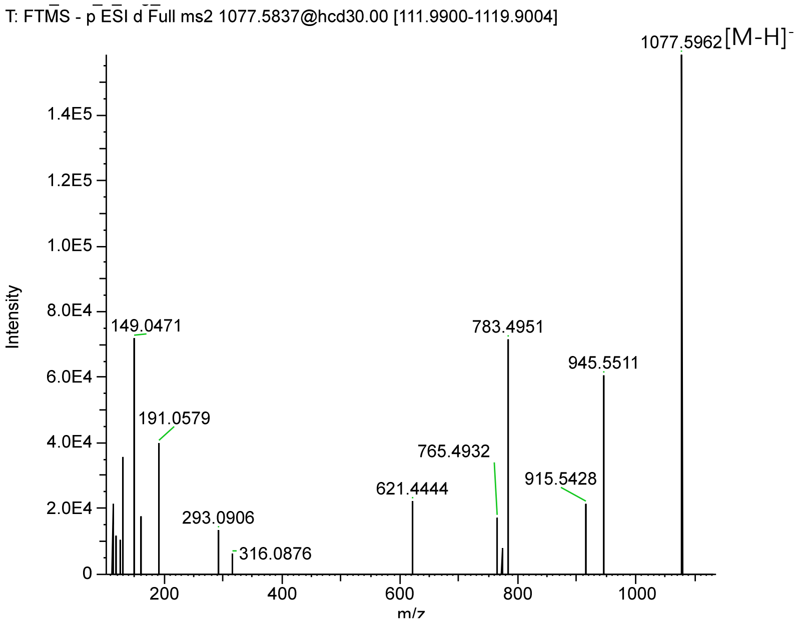

Figure S33. HRESIMS spectrum of compound **13**.

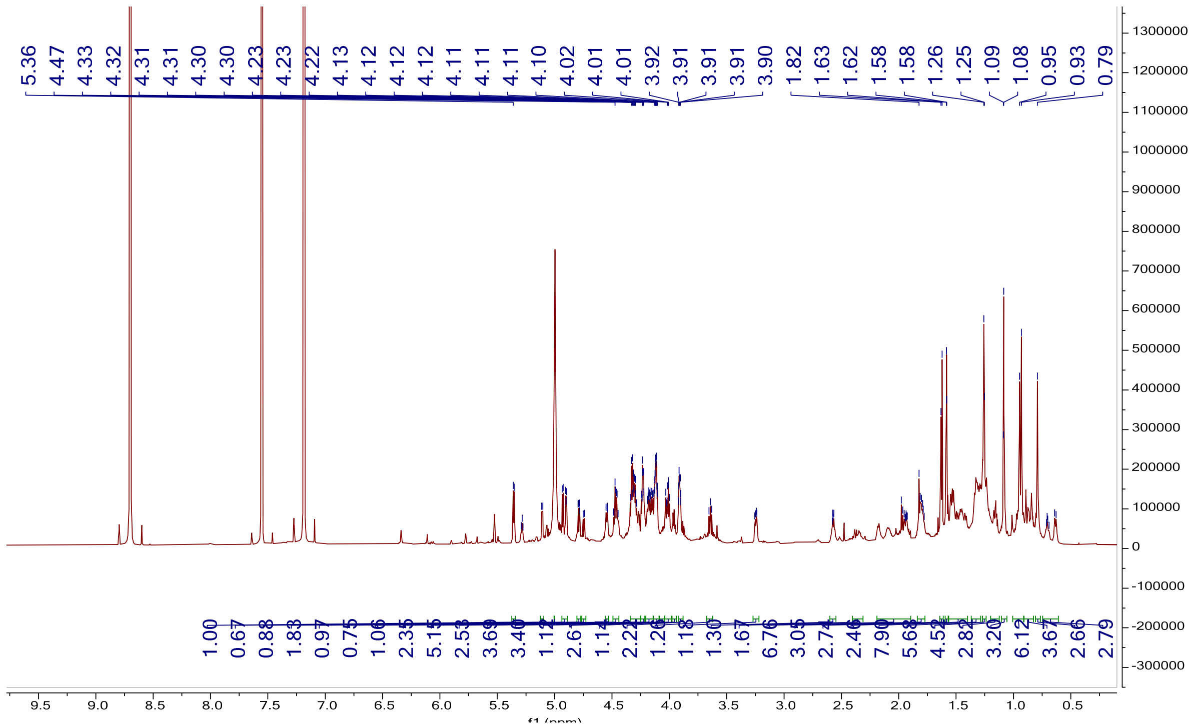

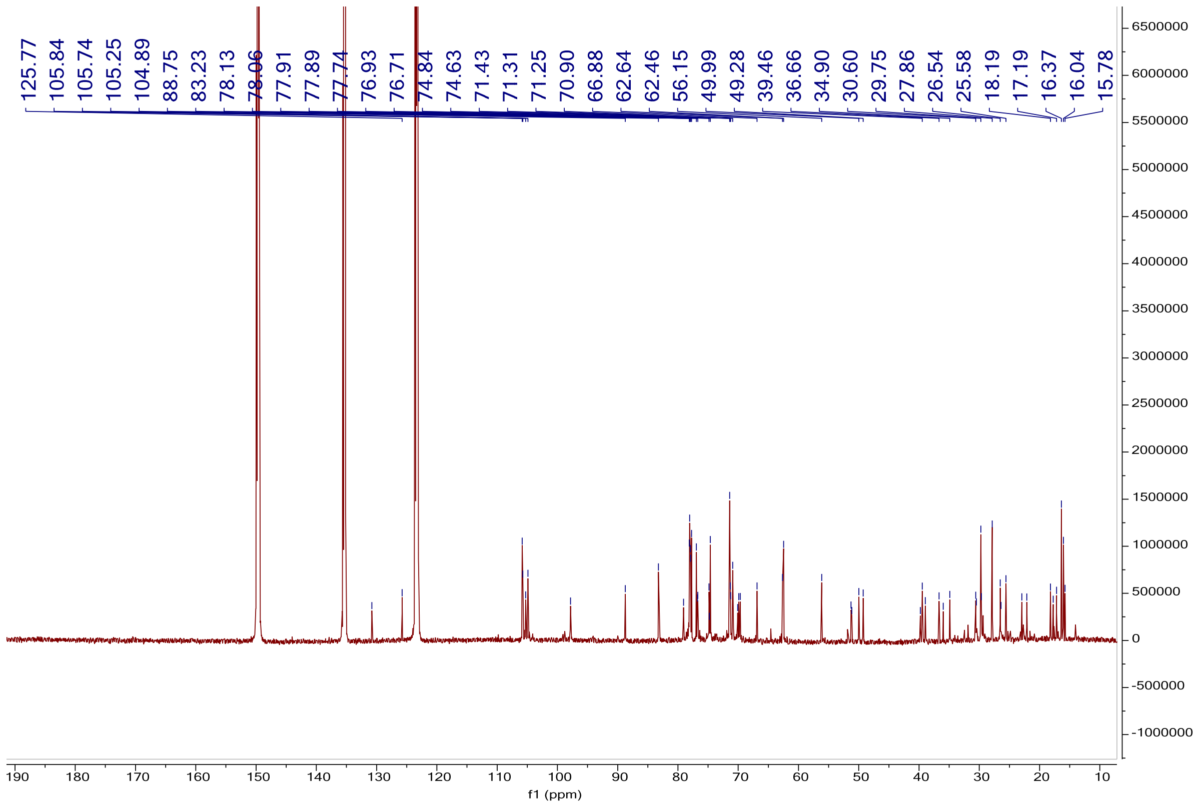

Figure S34. ^{1}H- and ^{13}C-NMR spectra of compound **14** (800 and 200 MHz, pyr-d_5).

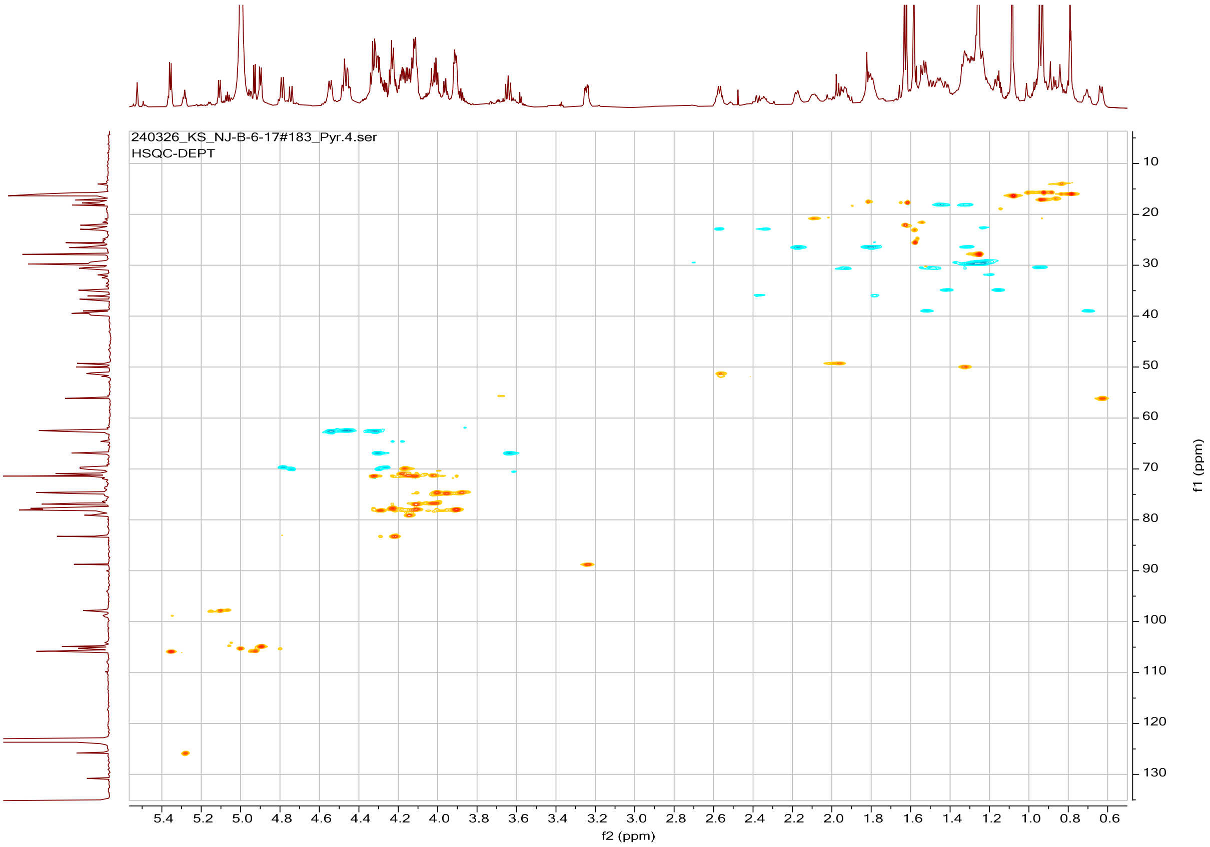

Figure S35. HSQC spectrum of compound **14** (800 MHz, pyr-d_5).

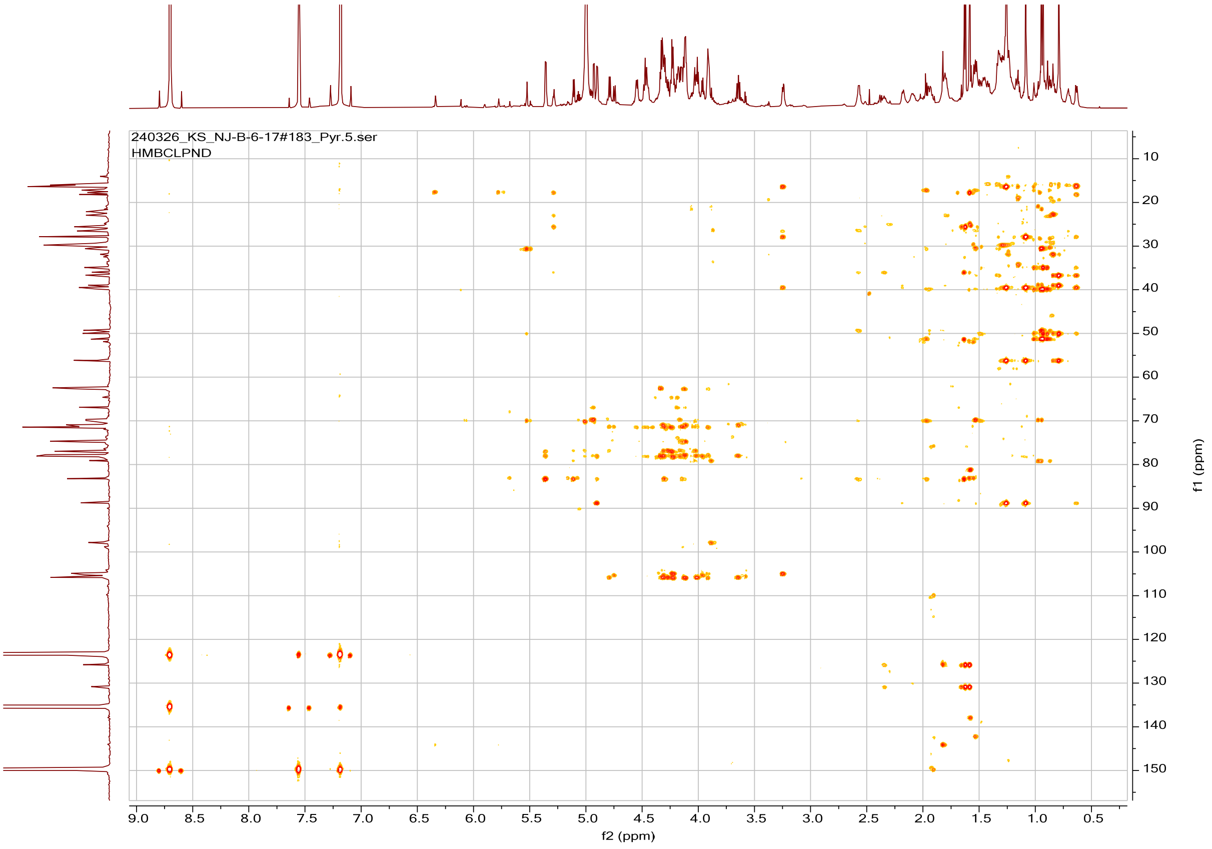

Figure S36. HMBC spectrum of compound **14** (800 MHz, pyr-d5).

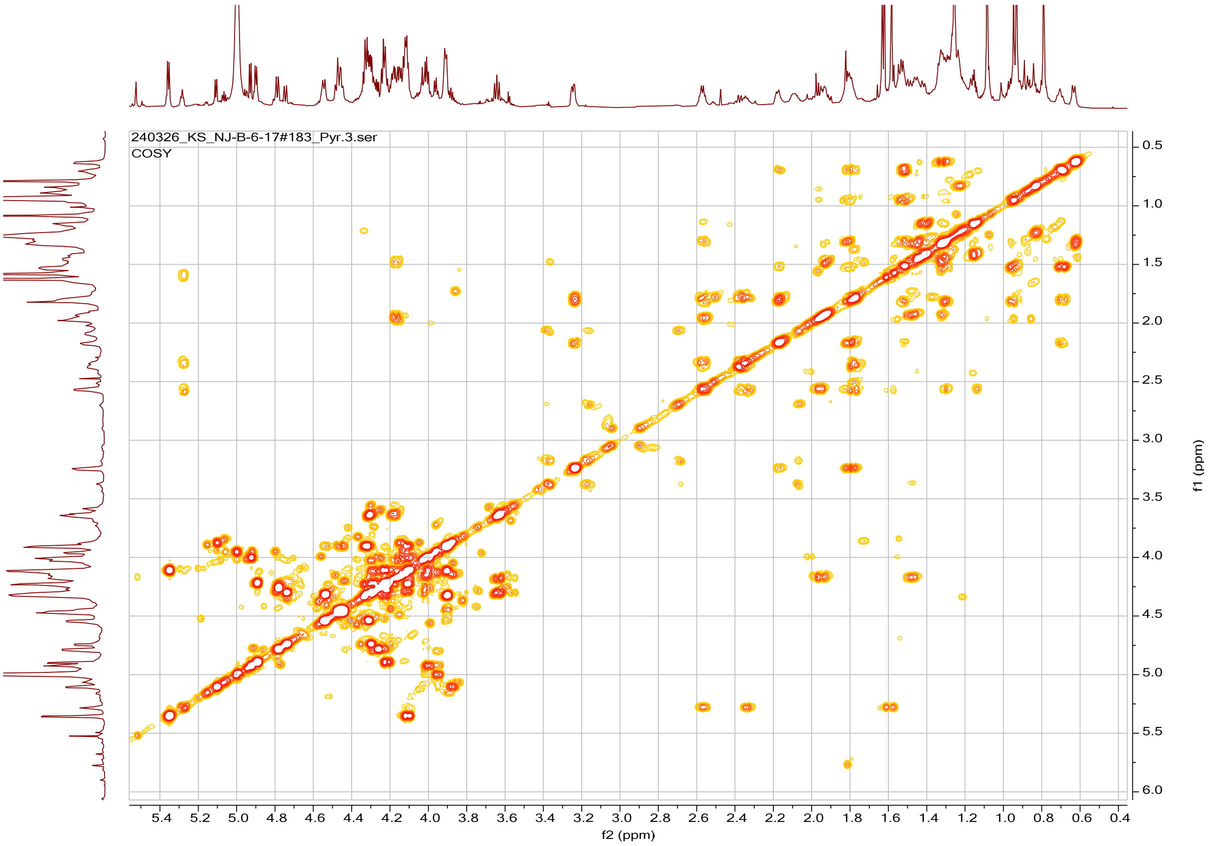

Figure S37. COSY spectrum of compound **14** (800 MHz, pyr-d5).

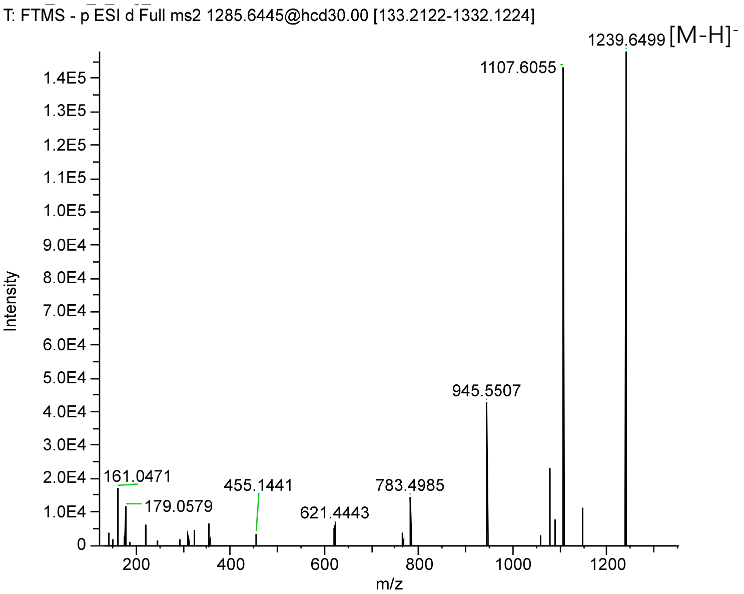

Figure S38. HRESIMS spectrum of compound **14**.

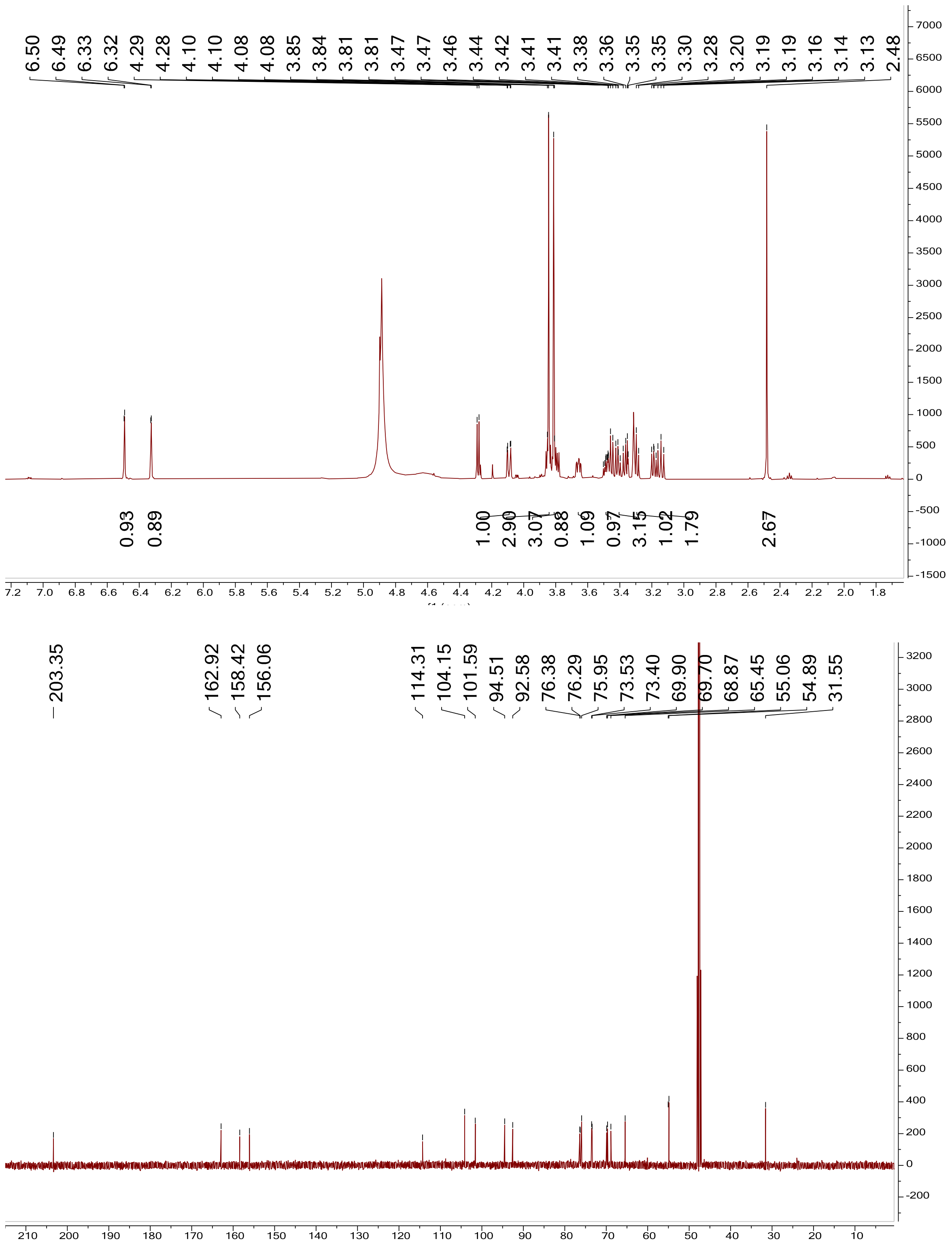

Figure S39. ^{1}H- and ^{13}C-NMR spectra of compound **15** (600 and 150 MHz, Pyr-d_5).

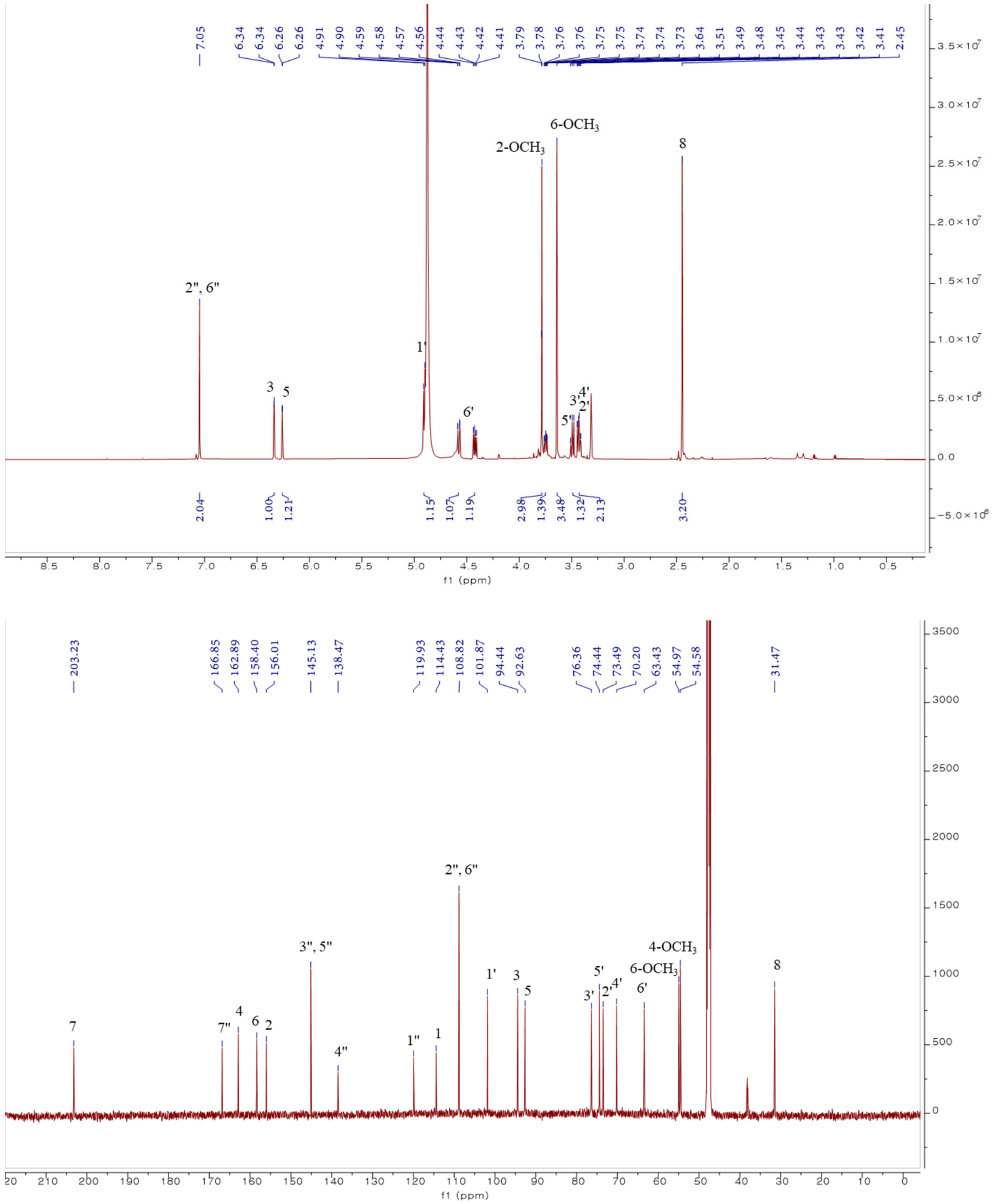

Figure S40. ^{1}H- and ^{13}C-NMR spectra of compound **16** (600 and 150 MHz, CD_3OD+D_2O).

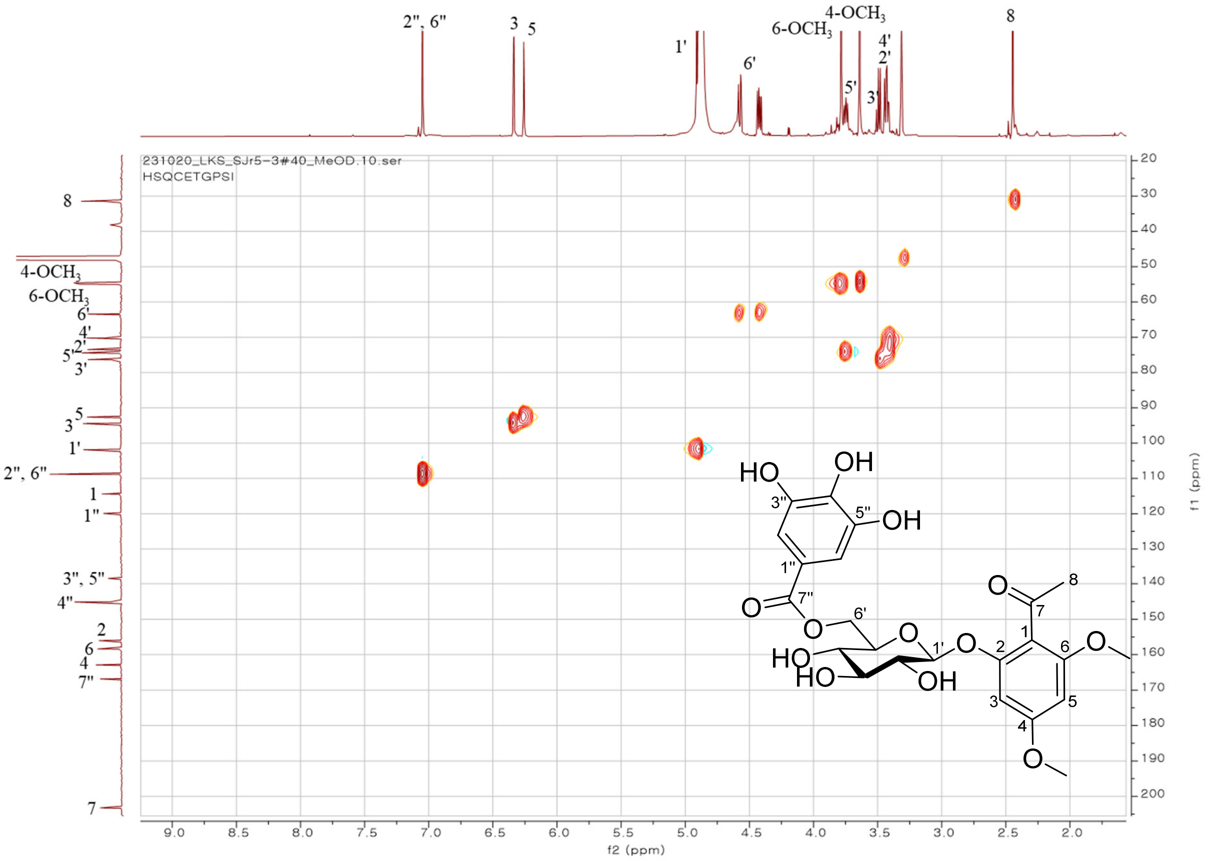

Figure S41. HSQC spectrum of compound **16** (600 and 150 MHz, CD_3OD+D_2O).

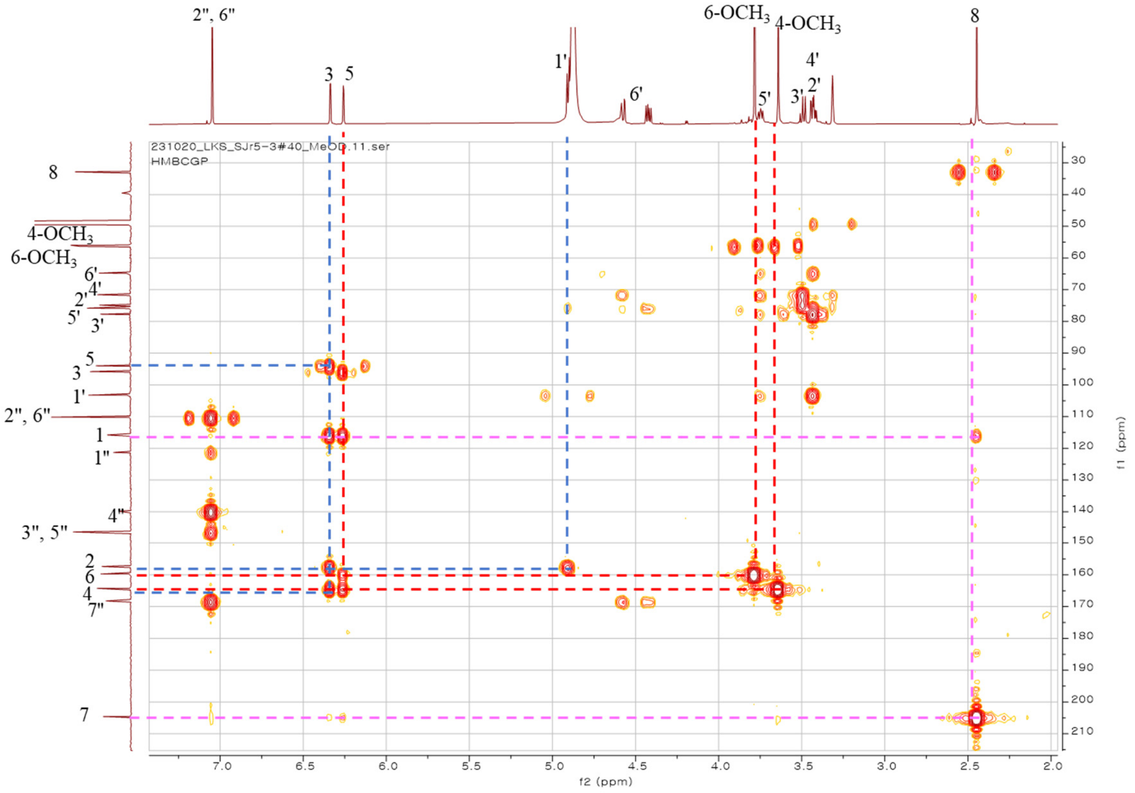

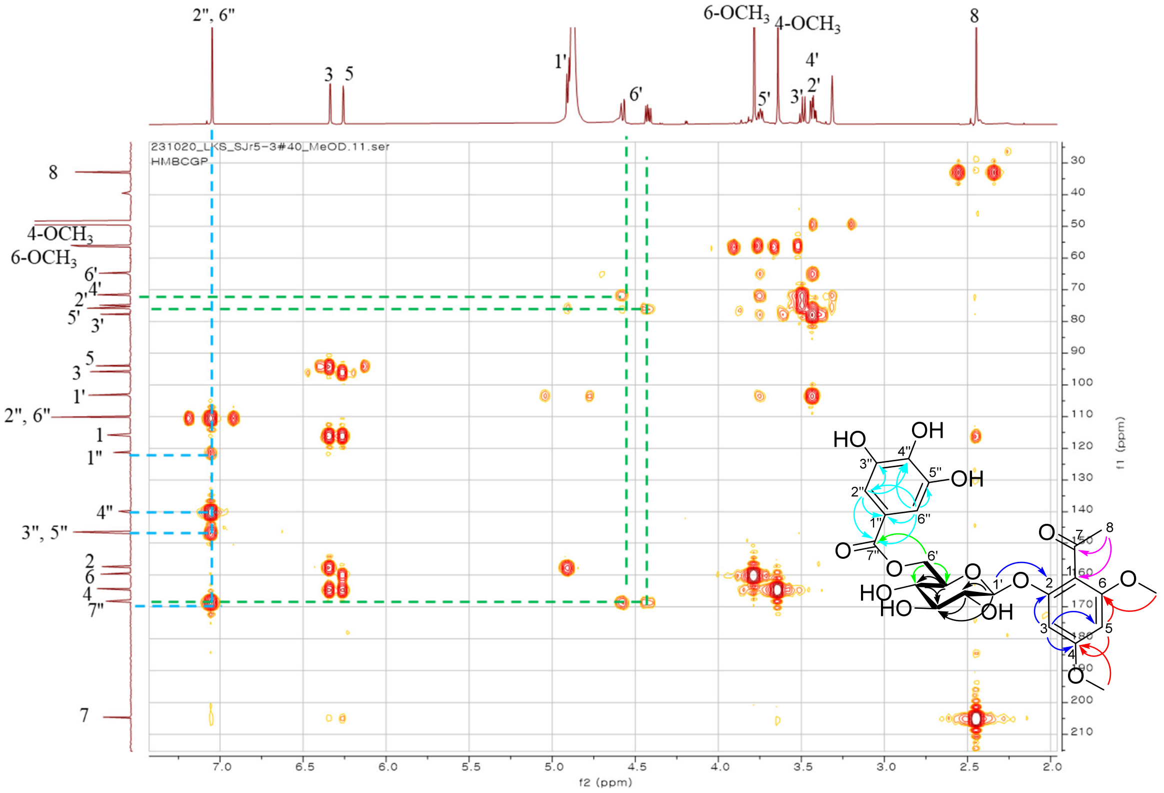

Figure S42. HMBC spectrum of compound **16** (600 MHz, CD_3OD+D_2O).

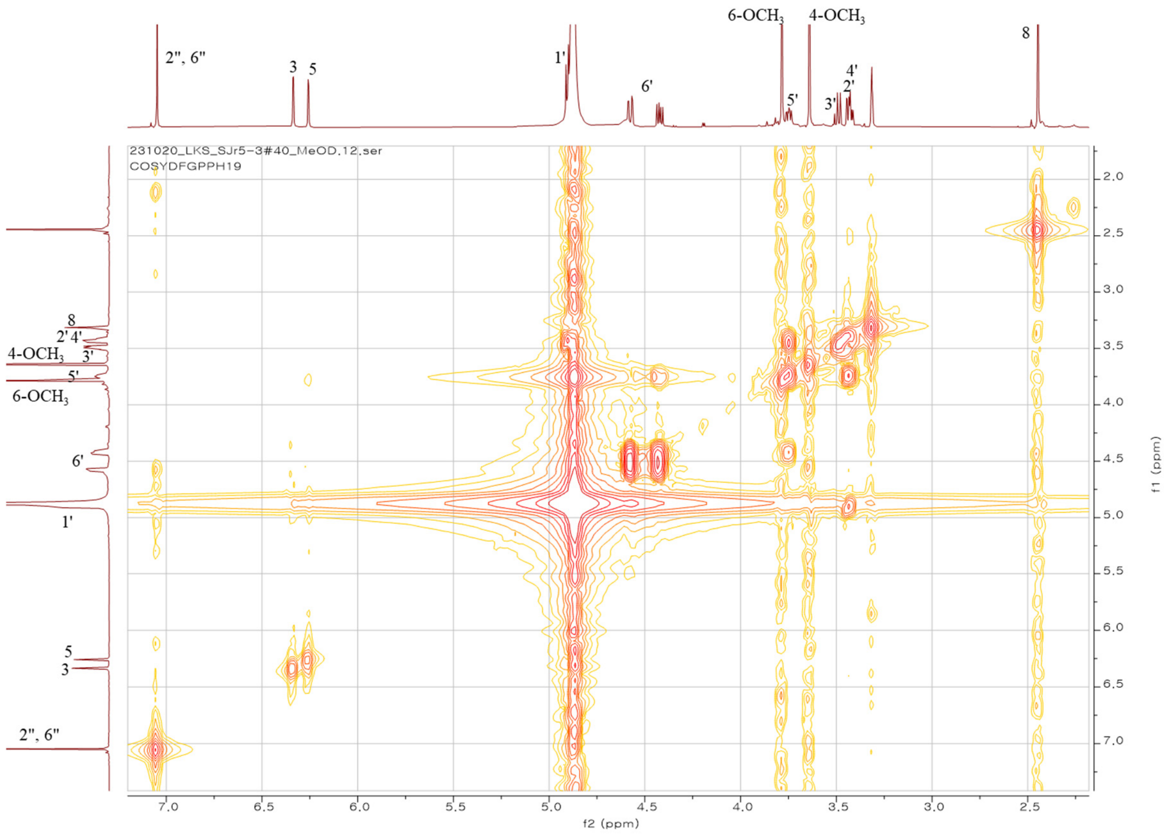

Figure S43. COSY spectrum of compound **16** (600 MHz, CD_3OD+D_2O).

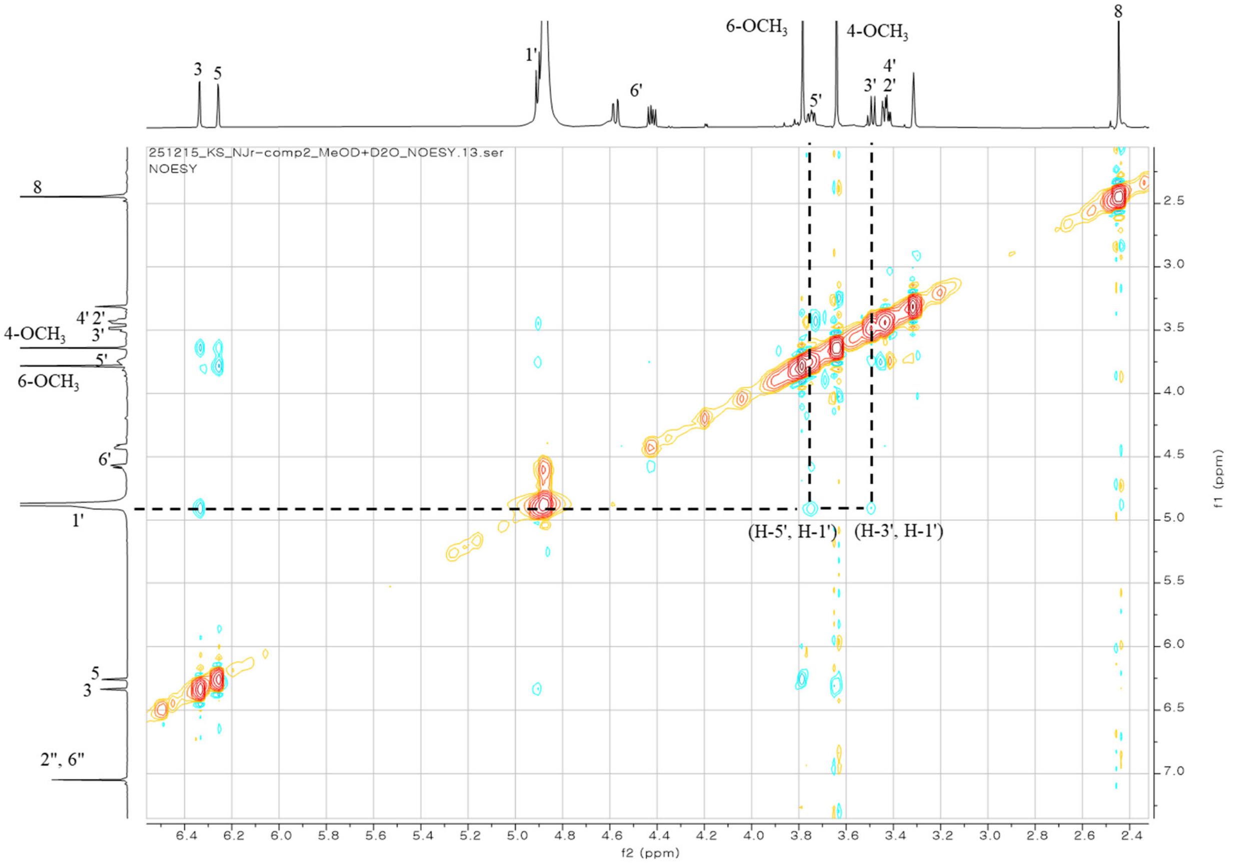

Figure S44. NOESY spectrum of compound **16** (600 MHz, CD_3OD+D_2O).

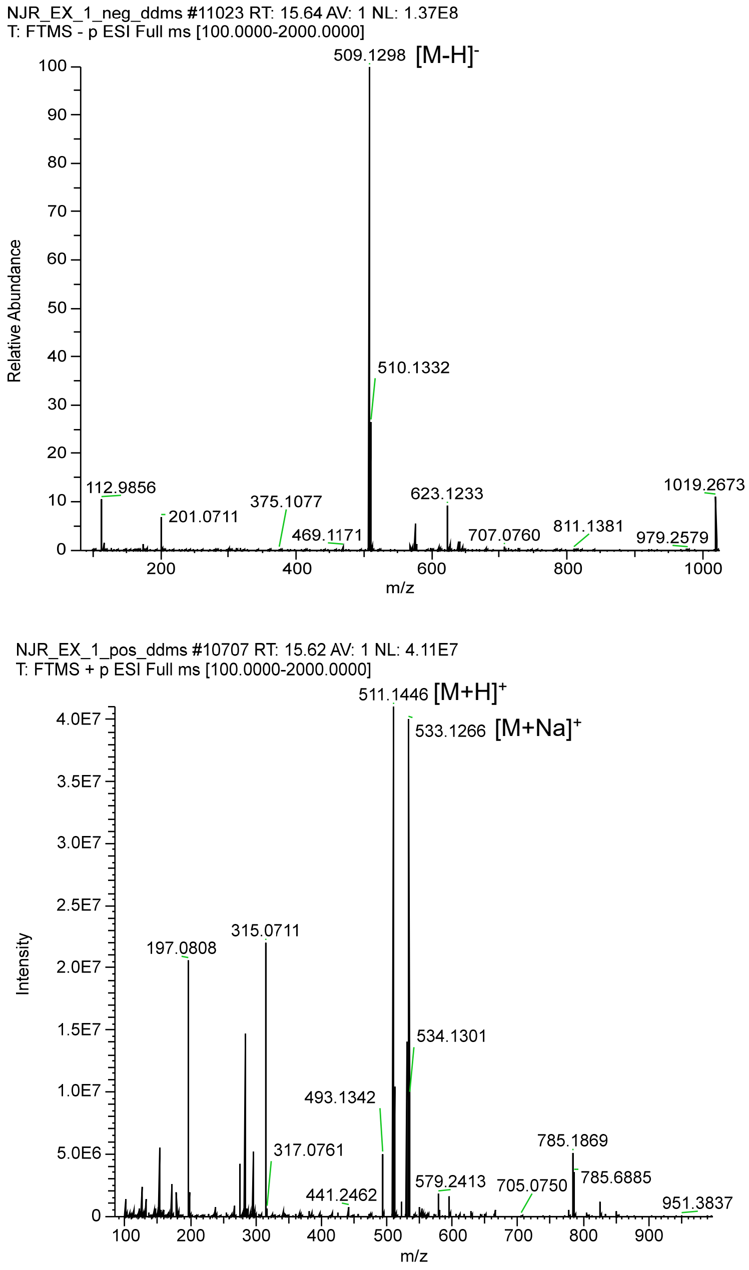

Figure S45. HRESIMS spectra of compound **16.** (above) at negative ion mode; (below) at positive ion mode.

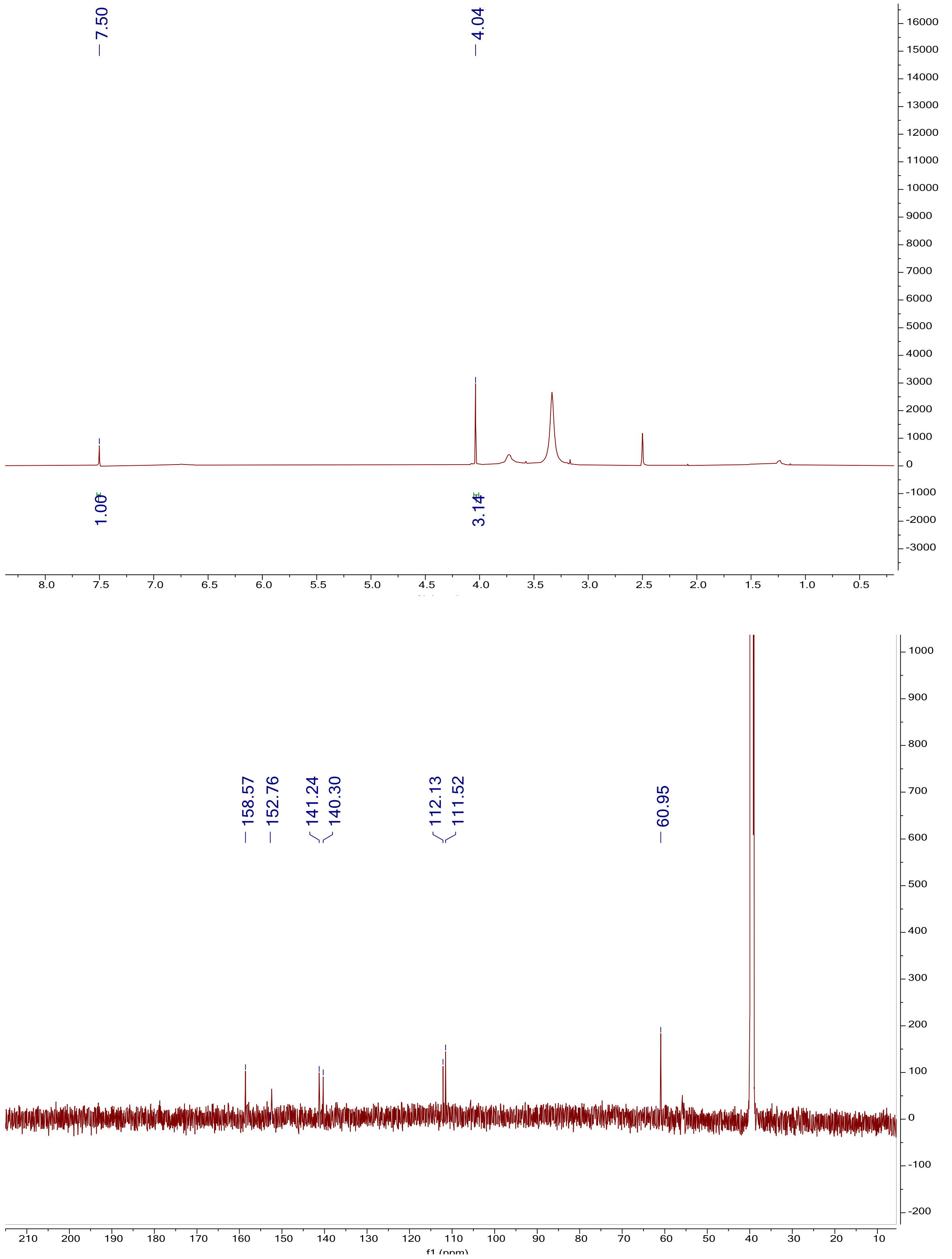

Figure S46. ^{1}H- and ^{13}C-NMR spectra of compound **17** (600 and 150 MHz, DMSO-d_6).

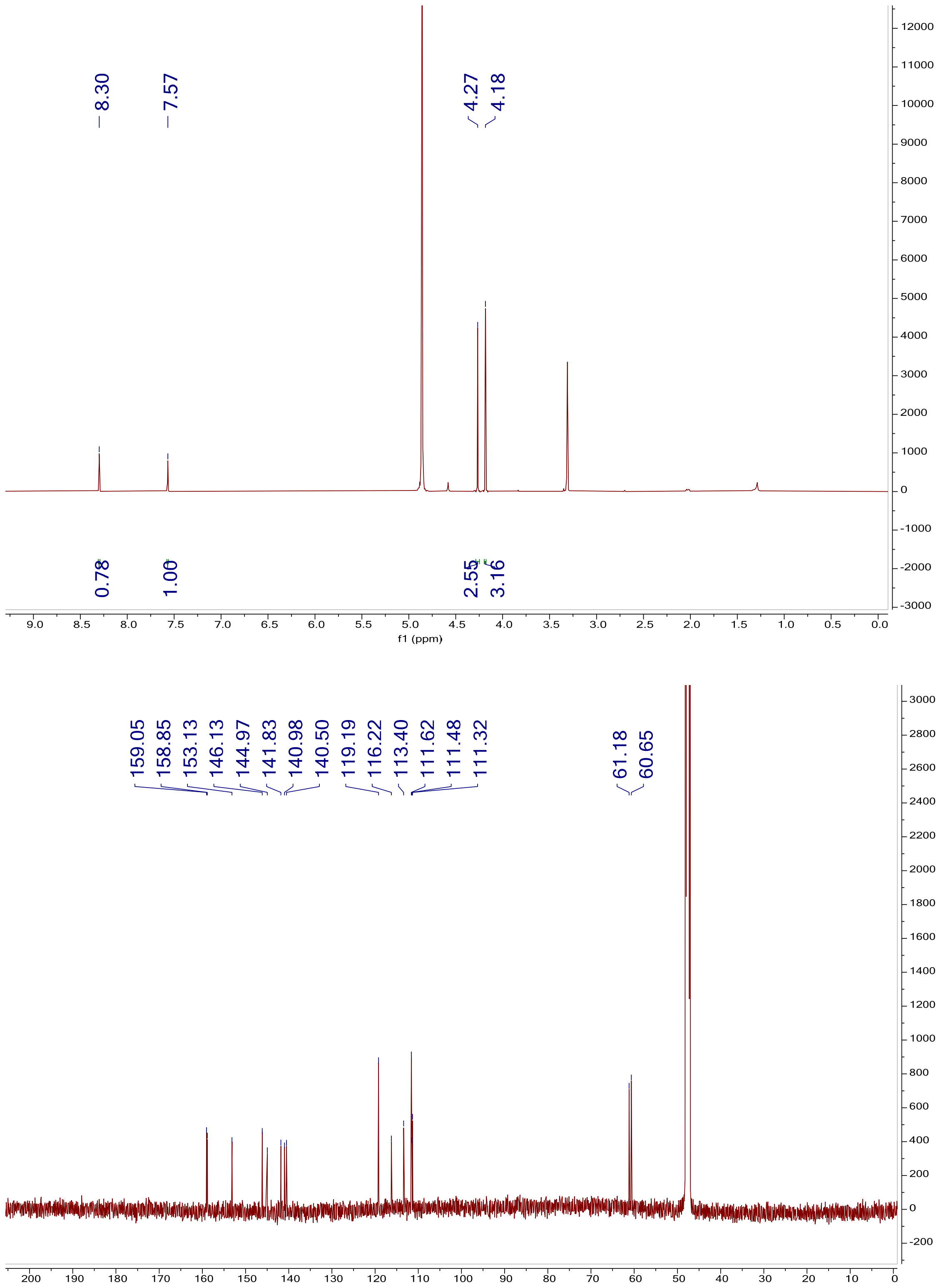

Figure S47. ^{1}H- and ^{13}C-NMR spectra of compound **18** (600 and 150 MHz, CD_3OD).

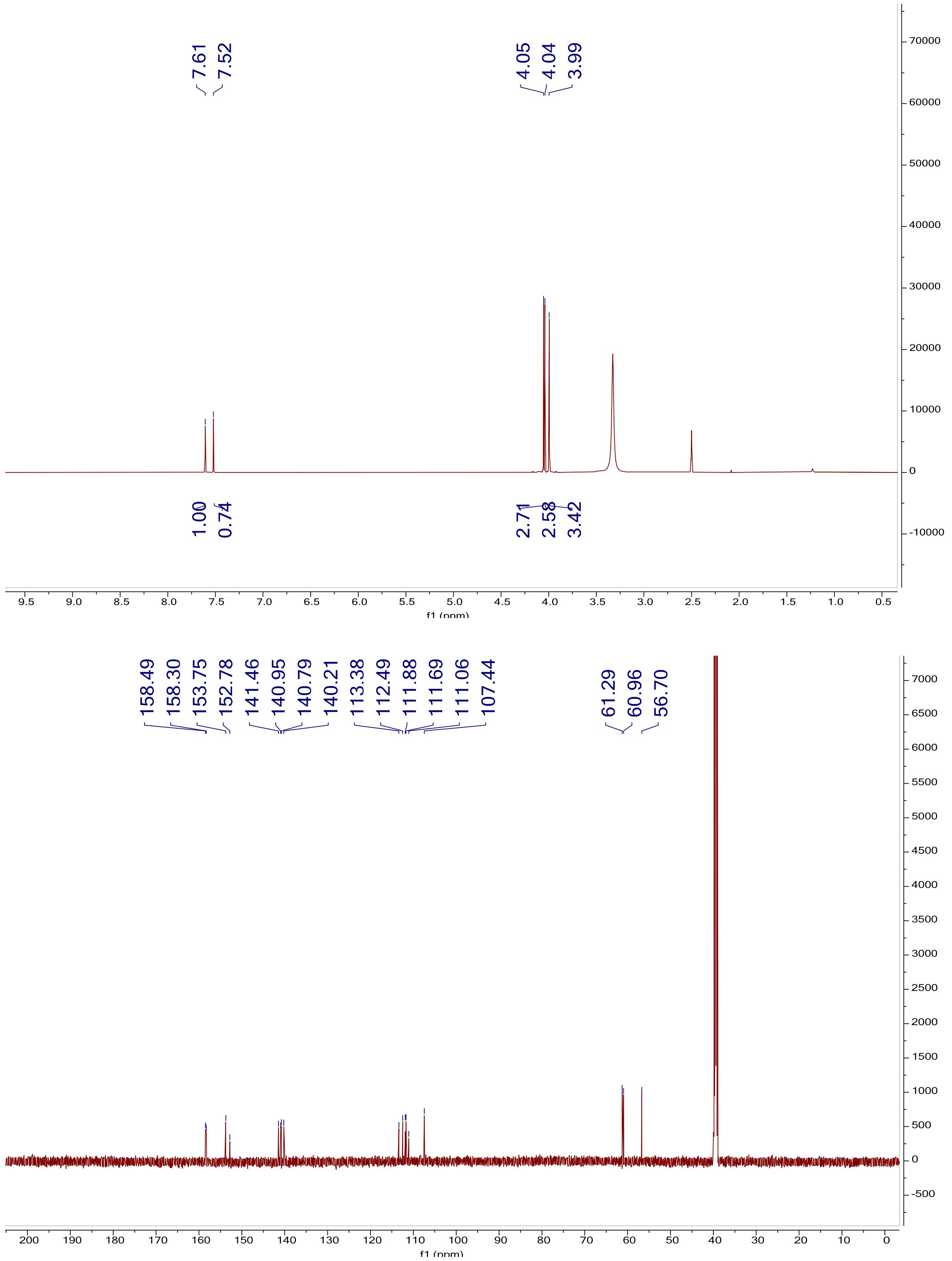

Figure S48. ^{1}H- and ^{13}C-NMR spectra of compound **19** (600 and 150 MHz, DMSO-d_6).

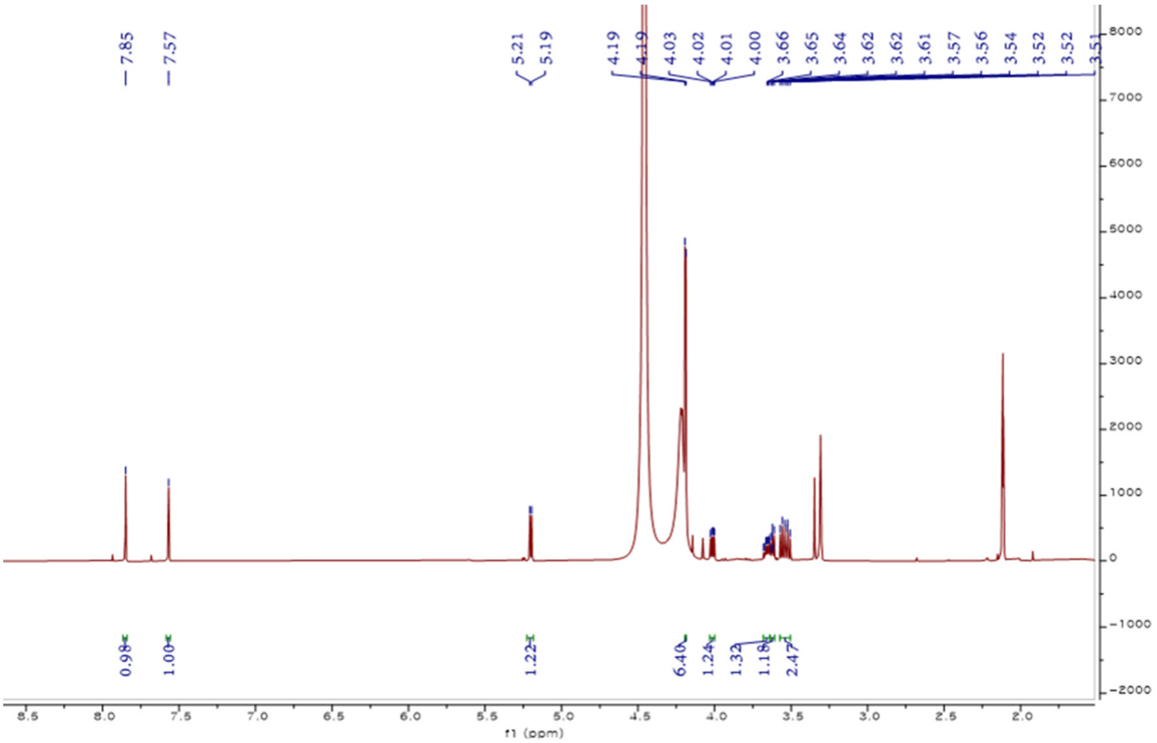

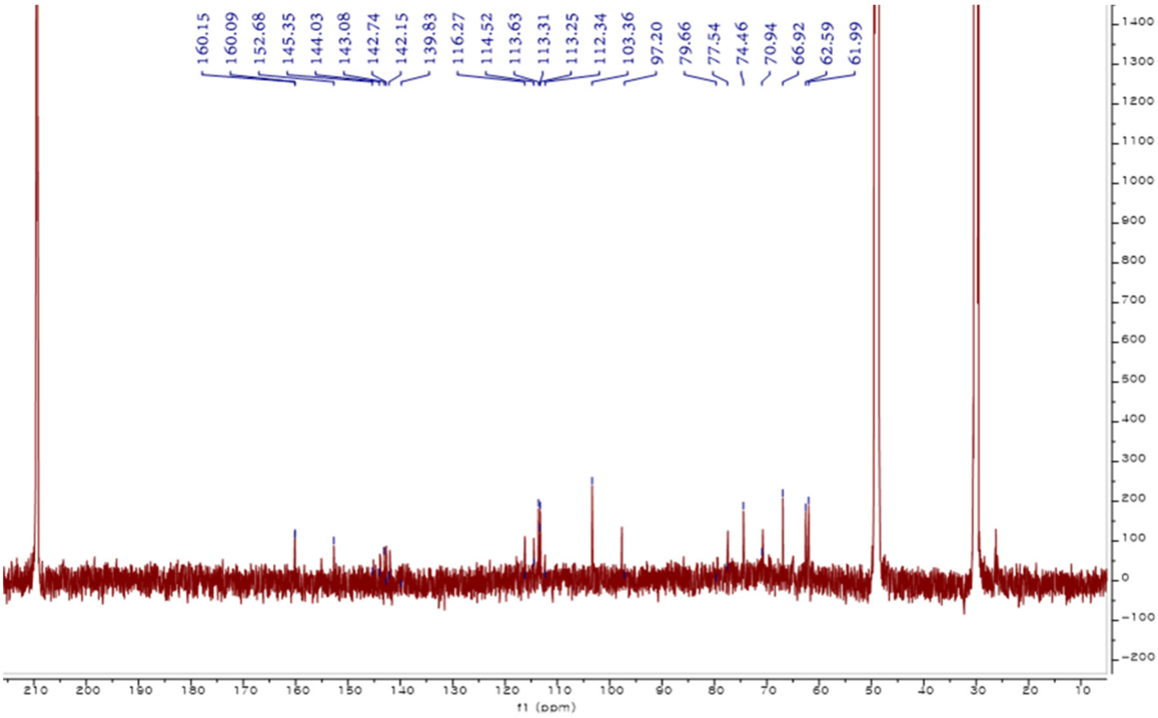

Figure S49. ^{1}H- and ^{13}C-NMR spectra of compound **20** (600 and 150 MHz, CD_3OD:Acetone-d_5 1:1, v/v).
